# High density of unrepaired genomic ribonucleotides leads to Topoisomerase 1-mediated severe growth defects in absence of ribonucleotide reductase

**DOI:** 10.1093/nar/gkaa103

**Published:** 2020-03-18

**Authors:** Susana M Cerritelli, Jaime Iranzo, Sushma Sharma, Andrei Chabes, Robert J Crouch, David Tollervey, Aziz El Hage

**Affiliations:** 1 SFR, Division of Intramural Research, Eunice Kennedy Shriver National Institute of Child Health and Human Development, National Institutes of Health, Bethesda, MD, USA; 2 National Center for Biotechnology Information, National Library of Medicine, National Institutes of Health, Bethesda, MD 20894, USA; 3 Department of Medical Biochemistry and Biophysics, Umeå University, Umeå SE-901 87, Sweden; 4 The Wellcome Centre for Cell Biology, University of Edinburgh, Edinburgh, UK

## Abstract

Cellular levels of ribonucleoside triphosphates (rNTPs) are much higher than those of deoxyribonucleoside triphosphates (dNTPs), thereby influencing the frequency of incorporation of ribonucleoside monophosphates (rNMPs) by DNA polymerases (Pol) into DNA. RNase H2-initiated ribonucleotide excision repair (RER) efficiently removes single rNMPs in genomic DNA. However, processing of rNMPs by Topoisomerase 1 (Top1) in absence of RER induces mutations and genome instability. Here, we greatly increased the abundance of genomic rNMPs in *Saccharomyces cerevisiae* by depleting Rnr1, the major subunit of ribonucleotide reductase, which converts ribonucleotides to deoxyribonucleotides. We found that in strains that are depleted of Rnr1, RER-deficient, and harbor an rNTP-permissive replicative Pol mutant, excessive accumulation of single genomic rNMPs severely compromised growth, but this was reversed in absence of Top1. Thus, under Rnr1 depletion, limited dNTP pools slow DNA synthesis by replicative Pols and provoke the incorporation of high levels of rNMPs in genomic DNA. If a threshold of single genomic rNMPs is exceeded in absence of RER and presence of limited dNTP pools, Top1-mediated genome instability leads to severe growth defects. Finally, we provide evidence showing that accumulation of RNA/DNA hybrids in absence of RNase H1 and RNase H2 leads to cell lethality under Rnr1 depletion.

## INTRODUCTION

In eukaryotes, undamaged nuclear DNA is replicated by three members of the B family of DNA polymerases (Pols), Pol α, Pol ϵ and Pol δ, whose catalytic subunits are Pol1, Pol2 and Pol3, respectively (for a review, see e.g. (1)). Pol α-RNA primase complex initiates synthesis of both leading and lagging strands. On the leading strand, Pol α is then replaced by Pol ϵ, which synthesizes long stretches of DNA in a processive manner. On the lagging strand, Pol δ takes over from Pol α and synthesizes Okazaki fragments (henceforth referred to as ‘OF’), which are short segments of about 200 nt that are processed and ligated after polymerization (for a review, see e.g. (2)). Recent *in vivo* analyses in *Saccharomyces* (*S*.) *cerevisiae* and *Schizosaccharomyces pombe*, and *in vitro* reports, indicate that Pol δ contributes to leading strand synthesis (3–9).

Pols ϵ and δ are extremely accurate in copying the genome and have high substrate selectivity for the base and sugar components of deoxyribonucleoside tri-phosphates (dNTPs). However, the stringency of selection against the incorporation of ribonucleoside monophosphates (rNMPs) varies among replicative Pols α, ϵ and δ ((10); for reviews, see e.g. (11,12)). As cellular rNTP concentrations in eukaryotes are generally one to two orders of magnitude higher than those of the corresponding dNTPs, this potentially affects the frequencies of rNMP incorporation by the replicative Pols (10,13).

In *S. cerevisiae*, rNTP abundances are relatively constant throughout the cell cycle (14). In contrast, the levels of dNTPs increase 3–6-fold during DNA replication in S-phase in normal/unperturbed growth conditions and a further 3–5-fold upon DNA damage (14,15). The ribonucleotide reductase (henceforth referred to as ‘RNR’) complex catalyzes the rate-limiting step in *de novo* dNTP synthesis by reducing ribonucleotides into deoxyribonucleotides and balancing the concentrations of all four dNTPs. In all eukaryotes, the RNR complex is formed by a large subunit R1 that harbors both the catalytic and allosteric sites, and a small subunit R2 that houses the diferric-tyrosyl radical cofactor that is essential for the initiation of nucleotide reduction. In *S. cerevisiae*, R1 is a homodimer formed of two copies of the major catalytic-subunit Rnr1, and R2 is a heterodimer formed of Rnr2 and Rnr4 (for a review of yeast RNR complex, see e.g. (16)). The expression, activity and localization of the yeast RNR complex are exquisitely regulated during the cell cycle in unperturbed cells, and also in conditions of DNA damage and replicative stress (henceforth, these two conditions are collectively referred to as ‘genotoxic stress’) (see Supplementary Figure S1). Notably, Rnr3, the minor catalytic-subunit of the yeast RNR complex, is expressed at low levels during the cell cycle in unperturbed cells, but is highly upregulated under genotoxic stress (17).

Single ribonucleotides incorporated in nuclear DNA by Pols can be removed by the error-free Ribonucleotide Excision Repair (RER) pathway. This is initiated by RNase H2, which incises at the scissile phosphate upstream of the rNMP, thereby creating a nick whose ends have a 3′ OH and a 5′ RNA-DNA junction. The 3′ OH end is subsequently extended by the OF maturation machinery ((18); for a review see e.g. (12)). Genomic ribonucleotides that accumulate in absence of RNase H2 are henceforth referred to as ‘unrepaired rNMPs’. Loss of RNase H2-dependent-RER causes DNA damage that leads to embryonic lethality in mice (19–21), but is tolerated in *S. cerevisiae* (see e.g. (22,23)). RNase H1, the other major RNase H in eukaryotes, does not play a role in RER (18), as it needs at least four contiguous rNMPs in DNA for cleavage (for reviews, see e.g. (24,25)). However, both RNase H1 and RNase H2 (henceforth both enzymes are referred to as ‘RNases H1 and H2’) can process the RNA moiety of RNA/DNA hybrids (henceforth referred to as ‘hybrid-removal activity’; for reviews, see e.g. (24,25)), which can be found as part of R-loops in the nuclear and mitochondrial genomes (for R-loops in *S. cerevisiae*, see e.g. (26–28)). Notably, transcription-associated-R-loops can block replication fork (henceforth referred to as 'RF') progression, thereby threatening the stability of the genome (for reviews, see e.g. (29,30)).

Single genomic rNMPs can also be cleaved by DNA topoisomerase 1 (Top1), particularly unrepaired rNMPs. Top1 incises the scissile phosphate downstream of a single rNMP in duplex DNA, which could lead to an un-ligatable ribonucleoside-2′, 3′ cyclic phosphate-terminated end (henceforth referred to as ‘un-ligatable nick’; for reviews, see e.g. (12,31)). The nick could be reversed by Top1 (32,33), or be repaired by Apn2 and Srs2-Exo1 pathways (34,35). Alternatively, Top1 could make a second incision in the same strand, upstream of the un-ligatable nick, thereby resulting in a short gap, which can either be filled by error-free repair pathways (33), or lead to a deletion of 2–5 bp if the incision occurs within a short tandemly repeated sequence (henceforth the 2–5 bp deletion is referred to as ‘Δ2–5 bp’; see e.g. (32,33,36)). Another possibility is that Top1 could make a second incision in the complementary strand, opposite to the un-ligatable nick, thereby creating a DNA double strand break (DSB) that can either be repaired by the cellular Rad51/52-dependent homologous recombination (HR) machinery, or lead to Top1-mediated illegitimate recombinations (37). Top1-mediated DNA damage at sites of single genomic rNMPs is henceforth referred to as ‘Top1-mediated RNA-DNA damage’.

In this study, we sought to deplete Rnr1 in *S. cerevisiae* in order to analyze the consequences of reduced dNTP pools on genome integrity and cell viability of mutants lacking RNase H1, RNase H2, or both enzymes. We found that the removal of RNA/DNA hybrids by RNases H1 and H2 is essential for the growth of Rnr1-depleted cells. Importantly, we found that single genomic rNMPs are highly enriched in double mutants lacking both Rnr1 and RNase H2. This was further exacerbated in triple mutants that are depleted of Rnr1, lack RNase H2, and also harbor a steric gate replicative Pol variant with reduced discrimination against utilization of rNTPs as compared to its WT parent enzyme (henceforth referred to as ‘rNTP-permissive Pol’). Furthermore, our Southern blotting data led us to infer that, in cells depleted of Rnr1 and lacking RNase H2, Top1-mediated cleavages occur in both the leading and lagging strands when rNMPs are excessively incorporated by an rNTP-permissive form of Pol δ or α; but only in the leading strand by an rNTP-permissive form of Pol ϵ. Accordingly, triple mutants that are depleted of Rnr1, lack RNase H2, and harbor an rNTP-permissive form of Pol ϵ or δ showed severe growth defects that are likely to be caused by deleterious Top1-mediated RNA-DNA damage. Finally, we provide evidence to support the proposed role of Pol δ in leading strand synthesis (here particularly observed under replicative stress induced by Rnr1 depletion), in addition to its major role in lagging strand synthesis.

## MATERIALS AND METHODS

### Strains and plasmids

Yeast strains (BY4741 background) and plasmids used in this study are listed in Supplementary Table S1. Yeast transformations were carried out using a standard lithium acetate/polyethylene glycol protocol (38). Plasmids ‘pFA6a-*HIS3*MX6-*P_GAL1_-3HA*’ and ‘pFA6a-kanMX6/NatMX6/HphMX6’ were used for the construction of ‘*P_GAL_:3HA-RNR1*’ and ‘gene deletions’, respectively (39). Plasmids p173-Pol2-M644G (for *pol2-M644G*), p173-Pol2-M644L (for *pol2-M644L*), PYIAL30-L868M (for *pol1-L868M*) and p170-LM (for *pol3-L612M*) were used for the construction of the four Pol mutant alleles (generous gift from Jessica Williams, lab of Thomas Kunkel, National Institute of Environmental Health Sciences, NIH).

### Drop test growth assays

Strains harboring the *RNR1* gene under the control of its native promoter were pre-grown in liquid medium containing YEPD (medium contains 1% yeast extract [Formedium YEA02], 2% bacto-peptone [Formedium PEP02], and 2% dextrose [Sigma-Aldrich D9434]). Cells were spotted on solid medium containing either YEPD with 2% agar (Formedium AGA02), or YPGS with 2% agar (composition of YPGS is as for YEP but supplemented with 2% galactose [Sigma-Aldrich G0750] and 1% sucrose [Sigma-Aldrich 84097]). Strains carrying *P_GAL_:3HA-RNR1* were pre-grown in liquid medium containing YPGS, which is permissive for Rnr1 expression. Cells were spotted on solid medium containing either YEPD, which is non-permissive for Rnr1 expression, or YPGS. Strains carrying *P_GAL_:3HA-RNR1* together with a plasmid were pre-grown in liquid minimal medium lacking leucine with 2% galactose and 1% sucrose, which is permissive for Rnr1 expression (medium contains SD-Leu-Glucose [Sunrise Science Products 1799; ‘SD-Leu-Glucose’ stands for ‘synthetic defined minus leucine minus glucose’], galactose and sucrose). Cells were spotted on solid minimal medium lacking leucine, with either 2% glucose, which is none-permissive for Rnr1 expression (medium contains SD-Leu [Sunrise Science Products 1707; glucose included]), or 2% galactose and 1% sucrose. 10-fold dilutions of overnight, saturated liquid cultures were spotted on the plates, starting from 0.4 OD_600_ of yeast cells.

### *CAN1* forward mutation assay

*CAN1* forward mutation assay was performed according to (40), with modifications. Briefly, strains were streaked out on solid medium containing either YEPD (for strains WT and *rnh201Δ*), or YPGS (for strains *P_GAL_:3HA-RNR1* and *P_GAL_:3HA-RNR1 rnh201Δ*). For composition of YEPD and YPGS media see section ‘Drop test growth assays’. Plates were incubated for 2 days at 30°C until single colonies appeared. Then, 12–20 patches were made on solid medium containing YEPD (1 single colony per patch). After incubation at 30°C for 24 h (for strains WT and *rnh201Δ*), or 48 h (for strains *P_GAL_:3HA-RNR1* and *P_GAL_:3HA-RNR1 rnh201Δ*), each patch was re-suspended in 500 μl sterile water. To detect Can resistant (Can^R^) colonies, an aliquot from the cell suspension was plated on solid media supplemented with 60 mg l^−1^l-canavanine (Sigma-Aldrich C9758), as follows: (i) For strains WT and *rnh201Δ*, 200 μl cells were plated on minimal medium lacking arginine with 2% glucose (DOBA [Sunrise Science Products 1651; this medium contains glucose] and CSM-Arg [Sunrise Science Products 1031]). (ii) For strains *P_GAL_:3HA-RNR1* and *P_GAL_:3HA-RNR1 rnh201Δ*, 200 and 100 μl cells, respectively, were plated on minimal medium lacking arginine with 2% galactose and 1% sucrose (DOBA-glucose w/2% galactose [Sunrise Science Products 1653; this medium does not contain glucose], CSM-Arg and sucrose). For each strain, four independent experiments were performed, each including 12–20 patches. Total mutation rates and 95% confidence intervals were calculated for each independent experiment by the Lea and Coulson method of median (41,42), using a template kindly provided by Nayun Kim (University of Texas Health Science Center at Houston) (see (42)). To determine *CAN1* specific mutation rates, one Can^R^ colony was randomly picked from each plate and re-suspended in 50 μl water. A 20 μl aliquot of cell suspension was used for PCR amplification of *CAN1* using Herculase II Fusion DNA polymerase (Agilent Technologies 600679). For primers used for PCR and sequencing of *CAN1* see Supplementary Table S2. Specific mutation rates were calculated according to (43).

### Growth conditions for Rnr1 depletion in liquid media

**Figure 1.**
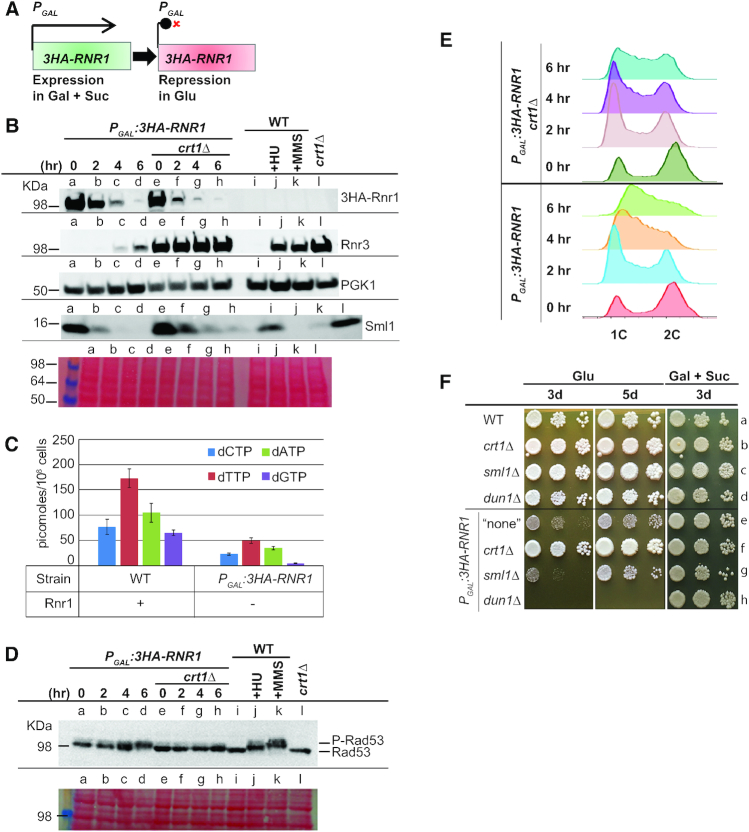
Depletion of Rnr1 in BY4741 background mildly triggers the S-phase checkpoint, greatly reduces dNTP levels and significantly prolongs S-phase. (**A**) Cartoon depicting the gene *RNR1* under the control of the inducible *P_GAL1/10_* promoter. The promoter is induced in galactose plus sucrose (gal + suc) media and inhibited in glucose (glu) media. Rnr1 is epitope-tagged with 3x hemagglutinin (3HA) at its N-terminus. (**B**) Rnr3 protein is mildly expressed in single mutant *P_GAL_:3HA-RNR1* depleted of Rnr1. Strains *P_GAL_:3HA-RNR1* and *P_GAL_:3HA-RNR1 crt1Δ* were grown at 30°C in liquid minimal medium lacking histidine with 2% galactose and 2% sucrose. To trigger Rnr1 depletion, cells at OD_600_ ∼0.2 were transferred to liquid minimal medium lacking histidine with 2% glucose. Cells were harvested before transfer (0 h) and 2, 4 and 6 h after transfer to glucose-containing medium (see also Materials and Methods). Strain WT was grown in rich YEPD (2% glucose) medium at 30°C, in absence of drugs (i.e. unperturbed conditions), or for 3 h in presence of either 200 mM HU (labeled +HU) or 0.03% MMS (labeled +MMS) (see also Materials and Methods). Strain *crt1Δ* was grown in rich YEPD (2% glucose) medium at 30°C. Note that all cell samples were harvested in exponential phase. Total proteins were separated on a 4–20% SDS-polyacrylamide gel and then electro-blotted. The filter was stained with Ponceau Red (bottom sub-panel). The same filter was probed separately with antibodies against HA tag (3HA-Rnr1), Rnr3, PGK1 and Sml1. Relevant protein molecular weights (KDa) are indicated at the left-hand. For the ease of comparison, each well is allocated a unique Latin letter, which is repeated in each sub-panel. The length of Rnr1 depletion in hours (hr) is indicated above the wells a-d and e-h. One representative experiment is shown of at least three independent ones. (**C**) dNTP levels, particularly dGTPs, are greatly decreased in single mutant *P_GAL_:3HA-RNR1* depleted of Rnr1. The WT strain was cultured in rich YEPD medium (2% glucose) at 30°C and harvested at OD_600_ ∼0.4. The single mutant *P_GAL_:3HA-RNR1* was cultured as explained in (B) and harvested 6 h after transfer to glucose-containing media at OD_600_ ∼0.4. dNTP levels were normalized to rNTP levels and values were adjusted to the total number of cells used for the preparation. Error bars reflect S.E.M. of 2 independent repeats. Symbols on the organigram: + and – indicate that Rnr1 is present or absent, respectively. For the ease of comparison, WT strain and single mutant *P_GAL_:3HA-RNR1* are also represented in Supplementary Figure S3A (see also Supplementary Table S4). (**D**) Rad53 is mildly phosphorylated in single mutant *P_GAL_:3HA-RNR1* depleted of Rnr1. Strains and growth conditions are as in (B). Total proteins were separated on a 6.5% SDS-polyacrylamide gel. The Filter was probed with antibody against Rad53 (P-Rad53 represents the phosphorylated form of Rad53). For other details, see (B). (**E**) Cells depleted of Rnr1 accumulate significantly in S-phase in the presence of Crt1. Flow cytometry (FACS) histograms of cells from strains *P_GAL_:3HA-RNR1* and *P_GAL_:3HA-RNR1 crt1Δ*, before (0 h), and 2, 4 and 6 h after transfer to glucose medium. One representative experiment is shown of at least three independent ones. Note that cell samples from the same cultures were used for FACS, western-blotting in (B) and (D), and RT-qPCR in Supplementary Figure S2. (**F**) Cells depleted of Rnr1 grow much slower than the WT strain, but their growth is fully restored in the absence of Crt1. Drop test growth assays of strain WT, strains deleted of the gene *CRT1*, *SML1*, or *DUN1*, and strains carrying *P_GAL_:3HA-RNR1* without gene deletion (labeled “none"), or with deletion of the gene *CRT1*, *SML1* or *DUN1*. Cells were pre-grown in YPGS (2% galactose and 1% sucrose) liquid medium overnight. Serial dilutions were plated on YEPD (2% glucose) and YPGS solid media. Plates were incubated at 25°C. Photographs were taken at the indicated number of days (d). For the ease of comparison, a unique Latin alphabet letter is allocated for each row. ‘Glu’ stands for glucose and ‘Gal + Suc’ stands for galactose plus sucrose. The horizontal line across the images is included for clarity. See Supplementary Table S1 for the list of strains. One representative experiment is shown of at least three independent ones.

Day1: Strains carrying *P_GAL_:3HA-RNR1* were pre-grown overnight at 30°C in liquid minimal medium lacking histidine with 2% galactose and 2% sucrose, which is permissive for Rnr1 expression (medium contains yeast nitrogen base without amino acids and with ammonium sulphate [Formedium CYN0410], synthetic complete mixture Kaiser drop-out -His [Formedium DSCK1003], galactose [Acros Organics 59-23-4], and sucrose [Fisher 57-50-1]). Day 2: In the morning, saturated pre-cultures were diluted with the same medium to OD_600_ ∼0.05. Growth was maintained in exponential phase by dilution with the same pre-warmed medium for 24 h. Day 3: In the morning, cells were harvested at OD_600_ ∼0.2, then washed with pre-warmed liquid minimal medium lacking histidine with 2% glucose, which is non-permissive for Rnr1 expression (same medium composition as above but supplemented with glucose [Fisher 50-99-7] as the sole carbon source). Cells were subsequently re-suspended in the same pre-warmed medium containing glucose to OD_600_ ∼0.1–0.2. Growth was maintained in exponential phase by dilution with the same pre-warmed medium containing glucose. Cells were collected at the indicated time points. Note that for Figure 1B, D and E, and Supplementary Figures S2, S3B and S8-S10, an aliquot of exponentially growing cells was also collected from medium containing galactose plus sucrose, in which Rnr1 is moderately over-expressed.

### Growth conditions in absence or presence of drugs in liquid media

WT cells (BY4741) were pre-grown overnight in YEPD medium at 30°C (for composition of YEPD medium see section ‘Drop test growth assays’). The next morning, saturated pre-cultures were diluted to OD_600_ ∼0.05 in the same medium. When OD_600_ reached ∼0.3, cells were split in three portions: one for control in absence of drugs, one for treatment with 200 mM hydroxyurea (HU; Acros Organics 127-07-1), and one for treatment with 0.03% methyl methane sulfonate (MMS; Sigma 129925). Control cells were harvested at OD_600_ ∼0.5–0.6. Cells treated with HU or MMS were kept in exponential phase by dilution in the same medium and finally harvested at OD_600_ ∼0.5–0.6 after 3 h in presence of the drug.

### Western-blotting

Total protein extraction from ∼5 OD_600_ of yeast cells was performed by NaOH lysis and trichloroacetic acid (TCA) precipitation according to (44), with minor modifications. Dissolved cell pellets (50 μl) were heated at 95°C for 10 min, and then spun for 10 min at 10 000 g at room temperature. Protein extracts (10% volume of the supernatant) were then resolved, together with a protein ladder (SeeBlue Plus2 Pre-stained Standard, ThermoFisher Scientific LC5925), by SDS-PAGE (4–20% Mini-Protean TGX Precast gel [Bio-Rad 456-1096] in Figure 1B, and standard 6.5% SDS-polyacrylamide gel in Figure 1D and Supplementary Figure S3B). Proteins were electro-transferred from the gel onto a nitrocellulose membrane (Thermo Fisher Scientific 88018). The membrane was sequentially treated as follows: Step 1: Stained briefly with Ponceau Red and then washed with distilled water. Step 2: Blocked for 30 min at room temperature in 1× PBS plus 0.1% tween (referred to as ‘PBST’; 1× PBS contains 137 mM NaCl, 2.7 mM KCl, 8 mM Na_2_HPO_4_ and 2 mM KH_2_PO_4_) with 5% (w/v) milk (Skim milk powder, OXOID LP0031). Step 3: Incubated with primary antibody in PBST with 5% milk for 1 h at room temperature followed by overnight incubation at 4°C. Step 4: Washed with PBST for 3 × 10 min at room temperature. Step 5: Incubated with secondary antibody in PBST with 5% milk for 1 h at room temperature. Step 6: Washed with PBST for 3 × 10 min at room temperature. Step 7: Treated with ECL Western Blotting substrate (Thermo Fisher Scientific/Pierce 32106). Note that for successive incubations with various sets of primary/secondary antibodies in Figure 1B and Supplementary Figure S3B, the blot was stripped (Restore western blotting stripping buffer, Thermo Fisher Scientific 21059) for 30 min at 30°C with shaking, then washed briefly with PBST, then incubated with another set of primary/secondary antibodies. The following primary antibodies were used: (i) HA-probe (F-7) HRP at 1:2500, for the detection of 3HA-Rnr1 (mouse monoclonal, Santa Cruz sc-7392). (ii) Anti-Rnr3 at 1:1500 (rabbit polyclonal, Agrisera AS09 574). (iii) Anti-Sml1 at 1:2500 (rabbit polyclonal, Agrisera AS10 847). (iv) Anti-PGK1 at 1:5000 (mouse monoclonal, Thermofisher Scientific 22C5D8). (v) Anti-Rad53 (yC-19) at 1:500 (goat polyclonal, Santa Cruz sc-6749 [this product has now been discontinued and replaced by Rad53 (A-9): sc-74427]). (vi) Anti-RNAPII at 1:5000 (mouse monoclonal, Diagenode C15200004 [against the C-terminal heptapeptide of RNA polymerase II largest subunit RPB1]). The following secondary antibodies were used: (i) Anti-rabbit-HRP at 1:10 000 (donkey polyclonal, GE healthcare NA934). (ii) Anti-mouse-HRP at 1:10 000 (sheep polyclonal, GE healthcare NXA931). (iii) Anti-goat-HRP at 1:2500 (donkey polyclonal, Santa Cruz sc-2020 [this product has now been discontinued and replaced by sc-2354]).

### Fluorescence-activated cell sorting (FACS) analysis

FACS was performed essentially as described in (45), with minor modifications. Propidium-iodide-stained cells were sonicated for 2 × 10 s at 4°C (Sonicator Bioruptor PICO, Diagenode) and subsequently analyzed using a flow cytometer. DNA profiles were generated using the FLOWJO software.

### Reverse transcription of total RNA combined with quantitative PCR (RT-qPCR)

Total RNA was extracted from ∼10–15 OD_600_ of yeast cells, as follows: Step 1: The cell pellet was vortexed vigorously in presence of 100 μl GTC-phenol (2.11 M guanidine thiocyanate, 26.5 mM Tris–HCl pH8, 5.3 mM EDTA pH8, 1.06% *N*-lauroylsarcosine, 75 mM β-mercaptoethanol and 50% phenol [Sigma P4557]) and 100 μl zirconia/silica beads (0.5 mm diameter, Thistle Scientific 11079105z), for 5 min at 4°C. Step 2: 700 μl GTC-phenol were added and the whole mixture was vortexed briefly, then incubated at 65°C for 5 min, and subsequently cooled down on ice. Step 3: 120 μl of 0.1 M NaOAc mix (99 mM NaOAc pH5.2, 10 mM Tris–HCl pH8 and 1 mM EDTA pH8) and 350 μl of chloroform:isoamyl alcohol (24:1 v/v) were added and the whole mixture was vortexed vigorously for 20 s, and then spun at 16 000 g at 4°C for 10 min. Steps 4 and 5: The upper phase was extracted once with an equal volume of phenol:chloroform:isoamyl alcohol (25:24:1 v/v/v; phenol, Sigma P4557), followed by one extraction with chloroform:isoamyl alcohol. Step 6: 450 μl (from the upper phase) were mixed with 1 μl glycoblue co-precipitant (Thermofisher Scientific AM9515) and 1 ml 100% ethanol, then incubated for 1 h at −80°C, and subsequently spun as described above. Step 7: The RNA pellet was washed once with 1 ml 70% ethanol, then ‘air-dried’, and finally re-suspended in distilled water.

RT-qPCR reactions were performed as follows: Step 1: To digest genomic DNA, an aliquot of total RNA (∼30 μg) was incubated with 7 u RQ1 DNase (Promega M6101) and 40 u ribonuclease inhibitor RNasin (Promega N251 A), in a total volume of 60 μl, at 37°C for 30 min. DNA-free RNA was extracted with phenol:chloroform:isoamyl alcohol as described above in steps 4-7. Step 2: An aliquot of DNA-free total RNA (∼1 μg) was incubated with 1 μl of 0.2 μg μl^−1^ random hexamers (Thermofisher Scientific SO142) and 1 μl of 10 mM dNTPs (equimolar mixture of dATP, dCTP, dGTP and dTTP), in a total volume of 14.25 μl, at 65°C for 5 min, and subsequently cooled down on ice. Step 3: For RT reaction, the mixture from the previous step was incubated with 100 u Superscript III Reverse Transcriptase (Thermo Fisher Scientific 18080093), 1× first-strand buffer, 5 mM DTT and 10 u RNasin, in a total volume of 20 μl, at 25°C for 15 min, and then incubated at 50°C for 1 h. RT reaction was stopped by heating at 70°C for 15 min. Step 4: qPCR reactions were performed in triplicate, using 4 μl of 10-fold dilution of complementary DNAs (from previous step) with 1× SYBR premix (TB Green® Premix Ex Taq™ II [Tli RNase H Plus], Takara Bio Europe RR820W) and 0.4 μM primers, in a total volume of 10 μl, as described previously (46). For primer sequences see Supplementary Table S2. To generate RT-qPCR data, the average of triplicates of Ct values was used in the formula ΔΔCt = 2(Ct ‘target mRNA’ – Ct ‘*ACT1* mRNA control’).

### Measurement of dNTP and rNTP levels

Measurement of nucleotide pools was performed as described in (47). Briefly, cells at OD_600_ ∼0.4 were rapidly (<3 min) harvested by filtration (MF-Millipore Membrane Filter, mixed cellulose esters, 0.8 μm, Sigma-Aldrich/Merck AAWP02500), for a total yield of ∼7.4 × 10^8^ cells. rNTPs and dNTPs were extracted in an ice-cold mixture of 12% (w/v) TCA and 15 mM MgCl_2_, and neutralized with an ice-cold freon–trioctylamine mixture (10 ml of freon [1,1,2-trichloro-1,2,2-trifluoroethane], Millipore Sweden AB [>99%], and 2.8 ml of trioctylamine, Sigma-Aldrich Sweden AB [98%]). 575 μl of the aqueous phase were pH adjusted with 1 M ammonium carbonate (pH 8.9), loaded on boronate columns (Affi-Gel Boronate Gel, Bio-Rad), and eluted with 50 mM ammonium carbonate (pH 8.9)- 15 mM MgCl_2_ mix to separate dNTPs from rNTPs. The purified dNTP eluates were adjusted to pH 3.4 and analyzed by HPLC on a LaChrom Elite^®^ HPLC system (Hitachi) with a Partisphere SAX HPLC column (Hichrome, UK). rNTPs were directly analyzed by HPLC in a similar way as dNTPs by using 24 μl aliquots of the aqueous phase, adjusted to pH 3.4.

### Detection of genomic ribonucleotides by alkaline-gel electrophoresis combined with Southern hybridization

Detection of ribonucleotides in genomic DNA by alkaline-gel electrophoresis was performed according to (48), with some modifications. Briefly, total DNA was extracted from ∼50–100 OD_600_ of yeast cells with MasterPure™ Yeast DNA Purification Kit (Epicentre-Lucigen/Cambio.co.uk MPY80200), by omitting RNase A (included in the kit) from cell lysis step. Total DNA was treated with 0.14 μg μl^−1^ RNase A in 1× TE at 37°C for 30 min (RNase A with no/low salt degrades single-stranded RNA, double-stranded RNA, the RNA moiety of RNA/DNA hybrids and genome-embedded single ribonucleotides; e.g. see (46,49–51)). An aliquot of total DNA (∼2 μg) was heated in presence of alkali (0.3 M KOH) at 55°C for 2 h. Alkali-denatured-total DNA samples were separated, together with the DNA ladder (1 kb plus DNA ladder, Invitrogen 10787018), on an alkaline (50 mM NaOH and 1 mM EDTA, pH 8.0) 1% agarose gel (length 15.5 cm), in alkaline electrophoresis buffer (50 mM NaOH and 1 mM EDTA, pH 8.0), at 1 V cm^−1^, for ∼18–22 h, at room temperature, using Owl separation system model A2 (Thermo Fisher scientific). Note that the buffer was allowed to recirculate using a pump at low flow rate setting (KNF Lab Liquiport 100) to avoid heating during alkaline-gel electrophoresis. The gel was washed in neutralization buffer I (1 M Tris–HCl and 1.5 M NaCl) and then washed briefly in deionized water. The gel was stained for 1 h with 1× SYBR gold (Thermo Fisher scientific S11494) in 0.5× TE, and then washed for 2 × 30 min in 0.5× TE, in a light-protected container, with gentle shaking. SYBR-stained, alkali-fragments from total DNA (henceforth referred to as ‘Afts’) were visualized using Fuji FLA-5100 PhosphorImager. Raw densitometry of SYBR-staining signal was obtained by using AIDA Image Analyzer v.4.15 densitometry software. For the determination of the numbers of total genomic rNMPs see the section ‘Quantitation of genomic ribonucleotides’.

Southern-blotting was performed according to (48), with some modifications. Briefly, the gel from the previous step (with ∼5 μg total DNA in each lane) was washed in alkaline transfer buffer (0.4 N NaOH and 1 M NaCl) for 20 min, and then capillary-transferred onto a nylon Hybond-N+ membrane (GE Healthcare RPN203B), in alkaline transfer buffer, at room temperature, overnight. The membrane was washed in neutralization buffer II (0.5 M Tris–HCl, pH 7.2 and 1 M NaCl), and then DNA was immobilized to the membrane by UV-crosslinking (120 mJ/cm^2^; UV Stratalinker 1800, Stratagene). Strand-specific single-stranded probes were synthesized by PCR using 1 single primer with the *AGP1* double-stranded PCR amplicon as template, in the presence of α-32P-dCTP (for the sequences of primers, see Supplementary Table S2). After 16–24 h hybridization at 65°C, the membrane was washed and exposed to a phosphor imaging screen. Raw densitometry of radioactivity signal was obtained with AIDA Image Analyzer v.4.15 densitometry software.

### Quantitation of genomic ribonucleotides

Ribonucleotide incorporation abundances were estimated using a slightly modified version of the method described previously in (20). Raw densitometric histograms of SYBR-stained Afts were obtained as described in section ‘Detection of genomic ribonucleotides by alkaline-gel electrophoresis combined with Southern hybridization’. After subtracting the background intensity, a smoothing spline with 15 optimally placed internal knots was applied to each lane of the gel by running the SLM tool (D’Errico, 2017*) in Matlab version 9.2. The smoothened intensity curves were resampled at intervals of width ${\rm{\Delta }}d\ = \ 1$mm. For each interval, the characteristic fragment size ($sz$) was calculated following the equation $sz\ = \ {\rm{exp}}(( {d - a)/b} )$, where $d$ represents the electrophoretic distance in the middle point of the interval. Parameters $a$ and $b$ were inferred by fitting the linear model $lm( {d\ \sim\log ( {sz} )} )$ to the peaks of the size reference lane (i.e. 1 Kb plus DNA ladder, Invitrogen 10787018). The fragment count associated with each interval was estimated as ${n_{sz}} = {I_{sz}}\ /sz$, where ${I_{sz}}$ is the smoothened densitometric intensity in that interval. To make the results independent of the total amount of DNA loaded in the lane, the fragment count per size interval per 1Gb of total genomic DNA was obtained as ${n_{sz}}\ ( {per\ 1Gb} ) = {n_{sz}}\ \times {10^9}\ /\ \sum ( {sz\ {n_{sz}}} )$, where the sum extends over all size intervals (Figure 5B). Note that the choice of 1Gb as the unit of measurement is arbitrary and does not change the results by any means. Because the conversion from densitometry intensity to fragment count is highly sensitive to small, noisy fluctuations in the far bottom end of the electrophoretic gel, a cutoff at an electrophoretic distance ${d_{max}}$ was introduced. The value of ${d_{max}}$ was determined under supervision, as the point where fluctuations in the original (non-smoothened) intensities of the loaded lanes became similar in magnitude to those observed in empty lanes, indicating a poor signal-to-noise ratio. Setting a distance cutoff indirectly defined a minimum detectable fragment size equal to$\ s\ {z_{min}} = {\rm{exp}}(( {{d_{max}} - a)/b} )$.

#### Binned distribution of fragment sizes

Binned distributions of fragment sizes were obtained by adding the fragment counts per size interval per 1Gb in bins covering 50 nucleotides (nt) each. The distributions were normalized by dividing the value in each bin by the sum of values in all bins. To account for the fact that $s{z_{min}}$ cutoffs differ across gels, all comparisons between gels were restricted to bins spanning fragment sizes above $s{z_{min}}$. This sub-section is part of section ‘Quantitation of genomic ribonucleotides’ and is related to Supplementary Figure S4.

#### Estimate of numbers of total genomic rNMPs

A preliminary estimate of the number of ribonucleotides per genome, $N$, was obtained by adding, for all intervals, the fragment counts per 1Gb, dividing by ${10^9}$ and multiplying by the size of the yeast haploid genome (∼24 Mb). To account for small fragments that had migrated beyond ${d_{max}}$ (i.e. with sizes below $s{z_{min}}$), the total fragment count was corrected under the assumption that break points are randomly distributed with uniform probability along the genome. Thus, the corrected estimate of the total number of ribonucleotides per genome became ${N_{corr}} = \ N \times ( {2 - {\rm{exp}}\{ { - s{z_{min}} \times \sum {n_{sz}}( {per\ 1Gb} )/{{10}^9}} \}} )$, where the sum extends over all intervals. Note that if the distribution of break points is not uniform along the genome (52), this formula provides a conservative estimate for (i.e. it does not overestimate) the total number of genomic rNMPs. This sub-section is part of section ‘Quantitation of genomic ribonucleotides’ and is related to Figure 5C and Supplementary Table S5.

#### Calculation of the contributions of replicative Pols α, δ and ϵ to synthesis of S. cerevisiae nuclear genome

The percentage of contribution of each replicative Pol was calculated by applying the mathematical formula that we adapted from Reijns *et al.* (53) (see also Supplementary Table S6): ‘(N_ΔPolx_/F_Polx_)/([N_ΔPol α-L868M_/F_Pol α-L868M_] + [N_ΔPol δ-L612M_/F_Pol δ-L612M_] + [N_ΔPol ϵ-M644G_/F_Pol ϵ-M644G_])’ (Reprinted by permission from Copyright Clearance Centre: Springer Nature; Nature; Lagging-strand replication shapes the mutational landscape of the genome; Martin A.M. Reijns *et al.*; 2015). ‘N_ΔPolx_’ represents the subtraction of the number of total genomic rNMPs incorporated *in vivo* by an rNTP-permissive Polx (‘x’ indicates α-L868M, δ-L612M or ϵ-M644G) in a given strain lacking RNase H2, from the number of total genomic rNMPs of the corresponding strain bearing the three WT replicative Pols (α, δ and ϵ) and lacking RNase H2, within the same gel. ‘F_Polx_’ represents the *in vitro* frequency of rNMP incorporation by Polx, i.e. 1 rNMP per 40, 100 and 300 dNMPs for Pols α-L868M, ϵ-M644G, and δ-L612M, respectively (frequencies from (23,54)). This sub-section is part of section ‘Quantitation of genomic ribonucleotides’ and is related to Supplementary Figure S6 and Supplementary Table S6.

### Resolution of formamide-denatured genomic DNA on neutral gel

The protocol was adapted from (20,49), with some modifications. Total DNA was extracted from ∼50 OD_600_ of yeast cells with MasterPure™ Yeast DNA Purification Kit, as described in section ‘Detection of genomic ribonucleotides by alkaline-gel electrophoresis combined with Southern hybridization’. An aliquot of total DNA (∼30 μg) was treated with 0.02 μg μl^−1^ RNase A in 1× TE with high salt (0.5 M NaCl), in a total volume of 175 μl, at 25°C for 1 h (RNase A with high salt degrades selectively single-stranded RNA, while avoiding degradation of double-stranded RNA, the RNA moiety of RNA/DNA hybrids and genome-embedded single ribonucleotides; see e.g. (46,49–51)). DNA was purified with an equal volume of AMPure XP beads (Beckman Coulter A63880). DNA aliquots (∼0.5 μg) were incubated in 1× ThermoPol buffer (New England Biolabs B9004S), either in absence or presence of 25 u of recombinant *E. coli* RNase HII (New England Biolabs M0288S), or in presence of both 25 u RNase HII and 0.1 μg μl^−1^ RNase A, in a total volume of 50 μl, at 37°C for 2 h. As a control for DNA fragmentation, DNA aliquots (∼0.5 μg) were incubated in 1× CutSmart buffer in presence of 2.5 u of Nb.BtsI (New England Biolabs R0707S), either in the absence or presence of 0.1 μg μl^−1^ RNase A, in a total volume of 50 μl, at 37°C for 1 h. DNA was extracted with phenol:chloroform:isoamyl alcohol as described in steps 4-7 in section ‘Reverse transcription of total RNA combined with quantitative PCR (RT-qPCR)’. DNA was re-suspended in 2 μl water and then incubated in presence of 90% formamide and 20 mM EDTA, pH 8, in a total volume of 25 μl, at 37°C for 1 h. Formamide-denatured DNA samples, together with the DNA ladder, were separated by neutral gel-electrophoresis at ∼5.7 V cm^−1^, for 4.5 h, at room temperature, with recirculation of buffer (1% agarose gel in 1× TBE; length of gel 15.5 cm). The gel was subsequently stained with SYBR gold. For other details, see section ‘Detection of genomic ribonucleotides by alkaline-gel electrophoresis combined with Southern hybridization’.

## RESULTS

### Depletion of Rnr1 mildly induces the S-phase checkpoint, greatly reduces dNTP levels and significantly slows cell growth in S-phase

Deletion of the *RNR1* gene is not lethal in the *S. cerevisiae* BY4741/SC288 background. However, *rnr1Δ* mutants are slow growing, relative to the otherwise isogenic wild-type (WT) and suffer from both limited and imbalanced dNTP pools (55,56). Spontaneous suppressor mutations arise in the gene *CRT1*, whose product represses the transcription of the genes *RNR2-4* and *HUG1* during the cell cycle in unperturbed cells ((57,58); see also Supplementary Figure S1). These can reverse the growth defects in *rnr1Δ* strains, likely due to the expansion of dNTP pools (56). To avoid selection for *crt1* suppressors, we constructed the strain *P_GAL_:3HA-RNR1*, in which the *RNR1* gene is under the control of the *P_GAL1/10_* promoter (59,60). We also constructed the strain *P_GAL_:3HA-RNR1 crt1Δ* lacking Crt1. 3HA-Rnr1 expression can be either induced under permissive conditions in galactose-containing medium (plus sucrose to limit Rnr1 over-expression and facilitate yeast growth) or repressed under non-permissive conditions in glucose-containing medium (Figure 1A). We next determined the effects of Rnr1 depletion on the DNA damage and replication checkpoint (henceforth referred to as ‘S-phase checkpoint’; see Supplementary Figure S1), dNTP levels, cell cycle progression and cell growth.

RT-qPCR analyses showed elevated levels of *RNR1* mRNA under permissive conditions (0 h time-point) in both the single mutant *P_GAL_:3HA-RNR1* and double mutant *P_GAL_:3HA-RNR1 crt1Δ*, relative to the WT strain and single mutant *crt1Δ*, which were both cultured in rich YEPD medium (Supplementary Figure S2A, compare lanes e and i with a and d). Following transfer of *P_GAL_:3HA-RNR1* or *P_GAL_:3HA-RNR1 crt1Δ* to glucose medium, *RNR1* transcripts were greatly decreased by 2 h (Supplementary Figure S2A, lanes e-h and i-l). Consistent with this, Western blotting (Figure 1B) showed robust depletion of 3HA-Rnr1 protein following transfer of *P_GAL_:3HA-RNR1* or *P_GAL_:3HA-RNR1 crt1Δ* strains to glucose medium (Figure 1B, 3HA-Rnr1, lanes a–d and e–h).

To induce genotoxic stress, we treated WT cells with hydroxyurea (HU) or methyl-methane sulfonate (MMS). HU inhibits RNR activity by scavenging the tyrosyl free radical in Rnr2, thereby slowing DNA synthesis (see e.g. (60)), while MMS is a DNA alkylating agent that leads to RF arrests (see e.g. (61)). These genotoxic agents led to increased *RNR1* mRNA levels (Supplementary Figure S2A, compare lanes b and c with a), as previously reported for Rnr1 protein (62).

Depletion of Rnr1 in strain *P_GAL_:3HA-RNR1* after 6 h transfer to glucose-containing medium reduced the dNTP pools >3-fold, particularly the levels of dGTP, as compared to the WT strain (Figure 1C, Supplementary Figure S3A and Supplementary Table S4). These results are consistent with previously published data for mutant *rnr1Δ* (56). Limited dNTP pools in the *P_GAL_:3HA-RNR1* strain depleted of Rnr1 are predicted to slow the progression of RFs. RF stalls would in turn trigger the activation of the S-phase checkpoint kinase cascade Mec1-Rad53-Dun1. Activation of this checkpoint can be monitored by western-blotting analysis of Rad53 phosphorylation (phospho-Rad53), visible via reduced electrophoretic mobility (see e.g. (63)). Following depletion of Rnr1 for 6 h, total protein extracts from *P_GAL_:3HA-RNR1* strain showed a noticeable phospho-Rad53 mobility upshift, but this was less marked in the double mutant *P_GAL_:3HA-RNR1 crt1Δ* (Figure 1D, compare lanes a-d with e-h). Phospho-Rad53 was virtually absent in the WT and *crt1Δ* strains but was strongly induced by treatment of the WT strain with HU or MMS (Figure 1D, compare lanes i and l with j and k).

Activation of the S-phase checkpoint kinase cascade Mec1-Rad53-Dun1 under genotoxic stress leads to Dun1-mediated-inhibition of Crt1, thereby leading to the upregulation of the expression of *RNR2-4* and *HUG1* genes ((57,58); see also Supplementary Figure S1). Depletion of Rnr1 in *P_GAL_:3HA-RNR1* strain increased the levels of these mRNAs but, except for *RNR2*, mRNA levels were lower than in *crt1Δ* or *P_GAL_:3HA-RNR1 crt1Δ* at all time-points (Supplementary Figure S2A and B, compare lanes e–h with i–l and d). In WT cells, *RNR3* and *HUG1* mRNAs were virtually absent, and *RNR2* and *RNR4* mRNAs were expressed at low levels; however, *RNR2-4* and *HUG1* mRNAs were all strongly induced by genotoxic stress following treatment with HU or MMS (Supplementary Figure S2A and B, compare lanes b and c with a). There was good concordance between the abundance of *RNR3* mRNA and Rnr3 protein, which was mildly elevated following Rnr1 depletion in *P_GAL_:3HA-RNR1* strain, and strongly elevated in strains lacking Crt1 or the WT strain treated with HU or MMS (Figure 1B, Rnr3, lanes d, e–h, l and j–k; compare with Supplementary Figure S2A, lanes h, i–l, d and b–c, respectively). The phosphorylation of Rad53 and the induced expression of the *RNR3* and *HUG1* genes both indicate that the S-phase checkpoint is modestly activated in *P_GAL_:3HA-RNR1* strain depleted of Rnr1.

Activation of the S-phase checkpoint kinase cascade Mec1-Rad53-Dun1 in unperturbed cells that are replicating their DNA, or in cells that are under genotoxic stress, leads to Dun1-mediated-degradation of Sml1, which is the protein repressor of Rnr1 (see Supplementary Figure S1). Conversely, increased Rnr1-Sml1 association due to over-expression of Rnr1 stabilizes Sml1 (64,65). Western blotting showed that, as expected, Sml1 totally disappeared from WT cells following treatment with HU or MMS (Figure 1B, Sml1, compare lanes j and k with i). In addition, Sml1 was lost upon depletion of Rnr1 in *P_GAL_:3HA-RNR1* strain (Figure 1B, Sml1, lanes a–d), as previously reported for *rnr1Δ* strain (55). Moreover, Sml1 was degraded upon depletion of Rnr1 in *P_GAL_:3HA-RNR1 crt1Δ* strain (Figure 1B, Sml1, lanes e–h). Together, these results suggest that depletion of Rnr1 led to the disappearance of Sml1 in both strains *P_GAL_:3HA-RNR1* and *P_GAL_:3HA-RNR1 crt1Δ*.

Fluorescence-activated cell sorting (FACS) analysis showed that cells from single mutant *P_GAL_:3HA-RNR1* significantly accumulated in S-phase at 6 h depletion of Rnr1, as revealed by the peak between the 1C and 2C positions (lower-part of Figure 1E), which is consistent with a previous report that analyzed *rnr1Δ* cells (55). Loss of Crt1 in the *P_GAL_:3HA-RNR1 crt1Δ* double mutant strain, however, substantially reduced cell accumulation in S-phase after depletion of Rnr1 for 6 h (upper-part of Figure 1E).

Finally, in drop test growth assays, *P_GAL_:3HA-RNR1* strain showed WT growth in galactose plus sucrose medium, but grew slower than the WT strain in glucose medium (Figure 1F, compare rows e with a). In contrast, growth of *P_GAL_:3HA-RNR1 crt1Δ* strain was similar to the WT strain and the single mutant *crt1Δ* in glucose medium (Figure 1F, compare rows f with a and b). Loss of Sml1 did not improve the growth of the double mutant *P_GAL_:3HA-RNR1 sml1Δ* relative to the single mutant *P_GAL_:3HA-RNR1* in glucose medium, which is in accordance with our western blotting results showing that Sml1 protein is degraded in the single mutant *P_GAL_:3HA-RNR1* upon depletion of Rnr1 (compare Figure 1F, rows e and g with Figure 1B, Sml1, lanes a-d). The double mutant *P_GAL_:3HA-RNR1 dun1Δ* was non-viable on glucose medium (Figure 1F, row h), suggesting that induced expression of Rnr3 via the activated S-phase checkpoint kinase cascade Mec1-Rad53-Dun1 (see Supplementary Figure S1) is essential for the survival of single mutant *P_GAL_:3HA-RNR1* depleted of Rnr1. This result is consistent with a previous report showing that Dun1 is essential for the viability of *rnr1* hypomorphic mutants with limited dNTP pools (66).

Collectively, these results indicate that depletion of Rnr1 mildly activates the S-phase checkpoint, greatly reduces and imbalances dNTP levels, and significantly slows S-phase progression and cell growth. Constitutive replicative stress in Rnr1-depleted strains is likely to reflect a combination of limited and imbalanced dNTP pools, as previously reported for *rnr1* hypomorphic mutants (66,67). The additional loss of Crt1 in Rnr1-depleted cells should expand and balance dNTP pools, as previously reported for the double mutant *rnr1Δ crt1Δ* (56), which would mitigate replicative stress and restore cell growth.

### Triple mutants depleted of Rnr1 and lacking RNases H1 and H2 are non-viable, but cell growth is restored by the presence of Rnh201-RED

We hypothesized that reduced dNTP pools in cells depleted of Rnr1 would increase the load of genome-embedded single rNMPs in mutants lacking the RNase H2-dependent-RER pathway, thereby compromising genome stability and cell growth. We further hypothesized that accumulation of persistent RNA/DNA hybrids (e.g. R-loops) in absence of RNases H1 and H2 in cells depleted of Rnr1 would aggravate replicative stress and compromise genomic integrity and cell viability. In principal, RER activity, hybrid-removal activity, or both RNase H activities might be important for growth of cells depleted of Rnr1. To assess this, we deleted one of the genes encoding for the heterotrimeric enzymatic complex RNase H2 (yeast RNase H2 is formed of the catalytic subunit Rnh201 and the accessory subunits Rnh202 and Rnh203 (68)), and/or the gene encoding for the monomeric enzyme RNase H1 in strains carrying *P_GAL_:3HA-RNR1*. We then performed drop test growth assays to determine viability.

**Figure 2.**
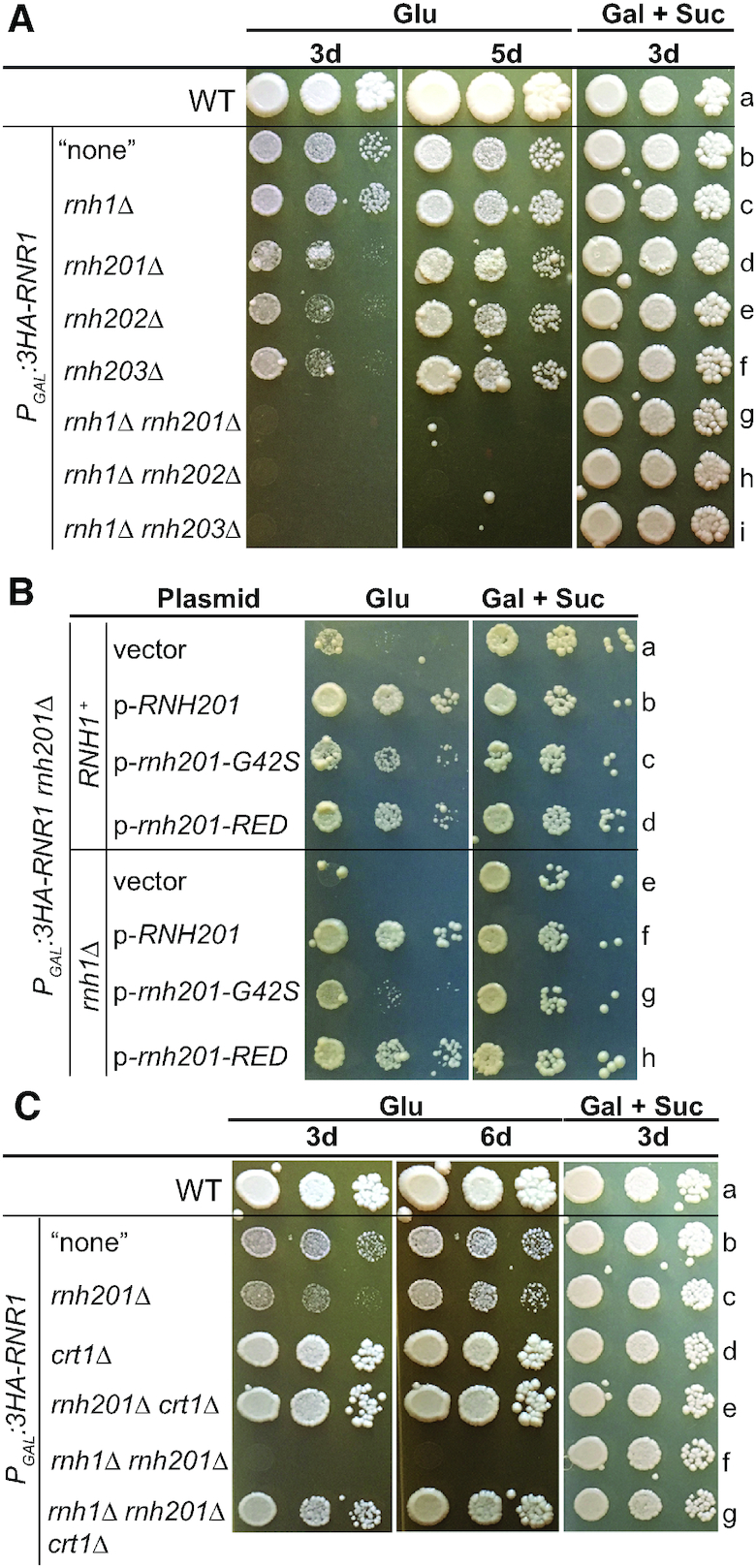
The lethality of Rnr1-depleted triple mutants lacking RNases H1 and H2 is suppressed by the variant Rnh201-RED. (**A**) Depletion of Rnr1 in mutants lacking RNase H1, RNase H2 or both enzymes has different effects on their growth. Drop test growth assays of strain WT, and strains carrying *P_GAL_:3HA-RNR1* without gene deletion (labeled “none"), or with deletion of the gene *RNH1*, *RNH201*, *RNH202*, or *RNH203*, or both genes *RNH1* and *RNH201*, or *RNH1* and *RNH202*, or *RNH1 and RNH203*. Cells were grown in YPGS (2% galactose and 1% sucrose) liquid medium overnight at 30°C. Serial dilutions were plated on YEPD (2% glucose) and YPGS solid media. Plates were incubated at 30°C. Photographs were taken at the indicated number of days (d). ‘Glu’ stands for glucose. ‘Gal + Suc’ stands for galactose plus sucrose. The horizontal line across the images is included for clarity. See Supplementary Table S1 for the list of strains. For the ease of comparison, a unique Latin alphabet letter is allocated for each row. One representative experiment is shown of at least three independent ones. (**B**) The variant Rnh201-G42S suppresses less well the growth defects of Rnr1-depleted triple mutants lacking RNases H1 and H2 than the variant Rnh201-RED. Drop test growth assays of strains *P_GAL_:3HA-RNR1 rnh201Δ* and *P_GAL_:3HA-RNR1 rnh201Δ rnh1Δ* that have an empty vector, or a plasmid expressing WT Rnh201, variant Rnh201-G42S, or variant Rnh201-RED. Cells were grown overnight in liquid minimal medium lacking leucine with 2% galactose and 1% sucrose at 30°C. Serial dilutions were plated on solid minimal medium lacking leucine with either 2% glucose, or 2% galactose and 1% sucrose. Photographs were taken after 7 days of incubation at 30°C. For other details, see (A). (**C**) Cells depleted of Rnr1 and lacking RNases H1 and H2 grow like the WT strain in absence of Crt1. Drop test growth assays of strain WT, and strains carrying *P_GAL_:3HA-RNR1* without gene deletion (labeled “none"), or with deletion of the gene *RNH201* or *CRT1*, or both genes *RNH201* and *CRT1*, or *RNH1* and *RNH201*, or the three genes *RNH1*, *RNH201* and *CRT1*. For other details, see (A).

We found that on glucose medium (Figure 2A): (i) The three double mutants carrying *P_GAL_:3HA-RNR1* together with *rnh201Δ, rnh202Δ* or *rnh203Δ* grew slower than the single mutant *P_GAL_:3HA-RNR1* (compare rows d–f with b). (ii) The double mutant *P_GAL_:3HA-RNR1 rnh1*Δ grew similarly to the single mutant *P_GAL_:3HA-RNR1* (compare rows c with b). (iii) The three triple mutants carrying *P_GAL_:3HA-RNR1 rnh1Δ* together with *rnh201Δ, rnh202*Δ or *rnh203*Δ did not grow at all (rows g–i). This led us to infer that loss of RNases H1 and H2 in cells depleted of Rnr1 induced synthetic lethality. Finally, strains carrying *P_GAL_:3HA-RNR1* without RNase H1, or RNase H2, or both enzymes grew similarly to the WT and single mutant *P_GAL_:3HA-RNR1* in galactose plus sucrose medium, showing that the lack of one or both of these enzymes does not clearly affect cell growth in the presence of Rnr1, which is consistent with previous reports (see e.g. (27,69)) (Figure 2A, compare lanes c–i with a and b).

To determine which of the two RNase H2 activities is important for preventing the growth defects observed in strains depleted of Rnr1 and lacking RNase H2 in presence/absence of RNase H1, we made use of two mutant variants of RNase H2: (i) Rnh201-P45D-Y219A which has no RER activity, but retains ∼50% of its hybrid-removal activity on long RNA/DNA hybrids (70) (henceforth designated as ‘Rnh201-RED’; RED stands for Ribonucleotide Excision Defective). (ii) Rnh201-G42S, which has ∼2% and <10%, RER and hybrid-removal activities, respectively (70). Note that *S. cerevisiae* Rnh201-G42S is homologous to the human mutant RNase H2^G37S^, which is associated with Aicardi-Goutières Syndrome (AGS)—a rare neuro-inflammatory autoimmune disorder in humans (71).

We transformed the double mutant *P_GAL_:3HA-RNR1 rnh201Δ* and triple mutant *P_GAL_:3HA-RNR1 rnh201Δ rnh1Δ* with empty vector, p-*RNH201*, p-*rnh201-G42S* or p-*rnh201-RED*. We found that on glucose medium, the variant Rnh201-RED suppressed the growth defects in both of these strains slightly less well than the WT Rnh201 (Figure 2B, compare rows a with b and d, and e with f and h). Suppression of the growth defects in glucose medium by the variant Rnh201-G42S was similar to Rnh201-RED in the *P_GAL_:3HA-RNR1 rnh201Δ* strain, but was much weaker in the *P_GAL_:3HA-RNR1 rnh1Δ rnh201Δ* strain (Figure 2B, compare rows c and d with g and h). These results indicate that both single genomic rNMPs and RNA/DNA hybrids are detrimental for the growth of strains depleted of Rnr1 and lacking RNase H2 or RNases H1 and H2. Because the variant Rnh201-RED, which has much higher hybrid-removal activity than the variant Rnh201-G42S, better alleviated the growth defects of the triple mutant depleted of Rnr1 and lacking RNases H1 and H2, we concluded that removal of RNA/DNA hybrids is the critical factor for survival of this triple mutant. Note that it is possible that, because RER is absent (i.e. in presence of plasmid vector) or defective (i.e. in presence of plasmid expressing Rnh201-G42S), persistent RNA/DNA hybrids, e.g. R-loops, could increase and/or become highly toxic in cells depleted for Rnr1 and lacking RNases H1 and H2.

Finally, we tested the effects of the loss of Crt1, which leads to the expansion of dNTP pools (56), on the growth of strains *P_GAL_:3HA-RNR1 rnh201Δ* and *P_GAL_:3HA-RNR1 rnh1Δ rnh201Δ* in glucose medium. Drop test growth assays showed that the absence of Crt1 fully suppressed the growth defects in both strains in glucose medium, relative to the WT strain (Figure 2C, compare rows c with e, and f with g, and e and g with a). Increased dNTP synthesis in absence of Crt1 should improve DNA synthesis (both replication and repair) (14,60,72) and reduce utilization of rNTPs by WT replicative Pols (10,13), thereby mitigating genomic instability defects that can be induced by unrepaired single genomic rNMPs and persistent RNA/DNA hybrids.

### Deletions of 2–5 bp are greatly increased in RER-deficient Rnr1-depleted double mutants

Earlier reports in budding yeast indicated that Top1-mediated incisions at unrepaired single genomic rNMPs can cause replicative stress, genomic instability, and a *Δ*2–5 bp mutation signature within short tandem repeats, which is predominately associated with the leading strand (see e.g. (22,34,37,54,73–77); for reviews, see e.g. (12,31)). Drop test growth assays in Figure 2 suggested that accumulation of unrepaired single genomic rNMPs is greatly increased in *P_GAL_:3HA-RNR1 rnh201Δ* strain depleted of Rnr1. We therefore determined whether Top1-mediated *Δ*2–5 bp mutations are increased in *P_GAL_:3HA-RNR1 rnh201Δ* strain depleted of Rnr1, relative to the *rnh201Δ* strain.

**Figure 3.**
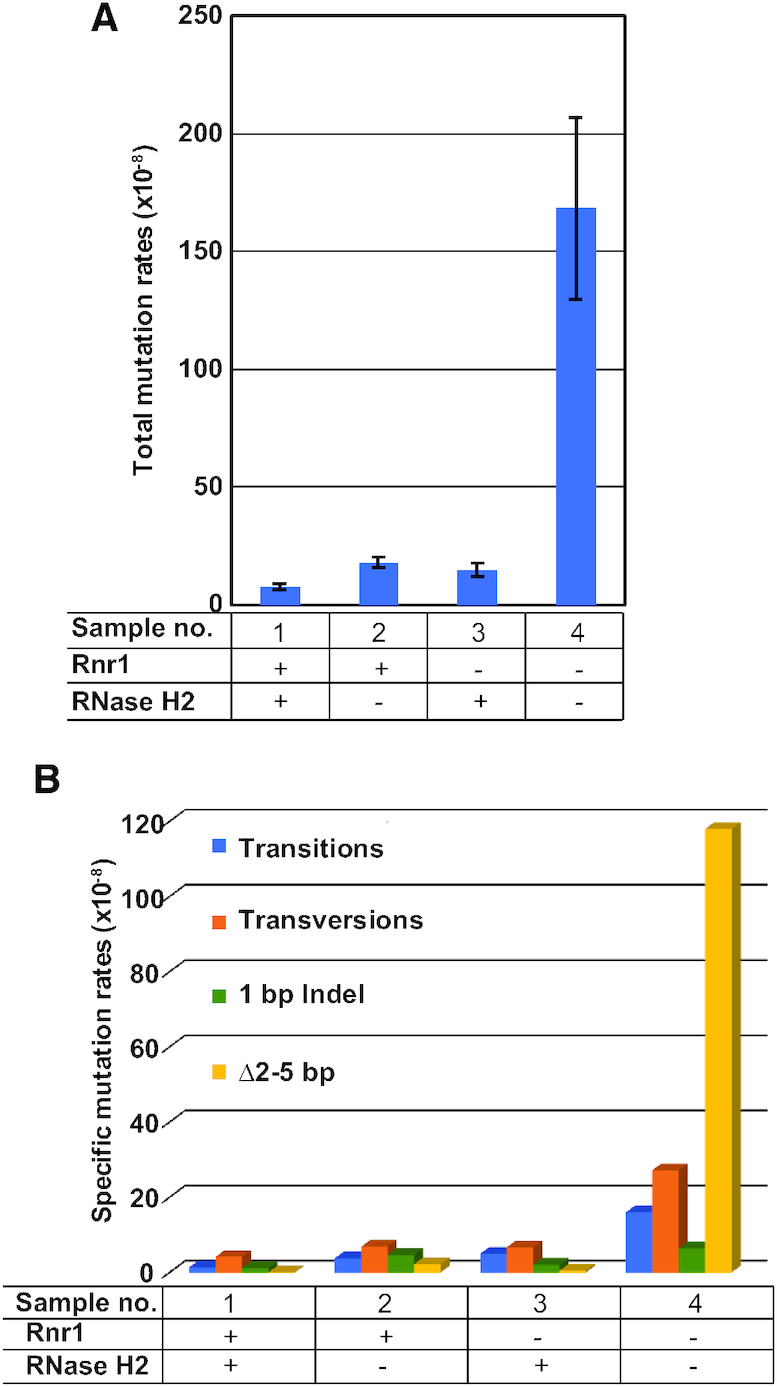
Total and Δ2–5 bp mutation rates of *CAN1* are highly increased in RER-deficient Rnr1-depleted double mutant. (**A**) Total mutation rate is highly increased in cells lacking RNase H2 and depleted of Rnr1. WT strain (sample 1), single mutant *rnh201Δ* (sample 2), single mutant *P_GAL_:3HA-RNR1* (sample 3), and double mutant *P_GAL_:3HA-RNR1 rnh201Δ* (sample 4), were grown in rich YEPD (2% glucose) solid medium. In these growth conditions, Rnr1 should be expressed at WT levels in samples 1 and 2, and Rnr1 should be depleted in samples 3 and 4. Total mutation rates are plotted on the Y-axis. The graph represents the average and S.E.M. of 4 independent experiments. See also Supplementary Table S3. Symbols on the organigram below the plot: + and – indicate that the protein is present or absent, respectively. (**B**) Δ2–5 bp specific mutation rate is highly increased in cells lacking RNase H2 and depleted of Rnr1. Specific mutation rates of *CAN1* (mutation-spectra) for the same strains and growth conditions as in (A). Specific mutation rates are plotted on the Y-axis. The different types of mutations are color-coded. ‘1 bp Indel’ stands for 1 base pair insertion/deletion. ‘Δ2–5 bp’ stands for 2–5 base pairs deletion. See also Supplementary Table S3. For other details see (A).

We analyzed total mutation rates and specific mutation rates (i.e. transitions, transversions, 1 bp indel and *Δ*2–5 bp) for the *CAN1* gene in the WT, *rnh201Δ*, *P_GAL_:3HA-RNR1* and *P_GAL_:3HA-RNR1 rnh201Δ* strains, grown in glucose medium (Figure 3; see also Supplementary Table S3). We found that total mutation rates and *Δ*2–5 bp rates in single mutant *rnh201Δ* were 2.4-fold and 19.3-fold higher than in the WT strain, respectively (Figure 3A and B; compare samples 2 with 1). These data are in agreement with earlier reports using other yeast backgrounds (see e.g. (23,34,73,76)). Additionally, the single mutant *P_GAL_:3HA-RNR1* depleted of Rnr1 showed a modest 2-fold increase in total mutation rates, and slightly higher specific mutation rates, relative to the WT strain (Figure 3A and B, compare samples 3 with 1). Strikingly, the double mutant *P_GAL_:3HA-RNR1 rnh201Δ* depleted of Rnr1 showed 23-fold and 1039.3-fold increase in total mutation rates and *Δ*2–5 bp rates, respectively, relative to the WT strain (Figure 3A and B, compare samples 4 with 1). These results are consistent with published mutation rates (using *CAN1* and other reporters) for the double mutant *pol2-M644G rnh201Δ*, which accumulates high loads of single rNMPs in the leading strand (see e.g. (23,54,77)). This leads us to infer that Top1-mediated RNA–DNA damage is greatly increased in the double mutant *P_GAL_:3HA-RNR1 rnh201Δ* depleted of Rnr1.

### Loss of Top1 reverses the severe growth defects of Rnr1-depleted RER-deficient Pol ϵ-M644G or δ-L612M triple mutants

**Figure 4.**
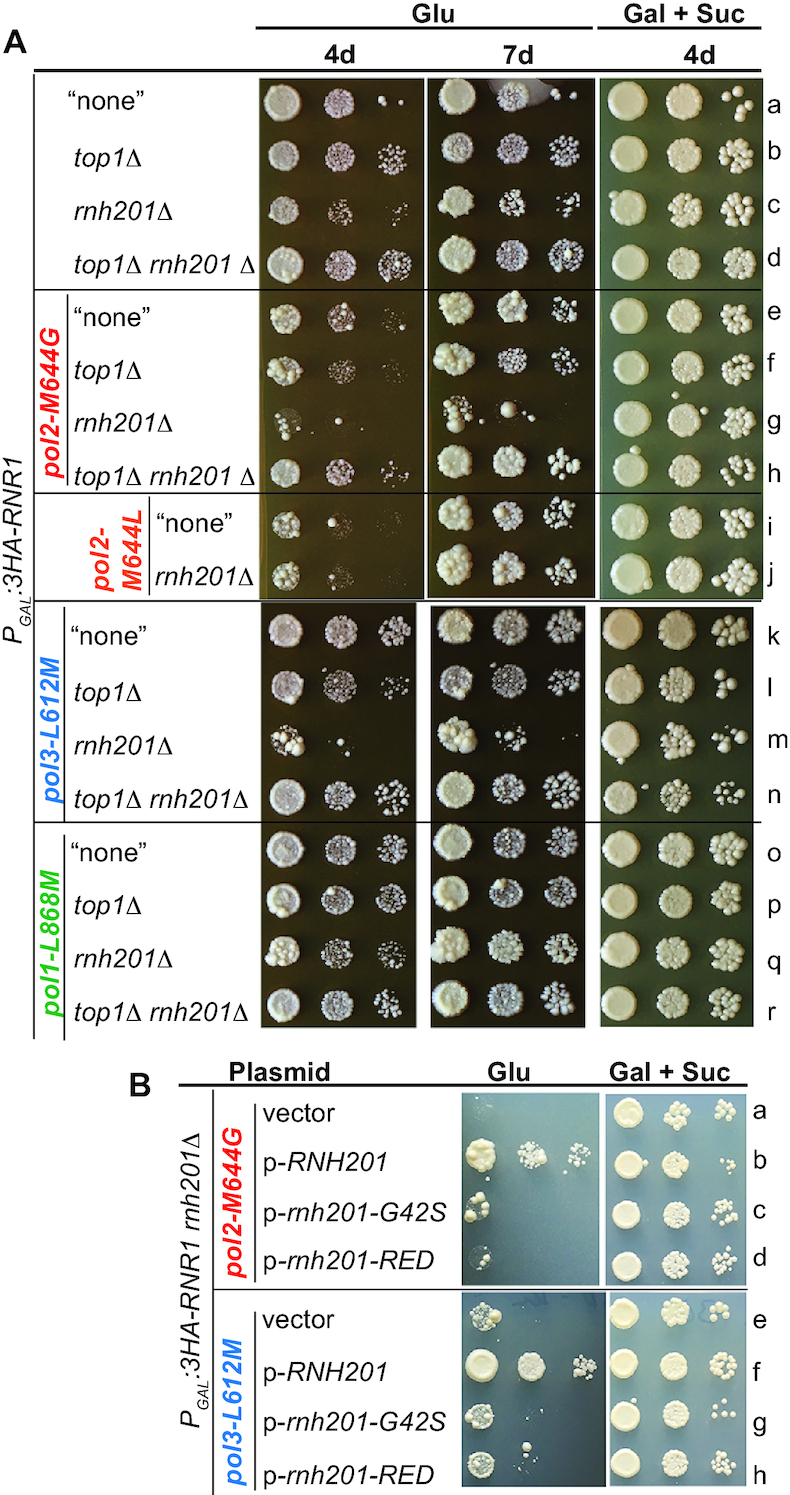
RER-deficient Rnr1-depleted triple mutants bearing Pol ϵ-M644G or δ-L612M, but not α-L868M, show Top1-dependent severe growth defects. (**A**) RER-deficient mutants depleted of Rnr1 and bearing Pol ϵ-M644G or δ-L612M show severe growth defects, but these defects are suppressed in absence of Top1. Drop test growth assays of strains carrying *P_GAL_:3HA-RNR1* without gene deletion (labeled “none"), or with deletion of the gene *TOP1* or *RNH201*, or both genes *TOP1* and *RNH201*, and strains carrying both *P_GAL_:3HA-RNR1* and allele *pol2-M644G*, *pol3-L612M*, or *pol1-L868M*, without gene deletion (labeled “none"), or with deletion of the gene *TOP1* or *RNH201*, or both genes *TOP1* and *RNH201*, and strains carrying both *P_GAL_:3HA-RNR1* and allele *pol2-M644L*, without gene deletion (labeled “none"), or with deletion of the gene *RNH201*. Cells were grown in YPGS (2% galactose and 1% sucrose) liquid medium overnight at 30°C. Serial dilutions were plated on YEPD (2% glucose) and YPGS solid media. Plates were incubated at 30°C. Photographs were taken at the indicated number of days (d). The images for each incubation time are from the same plate. The horizontal lines across the images are included for clarity. ‘Glu’ stands for glucose. ‘Gal + Suc’ stands for galactose plus sucrose. For the ease of comparison, a unique Latin alphabet letter is allocated for each row. See Supplementary Table S1 for the list of strains. One representative experiment is shown of at least three independent ones. (**B**) The variant Rnh201-RED does not suppress the severe growth defects of RER-deficient mutants depleted of Rnr1 and bearing Pol ϵ-M644G or δ-L612M. Drop test growth assays of strains *P_GAL_:3HA-RNR1 pol2-M644G rnh201Δ* and *P_GAL_:3HA-RNR1 pol3-L612M rnh201Δ* that have the same plasmids as in Figure 2B. Cells were grown overnight in liquid minimal medium lacking leucine with 2% galactose and 1% sucrose at 30°C. Serial-dilutions were plated on solid minimal medium lacking leucine with 2% glucose, or with 2% galactose and 1% sucrose. Photographs were taken after incubation for 7 days at 30°C. The images for each growth condition are from the same plate. The horizontal line across the images is included for clarity. For other details, see (A).

The data in Figures 2 and 3 suggested that the accumulation of single genomic rNMPs and the associated Top1-mediated DNA damage are greatly increased in the double-mutant *P_GAL_:3HA-RNR1 rnh201Δ* depleted of Rnr1. Accordingly, drop test growth assays showed that the triple mutant *P_GAL_:3HA-RNR1 rnh201Δ top1Δ* grew better than the double mutant *P_GAL_:3HA-RNR1 rnh201Δ* in glucose medium, whereas both mutants grew similarly to each other in galactose plus sucrose medium (Figure 4A, compare rows a–d).

To modulate the levels of rNMP incorporation in genomic DNA in cells depleted of Rnr1 and lacking RER, we employed three rNTP-permissive Pols harboring alleles *pol1-L868M*, *pol2-M644G* or *pol3-L612*M (henceforth designated as ‘Pol α-L868M’, ‘Pol ϵ-M644G’ and ‘Pol δ-L612M’, respectively). We also employed one steric gate Pol ϵ variant harboring allele *pol2-M644L* (henceforth designated as ‘Pol ϵ-M644L’) that has higher selectivity against utilization of rNTPs as compared to its WT parent enzyme. The three Pol mutators α-L868M, ϵ-M644G and δ-L612M, which have both reduced base and sugar selectivity, have been instrumental in determining which Pol is primarily responsible for the synthesis of leading stand (Pol ϵ) and lagging strand (Pols α and δ) (for a review, see e.g. (78)), and in unraveling the roles of RNase H2-dependent RER as well (see e.g. (5,22,23,34,35,52–54,73–75,77,79–83)), in *S. cerevisiae*. We separately introduced the four Pol mutant alleles into BY4741 and determined their effects on dNTP levels, S-phase checkpoint activation and cell growth, in combination with the expression or depletion of Rnr1, in the presence or absence of RNase H2 and/or Top1.

Previously published data indicate that survival of yeast mutants harboring Pol ϵ-M644G requires the expansion of dNTP pools, by constitutive activation of the S-phase checkpoint (23,84). Deletion of *RNH201* in these mutants further increases the dNTP levels, which is indicative of further exacerbation of replicative stress (23). However, Williams *et al.* (54) found that the presence of Pol α-L868M or δ-L612M in yeast cells does not lead to increased dNTP abundance, either in the presence or absence of RNase H2. Here, we found that, as described previously (23,54), the *pol2-M644G rnh201Δ* double mutant has ∼4-fold higher dNTP pool levels than the single mutant *rnh201Δ* and the two double mutants *pol1-L868M rnh201Δ* and *pol3-L612M rnh201Δ*. The latter three strains showed only slightly increased dNTP concentrations as compared to the WT strain (Supplementary Figure S3A and Supplementary Table S4, compare samples 1–5). Interestingly, depletion of Rnr1 for 6 h decreased dNTP pools > 3-fold in strains carrying *P_GAL_-3HA:RNR1*, regardless of the status of RER, as compared to the WT strain (Supplementary Figure S3A and Supplementary Table S4, compare samples 6 and 7 with 1). Moreover, the >3-fold decrease in dNTP levels in Rnr1-depleted RER-deficient strains remained even in combination with Pol ϵ-M644G, δ-L612M or α-L868M (Supplementary Figure S3A and Supplementary Table S4, compare samples 7–10). These observations are in accordance with western blotting data for the activation of the S-phase checkpoint in Supplementary Figure S3B. Induction of Rnr3 expression and phosphorylation of Rad53 were modest in strains carrying *P_GAL_-3HA:RNR1* and depleted of Rnr1 for 6 h, with or without active RER, or both lacking RER and harboring an rNTP-permissive Pol (lanes b, f, h, j and l, Rnr3 and P-Rad53). We conclude that the presence of an rNTP-permissive Pol (ϵ-M644G, δ-L612M or α-L868M) does not affect dNTP concentrations in cells lacking RNase H2 and depleted of Rnr1.

Drop test growth assays with strains harboring Pol ϵ-M644G or ϵ-M644L showed that, in glucose medium: (i) The two double mutants *P_GAL_:3HA-RNR1 pol2-M644G* and *P_GAL_:3HA-RNR1 pol2-M644L* grew slower than the single mutant *P_GAL_:3HA-RNR1*; however, growth of all three strains was comparable after prolonged growth on glucose (Figure 4A, compare rows e and i with a; see also Supplementary Figure S3C, compare rows d with b). Published *in vitro* data (23) indicate that purified Pols ϵ-M644G and ϵ-M644L have lower catalytic efficiency than their WT parent enzyme. We therefore hypothesize that these Pol variants are less active following Rnr1 depletion, due to limited and/or unbalanced dNTP concentrations, than with WT levels of Rnr1. (ii) The growth of the triple mutant *P_GAL_:3HA-RNR1 pol2-M644G rnh201Δ* was severely affected with respect to *P_GAL_:3HA-RNR1 pol2-M644G* (Figure 4A, compare rows g with e; see also Supplementary Figure S3C, compare rows e with d). (iii) The additional deletion of *TOP1* in *P_GAL_:3HA-RNR1 pol2-M644G rnh201Δ* reversed its severe growth defects (Figure 4A, compare rows h with g). (iv) The triple mutant *P_GAL_:3HA-RNR1 pol2-M644L rnh201Δ* grew as slowly as *P_GAL_:3HA-RNR1 pol2-M644L* (Figure 4A, compare rows j with i). Together, these observations suggest that excessive incorporation of rNMPs by Pol ϵ-M644G in leading strand of RER-deficient cells depleted of Rnr1 leads to deleterious Top1-mediated RNA-DNA damage.

Drop test growth assays with mutants harboring Pol δ-L612M or α-L868M (Figure 4A) showed that, in glucose medium: (i) The two double mutants *P_GAL_:3HA-RNR1 pol3-L612M* and *P_GAL_:3HA-RNR1 pol1-L868M* grew similarly to the single mutant *P_GAL_:3HA-RNR1* (compare rows k and o with a). (ii) The triple mutant *P_GAL_:3HA-RNR1 pol1-L868M* *rnh201Δ* grew only slightly slower than *P_GAL_:3HA-RNR1 pol1-L868M* (compare rows q with o). (iii) The triple mutant *P_GAL_:3HA-RNR1 pol3-L612M rnh201Δ* showed severe, synergistic growth impairment, as compared to *P_GAL_:3HA-RNR1 pol3-L612M* (compare rows m with k). Note that growth impairment in *P_GAL_:3HA-RNR1 pol3-L612M rnh201Δ* was slightly less marked than in *P_GAL_:3HA-RNR1 pol2-M644G rnh201Δ* (compare rows m with g). (iv) Loss of Top1 in the triple-mutant *P_GAL_:3HA-RNR1 pol3-L612M rnh201Δ* rescued the severe co-synthetic growth defect (compare rows n with m). (v) The quadruple mutant *P_GAL_:3HA-RNR1 pol1-L868M rnh201Δ top1Δ* grew better than *P_GAL_:3HA-RNR1 pol1-L868M rnh201Δ TOP1^+^* (compare rows r with q). Together, these results suggest that following depletion of Rnr1 the excessive incorporation of rNMPs by Pol δ-L612M in lagging strand leads to detrimental Top1-mediated RNA–DNA damage in RER-deficient cells. However, rNMP incorporation by Pol α-L868M in lagging strand is much less damaging.

Top1-mediated cleavage occurs at RNA-DNA junctions of unrepaired embedded single or stretches of rNMPs. Both can be incorporated by rNTP-permissive Pols in triple mutants *P_GAL_:3HA-RNR1 rnh201Δ pol2-M644G* and *P_GAL_:3HA-RNR1 rnh201Δ pol3-L612M* in glucose medium, thereby compromising genome stability and cell viability. To determine whether single rNMPs alone or a combination of both single and stretches of rNMPs contributed to Top1-mediated RNA-DNA damage, we transformed these triple mutants with empty vector, p-*RNH201*, p-*rnh201-G42S* or p-*rnh201-RED*. Drop test growth assays showed that, in glucose medium, in sharp contrast to WT Rnh201, neither the variant Rnh201-RED nor the variant Rnh201-G42S suppressed the severe growth defects of the triple mutants (Figure 4B, compare rows a–d and e–h). Because Rnh201-RED processes RNA/DNA hybrids but cannot process single rNMPs (70), we concluded that unrepaired single genomic rNMPs are likely to constitute the major source of Top1-induced DNA damage and growth defects in the triple-mutants in glucose medium. Supporting this conclusion, endogenous RNase H1 failed to support the growth of these triple mutants in glucose medium, despite processing RNA/DNA hybrids (Figure 4A rows g and m and 4B rows a, c–d, e and g–h). Additionally, we found that, in sharp contrast to *P_GAL_:3HA-RNR1 pol2-M644G rnh201Δ*, the triple mutant *P_GAL_:3HA-RNR1 pol2-M644G rnh1Δ* grew similarly to double mutant *P_GAL_:3HA-RNR1 pol2-M644G* in glucose medium (Supplementary Figure S3C, compare rows d-f). Finally, one cannot exclude the possibility that, in addition to single rNMPs, very short stretches of rNMPs that are not substrates for RNase H1 and are not cleaved efficiently by Rnh201-RED (e.g. 2–3 consecutive rNMPs; see (70)), might also contribute to Top1-mediated RNA-DNA damage in the triple mutants *P_GAL_:3HA-RNR1 rnh201Δ pol2-M644G* and *P_GAL_:3HA-RNR1 rnh201Δ pol3-L612M* in glucose medium.

Finally, drop test growth assays showed that growth of the three triple mutants *P_GAL_:3HA-RNR1 pol2-M644Gcrt1Δ*, *P_GAL_:3HA-RNR1 pol3-L612M crt1Δ* and *P_GAL_:3HA-RNR1 pol1-L868M crt1Δ*, and the three quadruple mutants *P_GAL_:3HA-RNR1 pol2-M644G crt1Δrnh201Δ*, *P_GAL_:3HA-RNR1 pol3-L612M crt1Δ rnh201Δ*, and *P_GAL_:3HA-RNR1 pol1-L868M crt1Δ rnh201Δ* was similar to both the double mutant *P_GAL_:3HA-RNR1 crt1Δ* and the isogenic WT strain, in glucose medium (Supplementary Figure S3D, compare lanes e, g, i, k, m, o with a and c). We speculate that the expansion of dNTP pools due to the loss of Crt1 in the two quadruple mutants *P_GAL_:3HA-RNR1 pol2-M644G crt1Δ rnh201Δ* and *P_GAL_:3HA-RNR1 pol3-L612M crt1Δ rnh201Δ* depleted of Rnr1 improved DNA synthesis (both replication and repair) and concomitantly reduced utilization of rNTPs by the rNTP-permissive Pol mutants. This presumably mitigated the deleterious impact of Top1-mediated RNA-DNA damage on genome stability and cell growth.

### Measurements of the numbers of genomic rNMPs by alkali-fragmentation of total DNA

**Figure 5.**
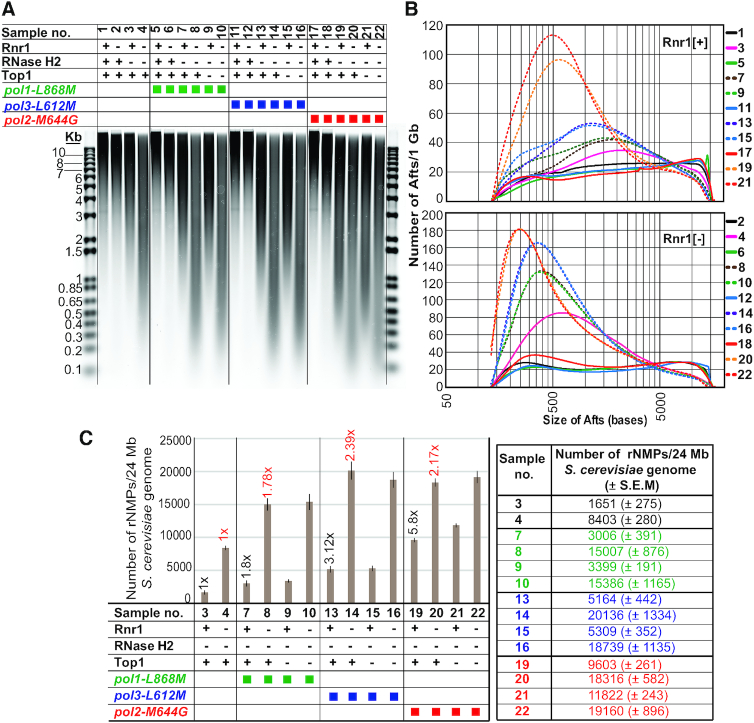
Determination of the numbers of genomic rNMPs by alkali-fragmentation of ribonucleotide-containing total DNA. (**A**) Resolution by gel electrophoresis of alkali-fragments from total DNA (Afts). The following strains (see Supplementary Table S1 for the list of strains) are represented by symbols on the organigram. Rnr1 [+] condition (Rnr1 indicated by ‘+’ on organigram): 1. WT. 3. *rnh201*Δ. 5. *pol1-L868M*. 7. *pol1-L868M rnh201*Δ. 9. *pol1-L868M rnh201*Δ*top1*Δ. 11. *pol3-L621M*. 13. *pol3-L621M rnh201*Δ. 15. *pol3-L621M rnh201*Δ*top1*Δ. 17. *pol2-M644G*. 19. *pol2-M644G rnh201*Δ. 21. *pol2-M644G rnh201*Δ*top1*Δ. Rnr1 [−] condition (Rnr1 indicated by ‘−’ on organigram): 2. *P_GAL_:3HA-RNR1*. 4. *P_GAL_:3HA-RNR1 rnh201*Δ. 6. *P_GAL_:3HA-RNR1 pol1-L868M*. 8. *P_GAL_:3HA-RNR1 pol1-L868M rnh201*Δ. 10. *P_GAL_:3HA-RNR1 pol1-L868M rnh201*Δ *top1*Δ. 12. *P_GAL_:3HA-RNR1 pol3-L621M*. 14. *P_GAL_:3HA-RNR1 pol3-L621M rnh201*Δ. 16. *P_GAL_:3HA-RNR1 pol3-L621M rnh201*Δ*top1*Δ. 18. *P_GAL_:3HA-RNR1 pol2-M644G*. 20. *P_GAL_:3HA-RNR1 pol2-M644G rnh201*Δ. 22. *P_GAL_:3HA-RNR1 pol2-M644G rnh201*Δ *top1*Δ. For Rnr1 [+] condition (Rnr1 expression at WT levels), strains carrying the gene *RNR1* under the control of its native promoter were cultured in rich YEPD (2% glucose) medium at 30°C and harvested in exponential phase at OD_600_ ∼0.5–0.6. For Rnr1 [−] condition (Rnr1 depletion), strains carrying *P_GAL_:3HA-RNR1* were cultured at 30°C in liquid minimal medium lacking histidine with 2% galactose and 2% sucrose. Cells at OD_600_ ∼0.2 were transferred to liquid minimal medium lacking histidine with 2% glucose, maintained in exponential phase, and harvested 6 h after transfer to glucose-containing medium (see also Material and Methods). Alkali-treated DNA samples (∼2 μg per lane) together with the DNA ladder (in duplicate) were resolved on an alkaline 1% agarose gel. The gel was neutralized and stained with SYBR gold (see also Materials and Methods). Selected molecular weights of the left-hand DNA ladder are labeled in kb. The vertical lines along the image of the gel are included for clarity. Symbols on the organigram: + and – indicate that the protein is present or absent, respectively; green, blue and red squares depict the alleles *pol1-L868M*, *pol3-L612M* and *pol2-M644G*, respectively. (**B**) Distribution of Afts sizes. Densitometry of SYBR-stain signal from samples in (A) was measured at 0.01 mm intervals and the values were normalized to the length of the DNA fragment (for mathematical modeling of the data see the section ‘Quantitation of genomic ribonucleotides’ in Materials and Methods). The number of Afts of a given size per 1Gb of total DNA is plotted on the Y-axis. Sizes of Afts in bases are plotted on the X-axis. 50, 500 and 5000 bases are indicated on the X-axis of the lower-plot. Units on the Y-axis (per 1 Gb) were chosen arbitrarily. Because the yeast haploid genome size is 24 Mb, the expected number of Afts of a given size in a single yeast genome is approximately 1/42 of the numbers shown on the Y-axis. Note also the logarithmic scale of the X-axis. For the ease of comparison, histograms for conditions Rnr1 [+] and Rnr1 [−] are represented separately in the upper- and lower-plots, respectively. See also Supplementary Figure S4 for averaged data from 4 independent repeats with S.E.M. (**C**) Numbers of total genomic rNMPs. Total genomic rNMPs present in the 24 Mb haploid yeast genome of selected samples from (A) are plotted on the Y-axis. Shown are averaged data for four independent repeats with S.E.M. For the ease of comparison, averaged data of total genomic rNMPs are also indicated on the small table to the right-hand of the bar plot. The fold differences of samples 7, 13 and 19 relative to sample 3 are indicated above the bars on the plot by black numbers, and the fold differences of samples 8, 14 and 20 relative to sample 4 are indicated above the bars on the plot by red numbers (for *P*-values, see Supplementary Table S5). Symbols on the organigram (below the bar plot) are as described in (A). For the calculation of the total numbers of DNA breaks by mathematical modeling, see section ‘Quantitation of genomic ribonucleotides’ in Material and Methods (numbers of DNA breaks from four independent experiments are represented in Supplementary Table S5). For the calculations of total numbers of genomic rNMPs (see Supplementary Table S5), in order to account for DNA breaks that may occur during alkaline heat-treatment independently of incorporated ribonucleotides, the number of DNA breaks in the WT strain (sample 1, not represented on the plot and small table) was subtracted from every other Rnr1 [+] strain (samples 3, 7, 9, 13, 15, 19 and 21), and the number of DNA breaks in the single mutant *P_GAL_:3HA-RNR1* depleted of Rnr1 (sample 2, not represented on the plot and small table) was subtracted from every other Rnr1 [−] strain (samples 4, 8, 10, 14, 16, 20 and 22).

To gain more insight into the role of Rnr1 depletion in increasing the incorporation of rNMPs in genomic DNA, we sought to determine the numbers of genomic rNMPs in mutants lacking both Rnr1 and RER, or that are also combined with an rNTP-permissive Pol in presence/absence of Top1. We made use of a panel of pairs of strains (see organigram in Figure 5A), of which each pair comprises one strain harboring the *RNR1* gene under the control of its native promoter and expressing Rnr1 at WT levels (henceforth referred to as ‘Rnr1 [+]’; odd numbers on the organigram), and an equivalent strain carrying *P_GAL_:3HA-RNR1* and depleted of Rnr1 for 6 h in glucose-containing medium (henceforth referred to as ‘Rnr1 [−]’; even numbers on the organigram). Total cellular DNA was heated in the presence of alkali, which should both denature the DNA duplex and hydrolyze the backbone downstream of embedded ribonucleotides (see e.g. (20,23)). Alkali-fragments from total DNA (Afts) were resolved by alkaline-gel electrophoresis (Figure 5A). Based on densitometry of SYBR-staining, we determined for each strain the distribution of Afts sizes (i.e. densitometry normalized to the length of the fragment; Figure 5B and Supplementary Figure S4), and the numbers of rNMPs present in the 24 Mb haploid yeast genome (i.e. ‘total genomic rNMPs’; Figure 5C and Supplementary Table S5). For the calculation of the numbers of total genomic rNMPs, in order to account for DNA breaks that may occur independently of incorporated ribonucleotides during alkaline heat-treatment, the number of DNA breaks in the WT strain was subtracted from every other Rnr1 [+] strain. Replicating DNA may be more susceptible to breakage during alkaline heat-treatment than the rest of the genome, so the number of DNA breaks in the single mutant *P_GAL_:3HA-RNR1* depleted of Rnr1, which shows severe blockage of cells in S-phase (Figure 1E), was subtracted from every other Rnr1 [−] strain.

As previously reported (see e.g. (53)), total genomic rNMPs were increased in all three double mutants *pol1-L868M rnh201*Δ (Rnr1 [+]), *pol3-L612M rnh201*Δ (Rnr1 [+]) and *pol2-M644G rnh201*Δ (Rnr1 [+]), compared to single mutant *rnh201*Δ (Rnr1 [+]) (Figure 5C, compare samples 7, 13 and 19 with 3; for *P*-values, see Supplementary Table S5). Moreover, compared to the two double mutants *pol1-L868M rnh201Δ* (Rnr1 [+]) and *pol3-L612M rnh201Δ* (Rnr1 [+]), total genomic rNMPs were not increased by loss of Top1 in the two corresponding triple mutants *pol1-L868M rnh201Δ top1Δ* (Rnr1 [+]) and *pol3-L612M rnh201Δ top1Δ* (Rnr1 [+]). However, loss of Top1 mildly increased rNMP retention in the triple mutant *pol2-M644G rnh201Δ top1*Δ (Rnr1 [+]) relative to *pol2-M644G rnh201Δ* (Rnr1 [+]) (Figure 5C, compare samples 7 with 9, and 13 with 15, and 19 with 21; for *P*-values, see Supplementary Table S5). Together, these results are consistent with earlier studies reporting that Top1-mediated processing of unrepaired single genomic rNMPs is mainly associated with ribonucleotides incorporated by Pol ϵ in leading strand (37,54,73,74,85).

The abundance of genomic rNMPs in the strain *P_GAL_:3HA-RNR1 rnh201Δ* (Rnr1 [−]) (sample 4: 8403 ± 280 rNMPs) was much greater than in the strain *rnh201Δ* (Rnr1 [+]) (sample 3: 1651 ± 275 rNMPs), as determined by the increase in alkali-fragmentation (Figure 5A and B, Supplementary Figure S4, and Figure 5C [for *P*-value, see Supplementary Table S5]). Fragmentation was further exacerbated in the three triple mutants *P_GAL_:3HA-RNR1 pol1-L868M rnh201*Δ (Rnr1 [−]) (sample 8: 15 007 ± 876 rNMPs), *P_GAL_:3HA-RNR1 pol3-L612M rnh201Δ* (Rnr1 [−]) (sample 14: 20 136 ± 1334 rNMPs) and *P_GAL_:3HA-RNR1 pol2-M644G rnh201*Δ (Rnr1 [−]) (sample 20: 18 316 ± 582 rNMPs) (Figure 5A, lower-part of Figure 5B, Supplementary Figure S4B and Figure 5C [for *P*-values, see Supplementary Table S5]). Accordingly, there was a noticeable enrichment of fragments <400nt in the distribution of Afts sizes in these triple mutants (lower-part of Figure 5B, and Supplementary Figure S4B; mode of peak ∼350–400nt, ∼300–350nt and ∼200–250nt for samples 8, 14 and 20, respectively).

The increased alkali-fragmentation observed in genomic DNA from *P_GAL_:3HA-RNR1 rnh201Δ* (Rnr1 [−]) mutants without or with an rNTP-permissive Pol (ϵ-M644G, δ-L612M or α-L868M) could potentially reflect incomplete DNA replication/repair, independently of incorporated ribonucleotides (Figure 5A, lower-part of Figure 5B, Supplementary Figure S4B, Figure 5C and Supplementary Table S5, samples 4, 8, 10, 14, 16, 20 and 22). To assess this, formamide-denatured genomic DNA was resolved on non-denaturing, neutral 1% agarose gels (Supplementary Figure S5). This revealed that *P_GAL_:3HA-RNR1 rnh201Δ* (Rnr1 [−]) mutants without or with an rNTP-permissive Pol have intact genomic DNA, similar to single mutant *P_GAL_:3HA-RNR1* (Rnr1 [−]) (Supplementary Figure S5, compare samples 13, 16, 19 and 22 with 9). Moreover, genomic DNA from *P_GAL_:3HA-RNR1 rnh201Δ* (Rnr1 [−]) mutants without or with an rNTP-permissive Pol showed fragmentation only after *in vitro* treatment with recombinant *E. coli* RNase HII, which preferentially incises at sites of DNA-embedded ribonucleotides as compared to the RNA moiety of RNA/DNA hybrids (86) (Supplementary Figure S5, compare samples 14 with 13, and 17 with 16, and 20 with 19, and 23 with 22). In contrast, genomic DNA from the WT strain or the single mutant *P_GAL_:3HA-RNR1* (Rnr1 [−]) was not fragmented by treatment with RNase HII (Supplementary Figure S5, compare samples 1 with 2, and 9 with 10). As a control of formamide-denaturation of genomic DNA, both the WT strain and the single mutant *P_GAL_:3HA-RNR1* (Rnr1 [−]) showed fragmentation of their genomic DNA after incubation with the DNA nicking endonuclease Nb.BtsI (Supplementary Figure S5, compare samples 1 with 4, and 9 with 12). Together, these results indicate that most Afts detected in *P_GAL_:3HA-RNR1 rnh201Δ* (Rnr1 [−]) mutants without or with an rNTP-permissive Pol are due to cleavage at sites of genome-embedded ribonucleotides (Figure 5A, lower-part of Figure 5B, Supplementary Figure S4B, Figure 5C and Supplementary Table S5, samples 4, 8, 10, 14, 16, 20 and 22).

Collectively, the results presented above led us to conclude that genomic rNMPs are greatly increased in the presence of limited dNTP concentrations in RER-deficient strains depleted of Rnr1, and this is further exacerbated in cells that also harbor an rNTP-permissive Pol.

Even though Afts of shorter sizes accumulated in the triple mutant *P_GAL_:3HA-RNR1 pol2-M644G rnh201Δ* (Rnr1 [−]) as compared to the triple mutant *P_GAL_:3HA-RNR1 pol3-L612M rnh201Δ* (Rnr1 [−]), the amounts of total genomic rNMPs, were, unexpectedly, similar for both mutants (compare samples 20 with 14 in Figure 5A, lower-part of Figure 5B, and Supplementary Figure S4B, with same samples in Figure 5C [for *P*-values, see Supplementary Table S5]). To gain more insight into this question, we calculated the contributions of replicative Pols α, δ, and ϵ to the synthesis of genomic DNA, by applying the mathematical formula from Reijns *et al.* (53), for four different conditions (Supplementary Figure S6 and Table S6): (i) in presence of both Rnr1 and Top1. (ii) in absence of Rnr1 and presence of Top1. (iii) in presence of Rnr1 and absence of Top1. (iv) in absence of both Rnr1 and Top1. For the description of the mathematical formula, see sub-section ‘Calculation of the contributions of replicative Pols α, δ and ϵ to synthesis of *S. cerevisiae* nuclear genome’ in Materials and Methods. The slightly higher contribution of Pol δ relative to Pol ϵ to synthesis of genomic DNA in cells expressing Rnr1 at WT levels (conditions i and iii) is consistent with the notion that Pol δ, besides synthesizing the bulk of lagging strand, plays a role in leading strand synthesis (see e.g. (5)). The minor contribution of Pol α, regardless of the state of Rnr1 (conditions i–iv), is in agreement with the role of Pol α in synthesizing only a small fraction of the yeast nuclear genome (see e.g. (53)). Notably, the contribution of Pol δ was ∼3-fold higher than Pol ϵ in cells depleted of Rnr1 (conditions ii and iv). This suggests that Pol ϵ might synthesize much less leading strand DNA under limited dNTP availability, with Pol δ taking a larger role. Both triple mutants *P_GAL_:3HA-RNR1 pol2-M644G rnh201Δ* (Rnr1 [−]) and *P_GAL_:3HA-RNR1 pol3-L612M rnh201Δ* (Rnr1 [−]) have similar levels of dNTP pools and activation of the S-phase checkpoint (Supplementary Figure S3A, compare samples 8 with 9, and Supplementary Figure S3B, compare lanes h with l, Rnr3 and P-Rad53). This excluded the possibility that the lower contribution of Pol ϵ versus Pol δ in absence of Rnr1 could be due to differences in levels of dNTP pools and/or S-phase checkpoint activation.

Finally, we observed no significant differences in the distribution of Afts sizes or amounts of total genomic rNMPs for the three quadruple mutants *P_GAL_:3HA-RNR1 pol1-L868M rnh201Δ top1Δ* (Rnr1 [−]), *P_GAL_:3HA-RNR1 pol3-L612M rnh201Δ top1*Δ (Rnr1 [−]) and *P_GAL_:3HA-RNR1 pol2-M644G rnh201*Δ*top1*Δ (Rnr1 [−]) with respect to their corresponding triple mutant, *TOP1^+^* counterparts (compare samples 10 with 8, and 16 with 14, and 22 with 20 in Figure 5A, lower-part of Figure 5B, Supplementary Figure S4B, and Figure 5C [for *P*-values, see Supplementary Table S5]). This was unexpected, since the loss of Top1 reversed the severe growth defects of RER-deficient mutants that are depleted of Rnr1 and contain Pol δ-L612M or ϵ-M644G (see Figure 4A) and will be addressed in the following section.

### Top1 processes unrepaired single genomic ribonucleotides in both leading and lagging strands under conditions of limited dNTP concentrations

**Figure 6.**
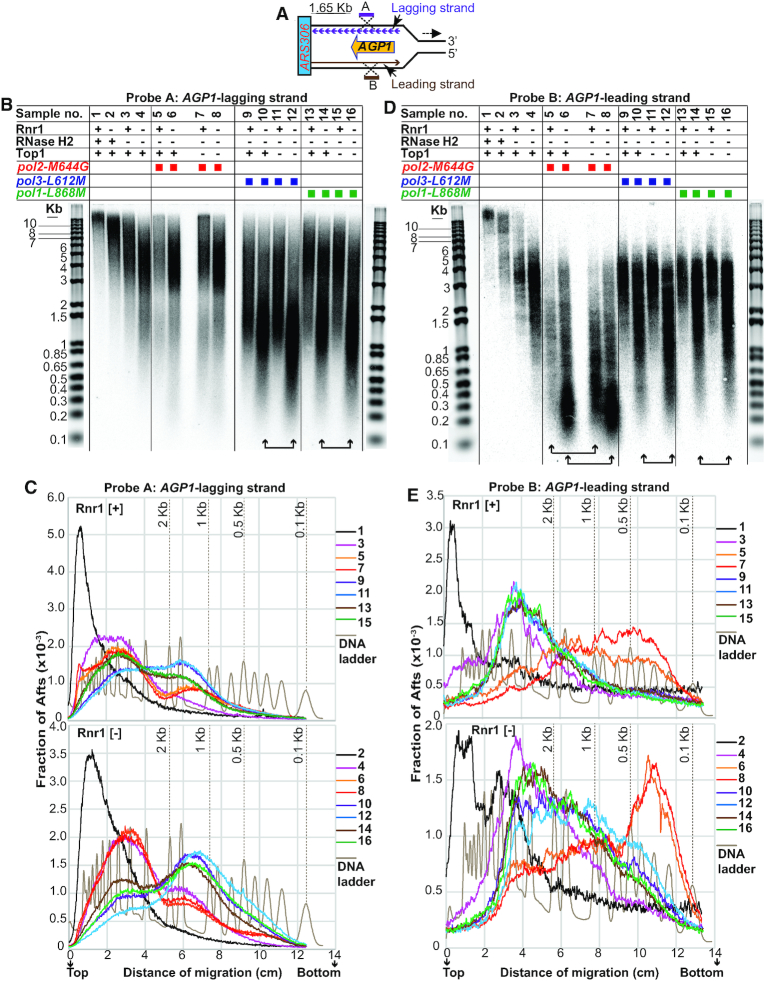
Southern analyses showing that Top1 cleaves genomic ribonucleotides in Rnr1-depleted RER-deficient triple mutants bearing Pol ϵ-M644G, δ-L612M or α-L868M. (**A**) Scheme depicting the RF encompassing the locus *AGP1*, which is located at ∼1.65 Kb distance from the right side of the bidirectional origin *ARS306* on chromosome III (the direction of replication, left to right, is indicated by a black horizontal arrow). For clarity, the RF to the left side of *ARS306* is omitted. OFs in the nascent lagging strand, which are synthesized by Pols α and δ, are depicted by small purple arrows. The nascent leading strand, which is mainly synthesized by Pol ϵ, is depicted by a long brown arrow. Template lagging and leading strands are depicted by black lines. Single-stranded probes A and B (length ∼660 nt) hybridize to *AGP1*-lagging strand DNA (bottom strand) and *AGP1*-leading strand DNA (top strand), respectively, at ∼2 Kb distance from the right side of *ARS306* (for primer sequences see Supplementary Table S2). (**B–E**) The following strains (see Supplementary Table S1 for the list of strains) are represented by symbols on the organigrams in (B) and (D). Rnr1 [+] condition: 1. WT; 3. *rnh201*Δ; 5. *pol2-M644G rnh201*Δ; 7. *pol2-M644G rnh201Δ top1*Δ; 9. *pol3-L621M rnh201*Δ; 11. *pol3-L621M rnh201*Δ*top1*Δ; 13. *pol1-L868M rnh201*Δ; 15. *pol1-L868M rnh201Δ top1*Δ. Rnr1 [−] condition: 2. *P_GAL_:3HA-RNR1*; 4. *P_GAL_:3HA-RNR1 rnh201*Δ; 6. *P_GAL_:3HA-RNR1 pol2-M644G rnh201*Δ; 8. *P_GAL_:3HA-RNR1 pol2-M644G rnh201*Δ*top1*Δ; 10. *P_GAL_:3HA-RNR1 pol3-L621M rnh201*Δ; 12. *P_GAL_:3HA-RNR1 pol3-L621M rnh201Δ top1*Δ; 14. *P_GAL_:3HA-RNR1 pol1-L868M rnh201*Δ; 16. *P_GAL_:3HA-RNR1 pol1-L868M rnh201Δ top1*Δ. For the description of Rnr1 [+] and Rnr1 [−] conditions and symbols on the organigrams see legend of Figure 5A. Alkali-treated DNA samples were separated on two alkaline 1% agarose gels (5 μg DNA per lane and per gel). The gels were neutralized, stained with SYBR gold (see pictures in Supplementary Figure S7) and capillary-blotted. Each membrane was hybridized with one radiolabeled probe (see also Material and Methods). Represented is an example of two independent repeats. (**B**) Blot showing Southern hybridization of *AGP1*-lagging strand DNA with probe A. For the ease of comparison, SYBR-stained DNA ladders from the picture of the gel represented in Supplementary Figure S7A were superimposed on the image of the blot. Selected molecular weights are labeled in kb on the left-hand ladder. Double-arrowed horizontal bars on the bottom of the blot point towards prominent differences between Top1+ and Top1– strains. The vertical lines along the image of the blot are included for clarity. (**C**) Signal densitometry histograms of samples in (B). Densitometry of radioactive signal was measured at 0.01 mm intervals and the background subtracted within each lane. To account for differences in loading between DNA samples, the densitometry of each interval was normalized to the sum of densitometries of all intervals within each lane. Normalized values are plotted on the Y-axis as a fraction of Afts. Distances of migration of Afts on the gel are plotted on the X-axis. For the ease of comparison, histograms for Rnr1 [+] and Rnr1 [−] conditions are represented separately in the upper- and lower-plot, respectively. The densitometry histogram of the DNA ladder is superimposed on each plot. Selected DNA molecular weights are labeled in Kb. The distance (in cm) and direction (top to bottom) of migration of Afts are indicated below the X-axis of the lower-plot. (**D**) Blot showing Southern hybridization of *AGP1*-leading strand DNA with probe B. SYBR-stained DNA ladders from the picture of the gel in Supplementary Figure S7B were superimposed on the image of the blot. For other details, see (B). (**E**) Signal densitometry histograms of samples in (D). For other details, see (C).

We analyzed the role of Top1 in the processing of unrepaired single genomic rNMPs in Rnr1-depleted RER-deficient triple mutants bearing Pol ϵ-M644G, δ-L612M or α-L868M, by strand-specific Southern blotting of Afts (see e.g. (48,54,77,80,81,87)). DNA was processed as for Figure 5, resolved by alkaline-gel electrophoresis and capillary-blotted. We analyzed the gene *AGP1*, which is situated nearby the strong early-firing bidirectional origin *ARS306* (88) (see scheme in Figure 6A; see also Supplementary Table S2 for the chromosomal coordinates of the probes). Single-stranded PCR probes were complementary to either the lagging strand (bottom strand) (probe A), or leading strand (top strand) (probe B).

In Figure 6, we compare strains harboring the *RNR1* gene under the control of its native promoter and expressing Rnr1 at WT levels (Rnr1 [+]), to strains carrying *P_GAL_:3HA-RNR1* and depleted of Rnr1 for 6 h (Rnr1 [−]) (see organigram in Figure 6B and D). In Supplementary Figures S8–S10, we used the same strains, but for strains carrying *P_GAL_:3HA-RNR1* we included also growth in permissive conditions, in which Rnr1 should be moderately over-expressed (henceforth referred to as ‘Rnr1 [*]’) (see organigram in panels B and D of Supplementary Figures S8–S10).

Compared to the WT strain, both alkali-treated *AGP1*-lagging and *AGP1*-leading strand DNAs were more fragmented in the single mutant *rnh201Δ* (Rnr1 [+]), and much more fragmented following Rnr1 depletion in *P_GAL_:3HA-RNR1 rnh201Δ* (Rnr1 [−]) (Figure 6, compare samples 1, 3 and 4; Supplementary Figures S8–S10, compare samples 1, 4 and 6). These results indicate that the accumulation of rNMPs in both leading and lagging strands of *AGP1* is greater in RER-deficient mutant depleted of Rnr1.

For the *AGP1*-lagging strand, DNA was more fragmented in *pol1-L868M rnh201Δ* (Rnr1 [+]), relative to *rnh201Δ* alone, and much more in *pol3-L612M rnh201Δ* (Rnr1 [+]) (Figure 6B and upper-part of Figure 6C, compare samples 13 and 9 with 3; panels B and C in Supplementary Figures S8 and S9, compare samples 10 with 4). Moreover, high-mobility Afts <1000 nt accumulated from *AGP1*-leading strand in *pol2-M644G rnh201*Δ (Rnr1 [+]), and the fragmentation was further exacerbated in the triple mutant counterpart *pol2-M644G rnh201*Δ *top1Δ* (Figure 6D and upper-part of Figure 6E, compare samples 5 with 3, and 7 with 5; Supplementary Figure S10D and E, compare samples 10 with 4, and 13 with 10). Together, these results are in agreement with an earlier report that analyzed the *URA3* reporter gene integrated at the *AGP1* locus (54).

Alkali-fragmentation of the *AGP1*-lagging strand was greater for *P_GAL_:3HA-RNR1 pol1-L868M rnh201Δ* (Rnr1 [−]), relative to *P_GAL_:3HA-RNR1 rnh201Δ* (Rnr1 [−]), with further increases in fragmentation in *P_GAL_:3HA-RNR1 pol3-L612M rnh201Δ* (Rnr1 [−]) (Figure 6B and lower-part of Figure 6C, compare samples 14 with 4, and 10 with 14; panels B and C in Supplementary Figures S8 and S9, compare samples 12 with 6). Notably, *AGP1*-lagging strand fragmentation was even more pronounced in the absence of Top1, in the two quadruple mutants *P_GAL_:3HA-RNR1 pol1-L868M rnh201Δ top1Δ* (Rnr1 [−]) and *P_GAL_:3HA-RNR1 pol3-L612M rnh201Δ top1Δ* (Rnr1 [−]) (Figure 6B and lower-part of Figure 6C, compare samples 16 with 14, and 12 with 10; panels B and C in Supplementary Figures S8 and S9, compare samples 15 with 12).

Together, the results presented above for strains bearing the alleles *pol1-L868M* and *pol3-L612M* lead us to hypothesize that embedded rNMPs increased on the *AGP1*-lagging strand in the quadruple mutants *P_GAL_:3HA-RNR1 pol1-L868M rnh201Δ top1Δ* (Rnr1 [−]) and *P_GAL_:3HA-RNR1 pol3-L612M rnh201Δ top1Δ* (Rnr1 [−]), relative to their corresponding triple mutants *TOP1^+^* equivalents. In contrast, there was no increase in embedded rNMPs on the *AGP1*-lagging strand in the triple mutants *pol1-L868M rnh201Δ top1Δ* (Rnr1 [+]) and *pol3-L612M rnh201Δ top1Δ* (Rnr1 [+]), relative to their corresponding double mutants *TOP1^+^* equivalents, which is consistent with a previous report (54) (Figure 6B and upper-part of Figure 6C, compare samples 15 with 13, and 11 with 9; panels B and C in Supplementary Figures S8 and S9, compare samples 13 with 10). We presume this difference reflects a much higher density of genomic ribonucleotides in the mutants depleted of Rnr1, which is above the limit for detection of Top1-mediated incisions by Southern analysis. We, therefore, conclude that Top1 processes unrepaired single genomic rNMPs that are incorporated by Pols α and δ in lagging strand at the *AGP1* locus in absence of Rnr1.

Alkali-fragmentation of the *AGP1*-leading strand revealed that high mobility fragments <500 nt accumulated in the strain *P_GAL_:3HA-RNR1 pol2-M644G rnrh201Δ* (Rnr1 [−]), compared to the corresponding strain *pol2-M644G rnrh201Δ* (Rnr1 [+]) (Figure 6D and E, compare samples 6 with 5; Supplementary Figure S10D and E, compare samples 12 with 10). Additionally, alkali-fragmentation was higher in the quadruple mutant *P_GAL_:3HA-RNR1 pol2-M644G rnrh201Δ top1Δ* (Rnr1 [−]) relative to the corresponding triple mutant expressing Top1 (Figure 6D and lower-part of Figure 6E, compare samples 8 with 6; Supplementary Figure S10D and E, compare samples 15 with 12). These results further confirm the elevated incorporation of rNMPs by Pol ϵ-M644G following Rnr1 depletion and support the model that Top1 processes unrepaired single rNMPs in leading strand (54).

Compared to the double mutant *P_GAL_:3HA-RNR1 rnh201Δ* (Rnr1 [−]), alkali-treated *AGP1*-leading strand DNA was more fragmented in the triple mutants *P_GAL_:3HA-RNR1 pol1-L868M rnh201*Δ (Rnr1 [−]) and *P_GAL_:3HA-RNR1 pol3-L612M rnh201*Δ (Rnr1 [−]), and even more in their corresponding quadruple mutants lacking Top1. The greatest fragmentation was seen in the quadruple mutant *P_GAL_:3HA-RNR1 pol3-L612M rnh201Δ top1Δ* (Rnr1 [−]) (Figure 6D and lower-part of Figure 6E, compare samples 14 with 4, and 10 with 14, and 16 with 14, and 12 with 10; panels D and E in Supplementary Figures S8 and S9, compare samples 6, 12 and 15). These results suggest that rNMPs are inserted by Pol α-L868M or δ-L612M in *AGP1*-leading strand in absence of Rnr1 and are processed by Top1 when RNase H2 is also absent.

Finally, there was a clear decrease in alkali-fragmentation of both *AGP1*-lagging and *AGP1*-leading strand DNAs in various combinations of RER-deficient mutants carrying *P_GAL_:3HA-RNR1* that were grown in permissive conditions, in which Rnr1 should be moderately over-expressed (Rnr1 [*]) (Supplementary Figures S8B, S8D, S9B, S9D and S10D, compare samples 11 with 10 and 12, and 14 with 13 and 15). Overexpression of Rnr1 may be associated with higher dNTP concentrations (59,60), supporting the expectation that dNTP levels and rNMP incorporation in DNA are inversely correlated, which is consistent with a previous report (13).

## DISCUSSION

Herein, we report the effects of high density of unrepaired ribonucleotides in genomic DNA of *S. cerevisiae*. We have modulated two factors, which influence rNMP incorporation: (i) The dNTP:rNTP ratios, by depleting Rnr1, which controls both the levels and balance of dNTPs (see Figure 1C, Supplementary Figure S3A and Supplementary Table S4). (ii) The sugar selectivity of replicative Pols, by employing steric gate Pol variants that are rNTP-permissive (for reviews, see e.g. (11,12)). We also deleted the gene *RNH201* or both genes *RNH201* and *TOP1*. Here we found that genomic rNMPs and the associated Top1-mediated Δ2–5 bp are greatly increased in double mutants lacking Rnr1 and RER. Furthermore, when a threshold of single genomic rNMPs is exceeded in RER-deficient Rnr1-depleted cells that also harbor an rNTP-permissive Pol (ϵ-M644G or δ-L612M), Top1-mediated RNA–DNA damage leads to severe growth defects. Moreover, we show that under constitutive replicative stress induced by Rnr1 depletion, loss of RNases H1 and H2 leads to cell lethality, likely due to deleterious accumulation of RNA/DNA hybrids (e.g. R-loops). Finally, we provide evidence showing that the contribution of Pol δ to synthesis of genomic DNA is much greater than that of Pol ϵ in cells depleted of Rnr1.

### Use of variants RNase H2-RED and RNase H2-G42S elucidates the growth defects associated with depletion of Rnr1 in the absence of RNase H activity

Multiple studies have shown that the absence of RNase H2, and to a lesser extent RNase H1, induces genomic instability in *S. cerevisiae* (see e.g. (22,23,74,76,82,89–91)). Both RNase H2 activities, hybrid-removal and RER, play important roles in safeguarding genome integrity, confounding assignment of a phenotype to either function.

In our drop test growth assays, the use of the three plasmids expressing WT Rnh201, variant Rnh201-RED, or variant Rnh201-G42S, led us to infer that the accumulation of RNA/DNA hybrids is the critical factor for cell lethality in strain *P_GAL_:3HA-RNR1 rnh1Δ rnh201Δ* depleted of Rnr1 (Figure 2B).

It is established that the accumulation of R-loops in budding and fission yeasts double mutants *rnh1Δ rnh201Δ* compromises genome stability (see e.g. (27,91–98)). We propose that R-loop-mediated transcription-replication conflicts (for reviews, see e.g. (29,30,99)) lead to defects in DNA replication, genome stability and growth in strains depleted of Rnr1 and lacking RNases H1 and H2. Limited dNTP pools, induced by Rnr1 depletion, could compromise the restart of stalled RFs at sites of persistent R-loops (98,100), thereby leading to RF collapse/breakage, and/or block repair of R-loop induced DNA damage, e.g. by the HR-repair machinery that would escort RFs (for a review, see e.g. (101)). Because RNR supplies also dNTPs for DNA repair, we speculate that insufficient dNTP supply may additionally compromise repair of R-loop associated-DNA damage independently of DNA replication in cells depleted of Rnr1 (see e.g. (102)).

Rnh201-RED suppressed the growth defects induced by Rnr1 depletion to similar levels in absence of RNase H2 or RNases H1 and H2 (Figure 2B). This suggests that either endogenous RNase H1 is dispensable to resolve RNA/DNA hybrids, or that only a subset of hybrids is toxic and these are specifically recognized by Rnh201-RED. However, Rnh201-G42S, which has much lower hybrid-removal activity than Rnh201-RED (70), suppressed less well the growth defects of strain *P_GAL_:3HA-RNR1 rnh1Δ rnh201Δ* in glucose medium than did Rnh201-RED (Figure 2B). This suggests a possible overlap between RNases H1 and H2 in hybrid removal in conditions of constitutive replicative stress following Rnr1 depletion. Nonetheless, it is important to emphasize that RNases H1 and 2 have different substrates preferences that do not completely overlap. There are R-loops targeted exclusively by RNase H1 (91), and conversely RNase H2 has R-loop substrates that RNase H1 is not able to cleave even when overexpressed, but that the variant Rnh201-RED can degrade (70).

Because the variant Rnh201-RED was almost as effective as WT Rnh201 in strains *P_GAL_:3HA-RNR1 rnh201Δ* and *P_GAL_:3HA-RNR1 rnh1Δ rnh201Δ* in glucose-containing medium (Figure 2B), it may be that increased accumulation of unrepaired single genomic rNMPs in these strains has only a slight negative effect on their growth. An alternative possibility is that the accumulation of unrepaired single genomic rNMPs (and the associated Top1-mediated RNA-DNA damage) in strain *P_GAL_:3HA-RNR1 rnh1Δ rnh201Δ* in glucose-containing medium greatly exacerbated formation of RNA/DNA hybrids, e.g. R-loops, and/or their impact on genome stability and cell growth. This is in contrast to the situation that occurs in strains *P_GAL_:3HA-RNR1 pol2-M644G rnh201*Δ and *P_GAL_:3HA-RNR1 pol3-L612M rnh201*Δ where the variant Rnh201-RED failed to support growth in glucose medium (Figure 4B), suggesting that high density of unrepaired single genomic rNMPs, and not RNA/DNA hybrids or a combination of both substrates, caused severe growth defects in these strains.

Finally, Meroni *et al.* (103) suggested that the translesion synthesis DNA polymerase η incorporates multiple contiguous rNMPs that are toxic for genome stability and viability of cells lacking RNases H1 and H2 under limited dNTP pools triggered by HU. We found that the deletion of the gene *RAD30* encoding for yeast Pol η in strain *P_GAL_:3HA-RNR1 rnh1Δ rnh201Δ* (background BY4741) only modestly alleviated cell lethality in glucose medium (our unpublished observations). We therefore hypothesize that, unlike R-loops, genome-embedded multiple contiguous rNMPs may not accumulate at high levels and/or may not be highly toxic in strains depleted of Rnr1 and lacking RNases H1 and H2.

### Following Rnr1 depletion Pol δ contributes much more to nuclear genome synthesis than Pol ϵ

We assessed the contribution of replicative Pols α, δ and ϵ to overall DNA synthesis (Supplementary Figure S6 and Supplementary Table S6). We found that Pol δ synthesizes only slightly more DNA than Pol ϵ in the presence of Rnr1, supporting previously reported *in vivo* data in budding and fission yeasts (see e.g. (4,5)). Interestingly, however, we found that Pol δ synthesizes ∼3-fold more DNA than Pol ϵ in Rnr1-depleted cells. In accordance with this result, Southern analysis of alkali-treated genomic DNA detected increased fragmentation of *AGP1*-leading strand in the triple mutant *P_GAL_:3HA-RNR1 pol3-L612M rnh201Δ* (Rnr1 [−]) compared to the double mutant *P_GAL_:3HA-RNR1 rnh201Δ* (Rnr1 [−]), with even greater fragmentation in the quadruple mutant *P_GAL_:3HA-RNR1 pol3-L612M rnh201Δ top1Δ* (Rnr1 [−]) (Figure 6 and Supplementary Figure S9).

These results are consistent with recent *in vitro* experiments (3), reporting that leading strand synthesis at *ARS306* is initiated by Pol δ. This uses lagging strand primers, which are laid down by Pol α-primase complex at RFs on opposite sides of the origin. Notably, our probe B (∼660 nt length) hybridizes to *AGP1*-leading strand DNA ∼2 kb from *ARS306* (Figure 6A and Supplementary Table S2). Moreover, *in vitro* analyses show that purified Pol ϵ requires 2- to 4-fold higher dNTP levels for maximum extension of nascent leading strand compared to Pol δ (104). We hypothesize that, following Rnr1 depletion, the hand-off from Pol δ to Pol ϵ during initiation of leading-strand synthesis at bidirectional origins is delayed by insufficient dNTP supply. It is important to emphasize that probe B detected excessive fragmentation of *AGP1*-leading strand DNA in strains *P_GAL_:3HA-RNR1 pol2-M644G rnh201Δ* (Rnr1 [−]) and *P_GAL_:3HA-RNR1 pol2-M644G rnh201Δ top1Δ* (Rnr1 [−]) (Figure 6 and Supplementary Figure S10), suggesting that the delay in the hand-off from Pol δ to Pol ϵ might occur only in a subset of cells within the same strain.

Finally, Pol δ may not only act to initiate leading strand synthesis at origins, but might function in other regions in the genome; e.g. difficult-to-replicate sequences and sites of mismatches, or under conditions of stress (see e.g. (6–9,105,106)). We further hypothesize that, following Rnr1 depletion, Pol ϵ may frequently uncouple from the nascent, leading strand 3′ terminus. Pol δ would then take over DNA synthesis until coupled leading strand synthesis by Pol ϵ is re-established.

### Top1-processing of unrepaired single genomic rNMPs is observable by Southern blotting in both leading and lagging strands under high rNMP load

Processing of unrepaired single genomic rNMPs by Top1 was previously reported to predominantly occur on the leading strand, with much less activity on the lagging strand (see e.g. (37,54,73,74,85)). Williams *et al.* (54) showed by Southern blotting that Top1 activity at sites of unrepaired rNMPs is observable in leading strand in strain *pol2-M644G rnh201Δ*, but not in lagging strand in strains *pol1-L868M rnh201Δ* and *pol3-L612M rnh201Δ*, which all three express Rnr1 at WT levels (results recapitulated herein in Figure 6 and Supplementary Figures S8–S10). Our Southern analyses using mutants depleted of Rnr1 showed, as expected, that processing of unrepaired single genomic rNMPs by Top1 is observable in leading strand in *P_GAL_:3HA-RNR1 pol2-M644G rnh201Δ* (Rnr1 [−]) (Figure 6 and Supplementary Figure S10). Notably, Top1 activity at sites of rNMPs was observable in both leading and lagging strands in *P_GAL_:3HA-RNR1 pol1-L868M rnh201Δ* (Rnr1 [−]), and much more in *P_GAL_:3HA-RNR1 pol3-L612M rnh201Δ* (Rnr1 [−]) (Figure 6 and Supplementary Figures S8 and S9). This leads us to hypothesize that the combination of Rnr1 depletion, lack of RNase H2 and presence of Pol δ-L612M or α-L868M increased the rNMP load and Top1-dependent rNMP removal in both leading and lagging strands in these triple mutants, sufficiently for detection of the retained rNMPs by Southern analysis in the corresponding *top1Δ* quadruple mutants counterparts. However, the density of ribonucleotides and Top1-mediated cleavage at unrepaired rNMP sites in leading strand of strains harboring Pol ϵ-M644G and lacking RNase H2 was sufficiently high for detection of retained rNMPs by Southern analysis, regardless of the presence/absence of Rnr1, as previously suggested by Williams *et al.* (54) for cells expressing Rnr1 at WT levels. Taken together, we conclude that Top1 incises at unrepaired single genomic rNMPs on leading and lagging strands. However, there is a detection limit for the identification of Top1 cleavages by Southern analyses.

### *S. cerevisiae* has a threshold of tolerance to unrepaired genomic ribonucleotides

The incorporation of ribonucleotides in genomic DNA of mice lacking RNase H2 (RNase H2^null^), or expressing the variant RED (RNase H2^RED^) causes massive genome instability, leading to a p53 DNA damage response and early embryonic death (19–21). The threshold for embryonic cell death was estimated at ≤240K unrepaired rNMPs per 1 Gb of genomic DNA (21).

Here, we found that yeast strains *P_GAL_:3HA-RNR1 pol2-M644G rnh201Δ* (Rnr1 [−]) and *P_GAL_:3HA-RNR1 pol3-L612M rnh201Δ* (Rnr1 [−]) showed severe growth defects in a Top1-dependent manner (Figure 4A), with rNMPs at a density of ∼763K (±24K) and ∼839K (±55K) per 1 Gb of DNA, respectively (Figure 5C and Supplementary Table S5; for the ease of comparison between yeast and mammalian genomes, values of rNMPs are given per 1 Gb of DNA). In contrast, lower densities of genomic rNMPs were relatively well tolerated; in strains *P_GAL_:3HA-RNR1 rnh201Δ* (Rnr1 [−]) (350K ±12K Gb^−1^) and *P_GAL_:3HA-RNR1 pol1-L868M rnh201Δ* (Rnr1 [−]) (625K ± 36K Gb^−1^), although both strains grew slower than single mutant *P_GAL_:3HA-RNR1* (Rnr1 [−]) (Figures 4A and 5C and Supplementary Table S5).

Even though the two triple mutants *P_GAL_:3HA-RNR1 pol2-M644G rnh201*Δ (Rnr1 [−]) and *P_GAL_:3HA-RNR1 pol3-L612M rnh201*Δ (Rnr1 [−]) have only ∼20–25% more rNMPs than the triple mutant *P_GAL_:3HA-RNR1 pol1-L868M rnh201*Δ (Rnr1 [−]), the growth of the former two strains is much more compromised than the latter strain (Figures 4A and 5C and Supplementary Table S5). We can connect these findings to indicate that total genomic rNMPs in strain *P_GAL_:3HA-RNR1 pol1-L868M rnh201*Δ (Rnr1 [−]) do not exceed a threshold above which Top1-induced RNA-DNA damage leads to severe growth defects. We speculate that a substantial fraction of ribonucleotides incorporated by Pol α and their associated Top1-mediated un-ligatable nicks in cells lacking both RER and Rnr1 are removed by the OF maturation machinery, and/or by the mismatch repair mechanisms, which are very efficient in correcting errors generated by Pol α (53,54,107).

Top1-induced-DSBs at unrepaired single genomic rNMP sites in strain *pol2-M644G rnh201Δ* are not tolerated in absence of Rad52 or presence of HU (37,77), albeit this strain accumulates ∼590K ± 156K rNMPs per 1 Gb according to (53) (∼400K ±11K rNMPs Gb^−1^ in our strain background, Figure 5C and Supplementary Table S5), which is below the number of rNMPs we observed in strains *P_GAL_:3HA-RNR1 pol2-M644G rnh201Δ* (Rnr1 [−]) and *P_GAL_:3HA-RNR1 pol3-L612M rnh201Δ* (Rnr1 [−]). It remains, therefore, to be determined whether the severe growth defects in strains expressing Pol ϵ-M644G or δ-L612M in absence of both RNase H2 and Rnr1 arise because the level of Top1-mediated-DSBs at single genomic ribonucleotide sites exceeds the capacity of Rad51/Rad52-dependent HR repair. The machinery may be swamped by high numbers of DSBs and/or be compromised by insufficient dNTP supply induced by Rnr1 depletion (see model in Supplementary Figure S11).

We conclude that in budding yeast, unrepaired single genomic rNMPs above a critical threshold lead to deleterious DNA damage in a Top1-dependent manner, particularly in conditions of insufficient dNTP supply (e.g. under Rnr1 depletion or HU). DNA lesions induced by human TOP1 at sites of genomic ribonucleotides in transformed, human RNase H2-deficient cells are efficiently repaired by Poly (ADP-ribose) polymerase (PARP) (108). However, when these cancerous cells were treated with PARP-trapping drugs, unresolved TOP1-mediated DNA lesions led to deleterious defects in DNA replication, genome integrity and cell viability (108). RNase H2-depleted human cells are non-viable in the presence of inhibitors of ATR^Mec1^ (ATR is the mammalian homolog of yeast Mec1), likely due to deleterious Top1-mediated RNA-DNA damage (109). It is tempting to speculate that inhibition of ATR^Mec1^ in these RNase H2-depleted cells (109) led to insufficient dNTP supply (for reviews on mammalian RNR regulation, see e.g. (110,111)), thereby both increasing incorporation of rNMPs in genomic DNA by replicative Pols and compromising the repair of the associated DNA damage. Finally, we hypothesize that the threshold of tolerance to single unrepaired genomic rNMPs in mice embryos lacking RER (19–21) might be dependent on the deleterious effects of TOP1.

### Modulating RNR activity could be beneficial in human diseases associated with RNase H deficiencies

Silencing of *RRM2*, the gene encoding for mammalian RNR complex subunit R2, hyper-sensitizes cancer cells to camptothecin, an inhibitor of TOP1, by hampering repair of DNA damage caused by collisions of RFs with TOP1 cleavage complexes (112). Indeed, inhibitors of mammalian RNR activity are widely used as chemotherapeutic agents to reduce the dNTP supply that is needed for uncontrolled proliferation in cancer cells (for reviews, see e.g. (110,113)). Our findings in budding yeast leads us to hypothesize that, apart from impeding DNA synthesis (both replication and repair) in cancer cells, RNR inhibitors would have the under-appreciated effect of increasing the incorporation of rNMPs in genomic DNA. Targeted inhibition of RNR activity in cancer cells defective for RNase H2 might hyper-sensitize them to PARP-trapping drugs, which hamper the repair of TOP1-mediated DNA lesions at sites of single genomic rNMPs (108,114,115). The cytotoxicity of the combination of RNR and PARP inhibitors could be even further enhanced in cancer cells defective for RNase H2 and also bearing an rNTP-permissive Pol mutator (for a review, see e.g. (116)). We also posit that RNR inhibitors would potentiate R-loop-driven DNA damage in cancer cells lacking RNase H2 and/or RNase H1 ((117,118); for a review, see e.g. (111)). Contrary to cells harboring hypomorphic RNases H, however, cancer cells with WT or hypermorphic RNases H could respond less well to RNR inhibitors. We suggest that cancerous tumors could be tested for defects in RNases H in order to identify those with hypomorphic RNase H mutations as bonafide candidates for treatment with RNR inhibitors (109), and/or to improve anti-RNR strategies and reduce their toxicity.

Increased gene dosage of *RRM2* alleviated DNA damage defects in ATR^Mec1^-depleted mice (119), and supplementing cells with nucleosides alleviated replication-induced DNA damage in cancer cells (120). We anticipate that increasing dNTP levels exogenously might have a protective effect on proliferating cells in AGS patients with hypomorphic mutations in the RNase H2 complex, by alleviating DNA damage associated with unrepaired single genomic rNMPs and/or R-loops (121,122). RNR might therefore be a promising opportunity to explore in AGS therapies.

## Supplementary Material

gkaa103_Supplemental_Files

## Notes

Present address: Jaime Iranzo, Centro de Biotecnología y Genómica de Plantas, Universidad Politécnica de Madrid (UPM) - Instituto Nacional de Investigación y Tecnología Agraria y Alimentaria (INIA), Madrid, Spain.
