# Supplementary material for: High density of unrepaired genomic ribonucleotides leads to Topoisomerase 1-mediated severe growth defects in absence of ribonucleotide reductase
Source: Nucleic Acids Res. 2020 Mar 18;48(8):4274–97. doi: 10.1093/nar/gkaa103 (PMC7192613; doi:10.1093/nar/gkaa103)
Supplement: gkaa103_Supplemental_Files [file gkaa103_supplemental_files.zip › Supplementary Data.docx]

**Supplementary Data for**

**High density of unrepaired genomic ribonucleotides leads to Topoisomerase 1-mediated severe growth defects in absence of ribonucleotide reductase**

Susana M. Cerritelli^1^, Jaime Iranzo^2^, Sushma Sharma^3^, Andrei Chabes^3^, Robert J. Crouch^1^, David Tollervey^4^ and Aziz El Hage^4*^

^1^ SFR, Division of Intramural Research, *Eunice Kennedy Shriver* National Institute of Child Health and Human Development, National Institutes of Health, Bethesda, Maryland, USA

^2^ National Center for Biotechnology Information, National Library of Medicine, National Institutes of Health, Bethesda, Maryland 20894, USA. Current address: Centro de Biotecnología y Genómica de Plantas, Universidad Politécnica de Madrid (UPM) - Instituto Nacional de Investigación y Tecnología Agraria y Alimentaria (INIA), Madrid, Spain.

^3^ Department of Medical Biochemistry and Biophysics, Umeå University, Umeå SE-901 87 Sweden.

^4^ The Wellcome Centre for Cell biology, University of Edinburgh, Edinburgh, UK.

* Correspondence should be addressed to AEH (aziz.elhage@ed.ac.uk)

Keywords: Replicative DNA polymerase; RNase H; ribonucleotide; RNA/DNA hybrids; Topoisomerase 1; Ribonucleotide reductase.

**Table of contents:**

**Supplementary Figures**

Figure S1: Simplified overview of the regulation of the activity of yeast RNR complex by the S-phase checkpoint kinase cascade Mec1-Rad53-Dun1.

Figure S2: Analyses by RT-qPCR of mRNAs encoding proteins of the RNR complex in strains depleted of Rnr1 in the presence or absence of Crt1.

Figure S3: Measurement of dNTP levels, western-blotting and drop test growth assays.

Figure S4: Averaged distributions of Afts sizes.

Figure S5: Resolution on non-denaturing gel of formamide-denatured genomic DNA from Rnr1-depleted RER-deficient mutants without or with an rNTP-permissive Pol.

Figure S6: Contributions of replicative Pols α, δ and ε to synthesis of *S. cerevisiae* nuclear genome, in presence or absence of Rnr1.

Figure S7: SYBR-stained gels for Southern-blots in Figure 6.

Figure S8: Southern analyses of RER-deficient Rnr1-depleted *TOP1+*/*top1Δ* mutants bearing Pol α-L868M.

Figure S9: Southern analyses of RER-deficient Rnr1-depleted *TOP1+*/*top1Δ* mutants bearing Pol δ-L612M.

Figure S10: Southern analyses of RER-deficient Rnr1-depleted *TOP1+*/*top1Δ* mutants bearing Pol ε-M644G.

Figure S11: Model depicting single genomic rNMPs and the associated Top1-incisions in Rnr1-depleted RER-deficient triple mutants bearing Pol ε-M644G or δ-L612M.

**Supplementary Tables S1-S3**

Table S1: List of strains and plasmids.

Table S2: List of oligonucleotides.

Table S3: Values of total and specific *CAN1* mutation rates.

**Supplementary Tables S4-S6** (provided as a single, separate, Excel file)

Table S4: dNTP concentrations (related to Figure 1C and Supplementary Figure S3A).

Table S5: Calculations of the average of total genomic rNMPs (related to Figure 5C).

Table S6: Calculations of the contributions of replicative Pols α, δ and ε to synthesis of S. cerevisiae nuclear genome (related to Supplementary Figure S6).

**Supplementary Figures**

***
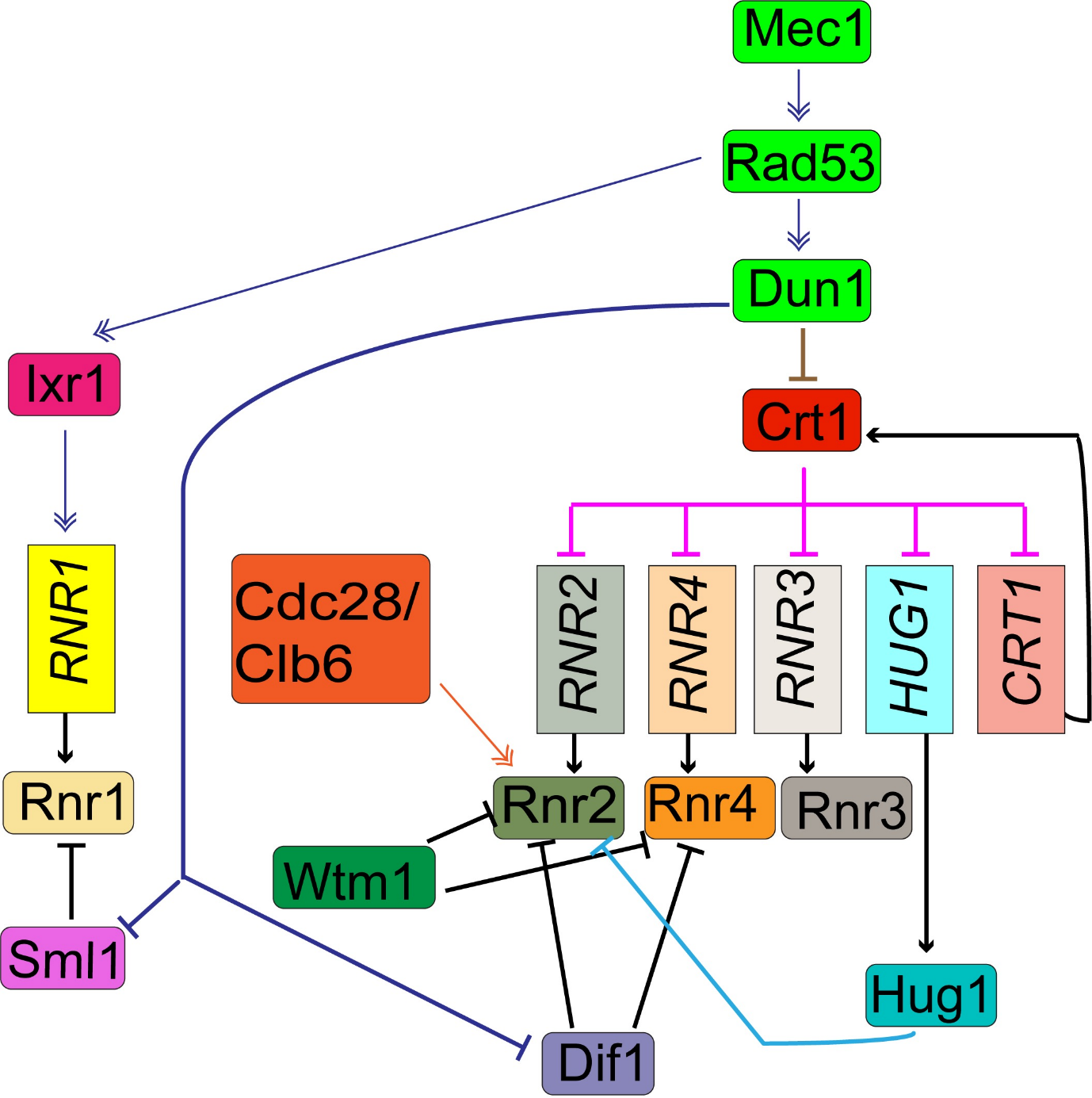
*Supplementary Figure S1. Simplified overview of the regulation of the activity of yeast RNR complex by the S-phase checkpoint kinase cascade Mec1-Rad53-Dun1.**This cartoon is adapted from Tsaponina *et al.* (1). Here, we present a brief overview of the pathway that regulates the expression, localization and activity of yeast RNR (for an extensive overview see e.g. (2)). In normally cycling/unperturbed WT cells: In all cell cycle stages, the transcription factor Crt1 represses the expression of the genes *RNR2-4*, *HUG1* and its own gene *CRT1* (3,4). Moreover, in cells that are not replicating their DNA (referred to as ‘outside the S-phase’), Sml1 binds to Rnr1 in the cytoplasm and inhibits its activity (5), and Dif1 binds to the heterodimer Rnr2-Rnr4 and transports it from the cytoplasm to the nucleus (6), which in turn leads to the sequestration of Rnr2-Rnr4 by Wtm1 in the nucleus (7). This separates the subunits of the RNR complex in different cellular compartments, thereby hampering the formation of the active RNR holoenzyme, which normally occurs in the cytoplasm. However, in cells undergoing G1/S-phase transition, the S-phase cyclin-cyclin-dependent kinase (CDK) complex Clb6-Cdc28 re-localizes Rnr2-Rnr4 from the nucleus to the cytoplasm (8), and in cells replicating their DNA (referred to as ‘unperturbed S-phase’), inherent activation of the DNA damage and replication checkpoint (referred to in the manuscript as ‘S-phase checkpoint’) kinase cascade Mec1-Rad53-Dun1 leads to Dun1 phosphorylation, which in turn induces partial degradation of Sml1 (9,10). These mechanisms lead to the formation of the active RNR holoenzyme in the cytoplasm. In cells subjected to genotoxic stress: The S-phase checkpoint kinase cascade Mec1-Rad53-Dun1 is highly activated, which in turn induces robust Dun1-mediated-degradation of both Sml1 and Dif1 (6,11), and Dun1-mediated-inactivation of Crt1. These mechanisms lead to strong upregulation of RNR activity. In unperturbed S-phase or under genotoxic stress: Mec1 and Rad53 activate the transcription factor Ixr1 (independently of Dun1 and Crt1), which in turn leads to the upregulation of the expression of the gene *RNR1* (1). Additionally, by the end of unperturbed S-phase or genotoxic stress, Hug1 inhibits RNR activity by binding directly to Rnr2 in order to limit dNTP synthesis (4). Note that a recent report by Li *et al.* (12) described a new branch in the S-phase checkpoint pathway (omitted on the cartoon), downstream of Mec1-Rad53. Briefly, under replicative stress (e.g. in presence of HU), the kinase Mck1, together with Dun1, antagonizes the repressor function of Crt1 via phosphorylation, thereby allowing the de-repression of transcription of the genes *RNR2-4* and *HUG1*. If replicative stress is severe, McK1 inhibits the transcription of the gene *HUG1*, in a Crt1-independent manner, in order to maintain high levels of RNR activity.

Key symbols and shapes

Rectangle with straight or round corners: Gene or protein, respectively. Orange-two-headed-arrow: Activates during G1/S-phase transition in normally cycling/unperturbed WT cells. Dark-blue-two-headed-arrow: Activates in unperturbed S-phase or under genotoxic stress. Dark-blue-arrow-to-bar: Represses Sml1 in unperturbed S-phase and both Sml1 and Dif1 under genotoxic stress. Brown-arrow-to-bar: Represses under genotoxic stress. Black-arrow-to-bar: Represses outside the S-phase in normally cycling/unperturbed WT cells. Light-blue-arrow-to-bar: Represses by the end of unperturbed S-phase or genotoxic stress. Fuchsia-arrow-to-bar: Represses in all cell cycle stages in normally cycling/unperturbed WT cells. Black arrow: Gene encodes for protein.


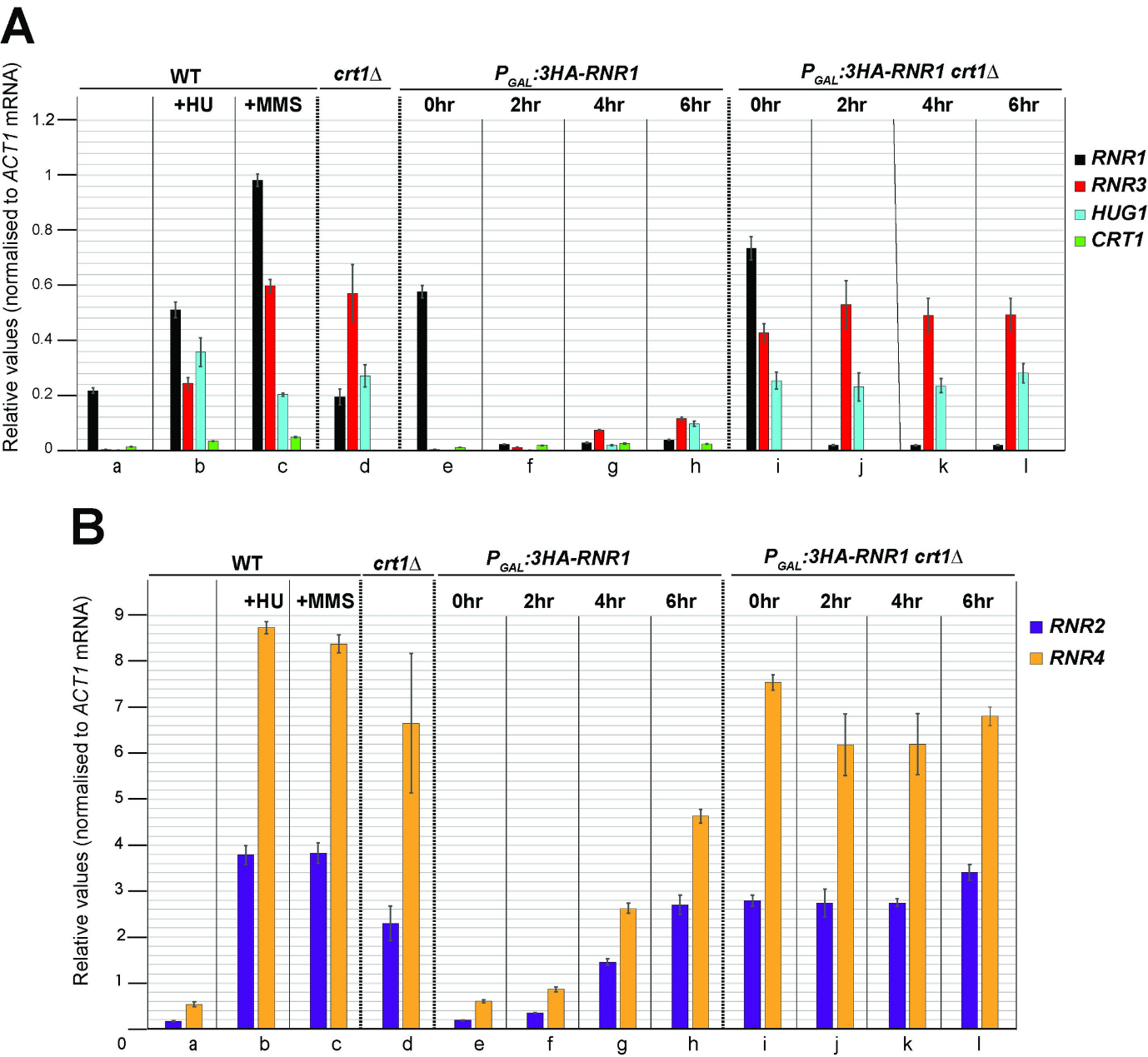


**Supplementary Figure S2. Analyses by RT-qPCR of mRNAs encoding proteins of the RNR complex in strains depleted of Rnr1 in the presence or absence of Crt1.** (**A**, **B**).Samples are from the same cell cultures that were used for western-blotting and FACS in Figure 1B, 1D and 1E. For strains and growth conditions see legend of Figure 1B. qPCR values normalized to *ACT1* mRNA, which is set to 1, are plotted on the Y-axis. For the ease of comparison, mRNAs *RNR1*, *RNR3*, *HUG1* and *CRT1* are represented in (A), and mRNAs *RNR2* and *RNR4* are represented in (B). A unique Latin letter is allocated for each sample. Represented on the plots are averages with S.E.M. (number of independent experiments: 3 for samples a, d and i-l; 2 for samples b and c; 5 for samples e-h).

**
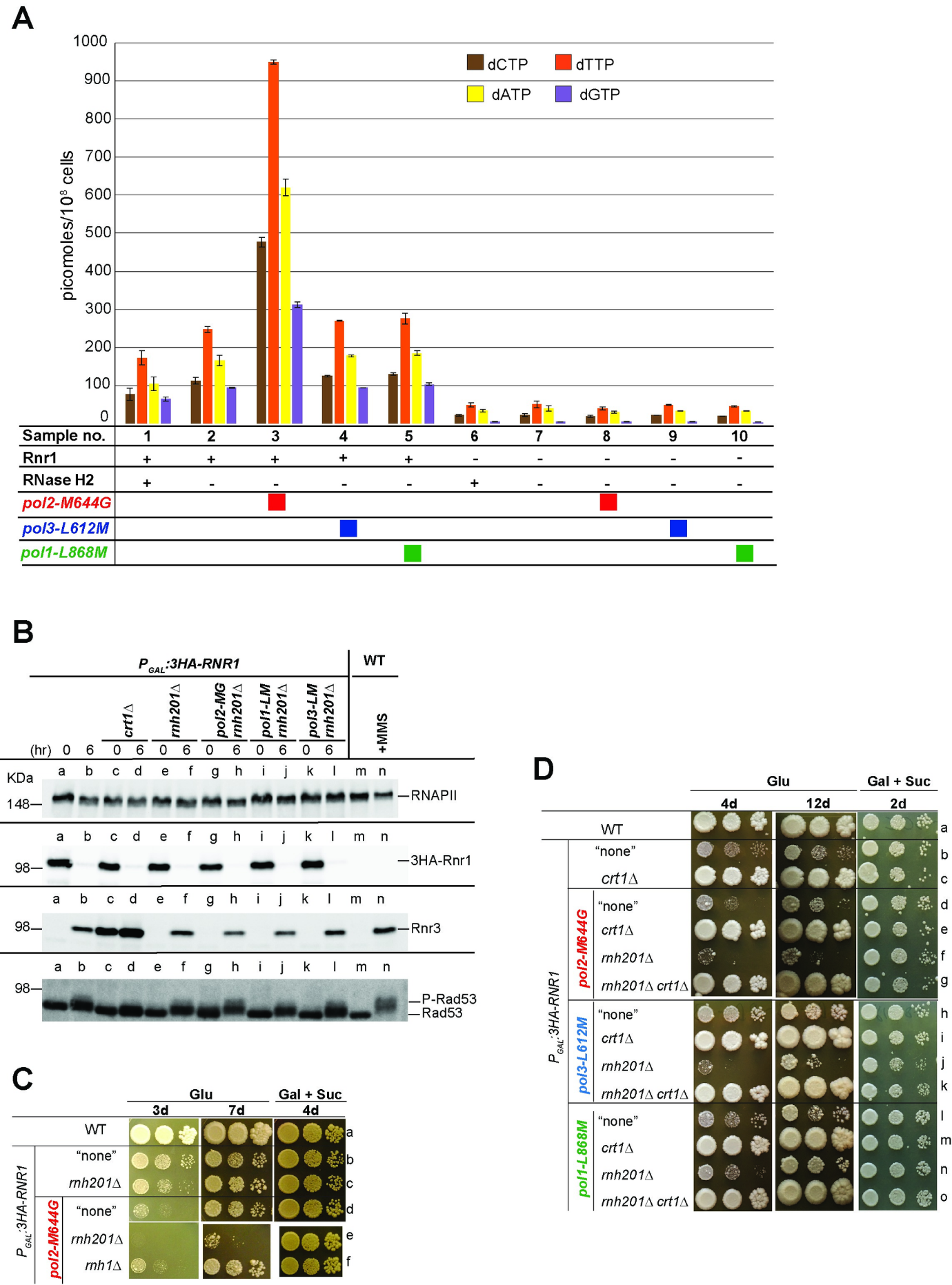
Supplementary Figure S3. Measurement of dNTP levels, western-blotting and drop test growth assays**. (**A**). Measurement of dNTP concentrations in various combinations of mutants expressing Rnr1 at WT levels or depleted of Rnr1. The following strains are represented by symbols on the organigram below the bar plot (see also Supplementary Table S1 for the list of strains): 1. WT. 2. *rnh201*Δ. 3. *pol2-M644G rnh201*Δ. 4. *pol3-L621M rnh201*Δ. 5. *pol1-L868M rnh201*Δ. 6. *P_GAL_:3HA-RNR1*. 7. *P_GAL_:3HA-RNR1 rnh201* 8. *P_GAL_:3HA-RNR1 pol2-M644G rnh201*Δ. 9. *P_GAL_:3HA-RNR1 pol3-L621M rnh201*Δ. 10. *P_GAL_:3HA-RNR1 pol1-L868M rnh201*Δ*.* Strains expressing Rnr1 at WT levels (samples 1-5) were cultured in rich YEPD medium (2% glucose) at 30°C and harvested at OD_600_ ~0.4. Strains carrying *P_GAL_:3HA-RNR1* (samples 6-10) were grown at 30°C in liquid minimal medium lacking histidine with 2% galactose and 2% sucrose. To trigger Rnr1 depletion, cells at OD_600_ ~0.2 were transferred to minimal medium lacking histidine with 2% glucose and maintained in exponential phase. Cells were harvested 6 hr after transfer to glucose-containing medium at OD_600_ ~0.4 (see also Material and Methods). dNTP levels were normalized to rNTP levels and values were adjusted to the total number of cells used for the preparation (see also Supplementary Table S4). Error bars reflect S.E.M. of 2 independent repeats. Symbols on the organigram: + and - indicate that the protein is present or absent, respectively; alleles *pol2-M644G*, *pol3-L612M* and *pol1-L868M* are depicted by red, blue and green squares, respectively. For the ease of comparison, WT strain (sample 1) and single mutant *P_GAL_:3HA-RNR1* (sample 6) are also represented in Figure 1C. (**B**). Analysis of Rnr1 depletion and activation of the S-phase checkpoint by western blotting in various combinations of mutants carrying *P_GAL_:3HA-RNR1*. Strains *P_GAL_:3HA-RNR1*, *P_GAL_:3HA-RNR1 crt1Δ, P_GAL_:3HA-RNR1 rnh201Δ*, *P_GAL_:3HA-RNR1 rnh201Δ pol2-M644G*, *P_GAL_:3HA-RNR1 rnh201Δ* *pol1-L868M*,and *P_GAL_:3HA-RNR1 rnh201Δ* *pol3-L612M*, were grown as described in (A) and cells were harvested before transfer (0 hr) and 6 hr after transfer to glucose-containing medium. As a control for the activation of S-phase checkpoint, the WT strain was grown in rich YEPD (2% glucose) medium at 30°C, in absence of drugs (labelled “none”), or in presence of 0.03% MMS for 3 hr (labelled +MMS) (see also Material and Methods). Total proteins were separated on a 6.5% SDS-polyacrylamide gel and then electro-blotted. The same filter was probed separately with antibodies against RNA polymerase II (RNAPII) as loading control, 3HA-Rnr1, Rnr3 and Rad53 (P-Rad53 represents the phosphorylated form of Rad53). Relevant protein molecular weights (KDa) are indicated at the left-hand. For the ease of comparison, each well is allocated a unique Latin letter, which is repeated in each sub-panel. The length of Rnr1 depletion in hours (hr) is indicated above wells a-l. One representative experiment is shown of 2 independent ones. (**C**). Loss of RNase H1 in Rnr1-depleted mutants bearing Pol ε-M644G does not lead to severe growth defects. Drop test growth assays of strain WT, and strains carrying *P_GAL_:3HA-RNR1* without gene deletion (labelled “none”), or with deletion of the gene *RNH201*, and strains carrying both *P_GAL_:3HA-RNR1* and allele *pol2-M644G*, without gene deletion (labelled “none”), or with deletion of the gene *RNH201* or *RNH1*. Cells were grown in YPGS (2% galactose and 2% sucrose) liquid medium at 30°C overnight. Serial-dilutions were plated on YEPD (2% glucose) and YPGS solid media. Plates were incubated at 30°C. Photographs were taken at the indicated number of days (d). Experiments were repeated independently 2 times. “Glu” stands for glucose and “Gal + Suc” stands for galactose plus sucrose. For the ease of comparison, each row is allocated a unique Latin alphabet letter. The images for each incubation time are from the same plate. The horizontal lines across the images are included for clarity. For the list of strains see Supplementary Table S1. Both panels (C) and (D) are related to Figure 4. (**D**). Rnr1-depleted RER-deficient mutants bearing Pol ε-M644G or δ-L612M grow like the WT strain in absence of Crt1. Drop test growth assays of strain WT, strains carrying *P_GAL_:3HA-RNR1* without gene deletion (labelled “none”), or with deletion of the gene *CRT1*, and strains carrying both *P_GAL_:3HA*-*RNR1* and allele *pol2-M644G*, *pol3-L612M*, or *pol1-L868M*, without gene deletion (labelled “none”), or with deletion of the gene *CRT1* or *RNH201*, or both genes *RNH201* and *CRT1*. Cells were grown in YPGS (2% galactose and 1% sucrose) liquid medium at 30°C overnight, and serial-dilutions were plated on YEPD (2% glucose) and YPGS solid media. Experiments were repeated independently 3 times. For other details, see panel (C).

**
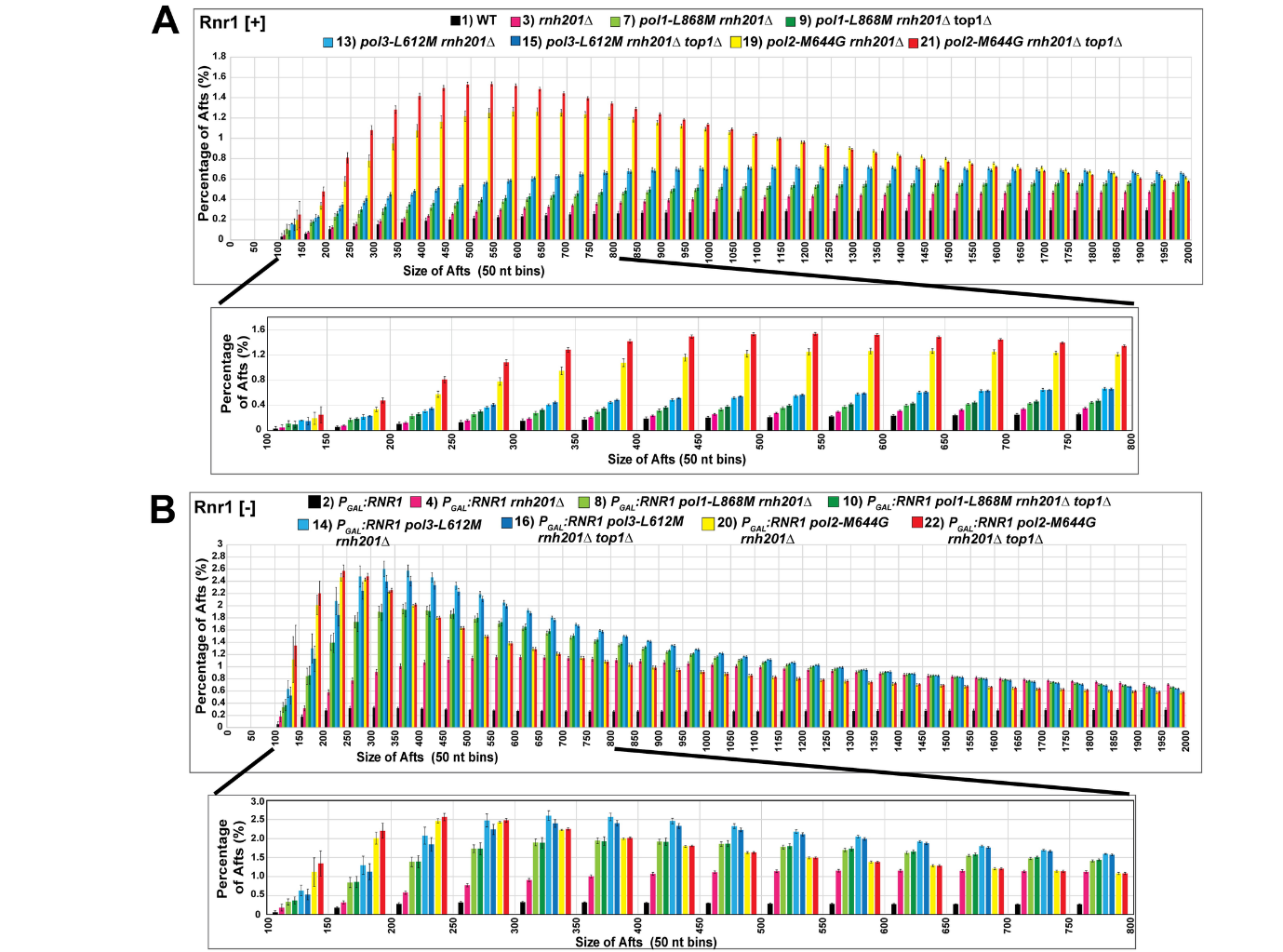
**

**Supplementary Figure S4. Averaged distributions of Afts sizes.** (**A**). Histograms of Afts sizes in Rnr1 [+] condition (Rnr1 expression at WT levels). Sample numbering, strains and data are the same as for Figure 5B upper-plot (samples 5, 11 and 17 are omitted). Bins covering 50 nt of Afts sizes (from 0-1999 nt) are plotted on the X-axis, and the percentage of Afts sizes from each bin (%) is plotted on the Y-axis. Note that for the ease of comparison, Afts ≥ 2000 nt were not included, and Afts sizes in bins 0 and 50 could not be accounted for. Below is an enlargement of bins covering Afts sizes 100-800 nt. For mathematical modelling of the data see the section “Quantitation of genomic ribonucleotides” in Material and Methods. Represented are averages of 4 independent repeats with S.E.M. Note that only 1 repeat is represented in Figure 5B upper-plot. (**B**). Histograms of Afts sizes in Rnr1 [-] condition (Rnr1 depletion). Sample numbering, strains and data are the same as for Figure 5B lower-plot (samples 6, 12 and 18 are omitted). Represented are averages of 4 independent repeats with S.E.M. Note that only 1 repeat is represented in Figure 5B lower-plot. For other details, see panel (A).

**
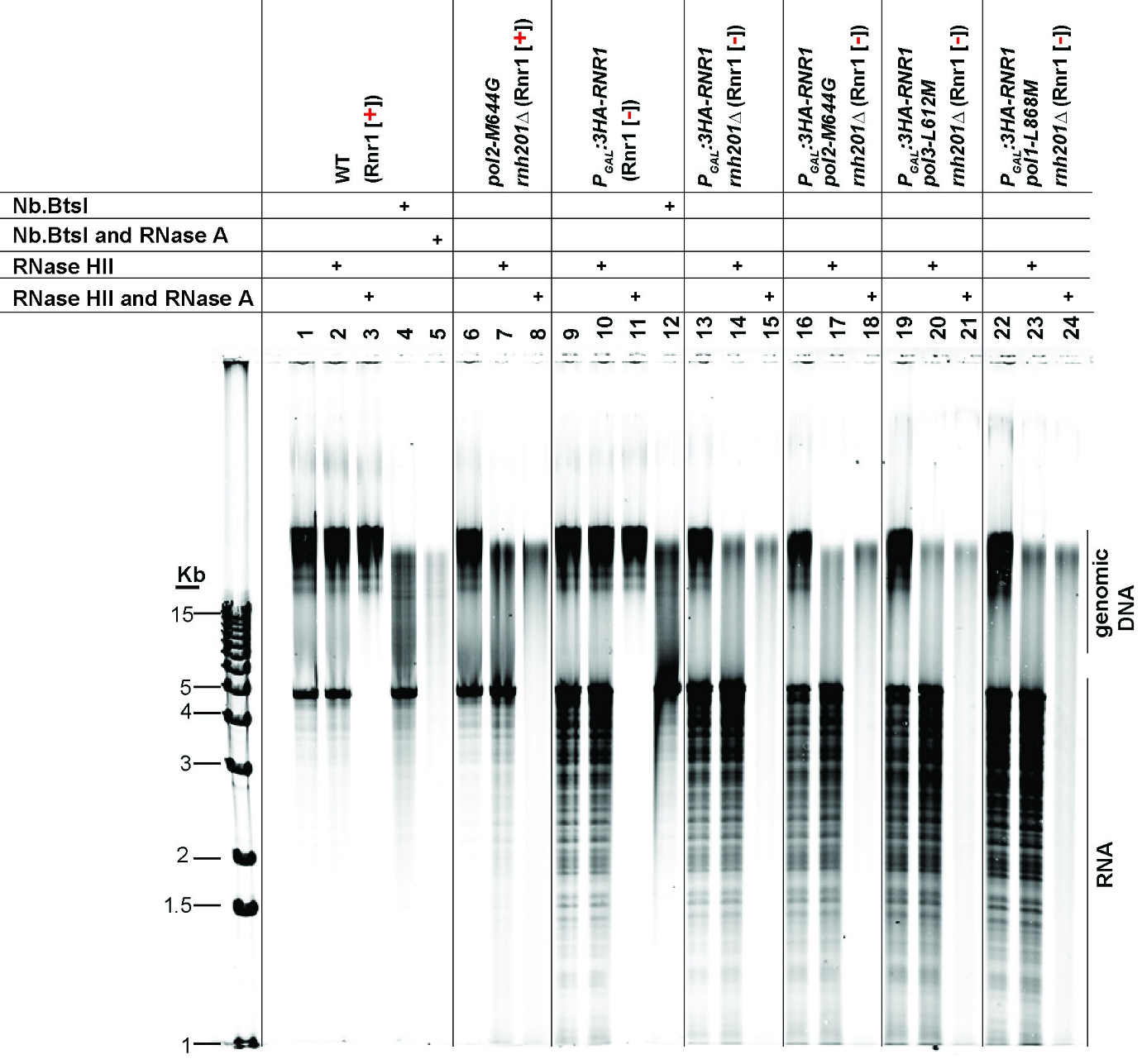
**

**Supplementary Figure S5. Resolution on non-denaturing gel of formamide-denatured-genomic DNA from Rnr1-depleted RER-deficient mutants without or with an rNTP-permissive Pol.** For Rnr1 [**+**] condition (Rnr1 expression at WT levels): Strains WT (DNA samples 1-5) and *pol2-M644G rnh201Δ* (DNA samples 6-8) were cultured in rich YEPD medium (2% glucose) at 30°C and harvested in exponential phase. For Rnr1 [**-**] condition (Rnr1 depletion): Strains *P_GAL_:3HA-RNR1* (DNA samples 9-12), *P_GAL_:3HA-RNR1 rnh201Δ* (DNA samples 13-15), *P_GAL_:3HA-RNR1* *pol2-M644G* *rnh201Δ* (DNA samples 16-18), *P_GAL_:3HA-RNR1* *pol3-L612M* *rnh201Δ* (samples 19-21), and *P_GAL_:3HA-RNR1* *pol1-L868M rnh201Δ* (DNA samples 22-24), were cultured as described in legend of Supplementary Figure S3A and harvested 6 hr after transfer to glucose-containing media. Total DNA was extracted from yeast cells and then incubated in the presence of RNase A in 1X TE with high salt (0.5M NaCl), for 1 hr at 25°C (RNase A with high salt selectively degrades single-stranded RNA, while avoiding degradation of double-stranded RNA, the RNA moiety of RNA/DNA hybrids and genome-embedded single ribonucleotides; see e.g. (13-16)). DNA aliquots were incubated at 37°C for 2 hr in 1X ThermoPol buffer, either in the absence of enzymes (samples 1, 6, 9, 13, 16, 19 and 22), or in the presence of recombinant *E. coli* RNase HII (samples 2, 7, 10, 14, 17, 20 and 23; ’**+**’ on organigram), or in the presence of both recombinant *E. coli* RNase HII and RNase A (samples 3, 8, 11, 15, 18, 21 and 24; ’**+**’ on organigram) (*E. coli* RNase HII preferentially incises at sites of DNA-embedded ribonucleotides as compared to the RNA moiety of RNA/DNA hybrids; see e.g. (17)) (RNase A with no/low salt degrades single-stranded RNA, double-stranded RNA, the RNA moiety of RNA/DNA hybrids and genome-embedded single ribonucleotides; see e.g. (13-16)). As a control for DNA denaturation, DNA aliquots were incubated at 37°C for 1 hr in 1X CutSmart buffer, either in the presence of the DNA nicking endonuclease Nb.BtsI (samples 4 and 12; ’**+**’ on organigram), or in the presence of both Nb.BtsI and RNase A (sample 5; ’**+**’ on organigram). DNA samples were denatured in the presence of 90% formamide and 20 mM EDTA, pH8, at 37°C for 1 hr, then separated, together with the non-denatured DNA ladder, on a neutral, non-denaturing 1% agarose gel. The gel was subsequently stained with SYBR gold. For a detailed description of the methodology see Material and Methods. Selected molecular weights of the DNA ladder are indicated to the left of the gel. The vertical lines along the image of the gel are included for clarity. One representative experiment is shown of at least four independent ones. RNA species (labelled ’RNA’ to the right of the gel), which were not degraded by RNase A in 1X TE buffer with high salt (samples 1, 2, 4, 6, 7, 9, 10, 12, 13, 14, 16, 17, 19, 20, 22 and 23), but which were degraded by RNase A in 1X ThermoPol or CutSmart buffer (samples 3, 5, 8, 11, 15, 18, 21 and 24), may represent double-stranded RNA (see e.g. (15)).

**
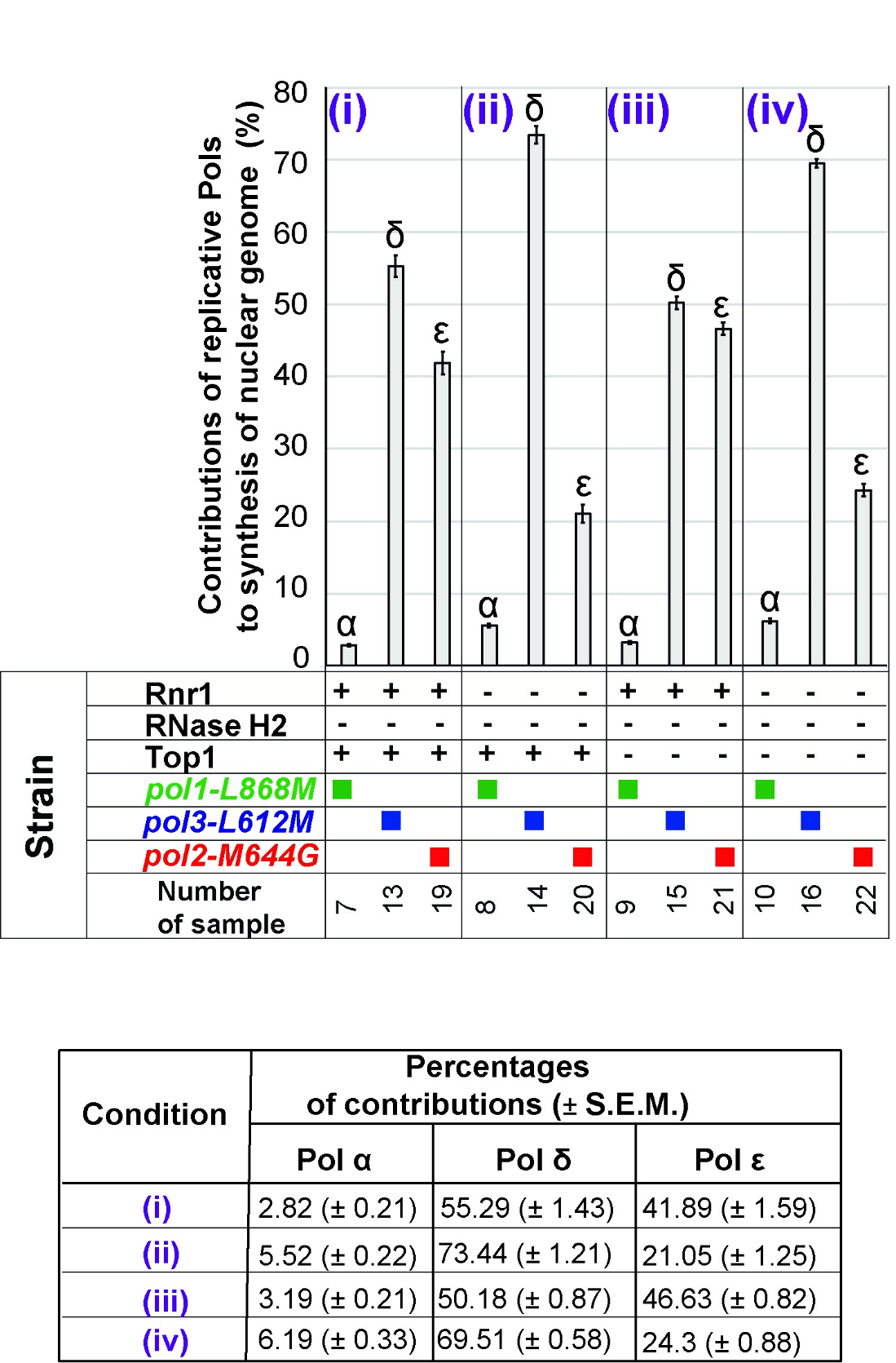
**

**Supplementary Figure S6. Contributions of replicative Pols α, δ and ε** **to synthesis of *S. cerevisiae* nuclear genome, in presence or absence of Rnr1.** This Supplementary Figure is related to Figure 5C and Supplementary Tables S5 and S6.The percentages of contributions (%) of WT replicative Pols α, δ and ε to synthesis of *S. cerevisiae* genome are plotted on the Y-axis with S.E.M. Greek letters α, δ and ε on the top of the bars on the plot represent Pols α, δ and ε, respectively. The contributions were calculated in four different conditions (indicated by violet Roman numerals on the plot): (i) in presence of both Rnr1 and Top1. (ii) in absence of Rnr1 and presence of Top1. (iii) in presence of Rnr1 and absence of Top1. (iv) in absence of both Rnr1 and Top1. For the ease of comparison, the percentages of contributions with S.E.M in four different conditions are indicated in the small table below the organigram. For the calculations of the contributions, we applied the mathematical formula ‘(N_ΔPolx_/F_Polx_)/([N_ΔPol α-L868M_/F_Pol α-L868M_] + [N_ΔPol δ-L612M_/F_Pol δ-L612M_] + [N_ΔPol ε-M644G_/F_Pol ε-M644G_])’, from Reijns *et al.* (18) (see Supplementary Table S6). ‘N_ΔPolx_’ represents the subtraction of the numbers of total genomic rNMPs (four independent experiments; see Figure 5C and Supplementary Table S5) in RER-deficient strains bearing Pol α-L868M, δ-L612M or ε-M644G, either in presence of both Rnr1 and Top1 (samples 7, 13 and 19, respectively; condition i), or in absence of Rnr1 and presence of Top1 (samples 8, 14 and 20, respectively; condition ii), or in presence of Rnr1 and absence of Top1 (samples 9, 15 and 21, respectively; condition iii), or in absence of both Rnr1 and Top1 (samples 10, 16 and 22, respectively; condition iv), from the numbers of total genomic rNMPs in the corresponding RER-deficient strains counterparts bearing the three WT replicative Pols α, δ and ε (samples not indicated on the organigram), within the same gel/experiment. ‘F_Polx_’ represents the *in vitro* frequency of rNMP incorporation in DNA for purified Pols α-L868M, ε-M644G and δ-L612M i.e. 1 rNMP per 40, 100 and 300 dNMPs, respectively (frequencies from (19,20)). Symbols on the organigram: + and - indicate that the protein is present or absent, respectively; the alleles *pol1-L868M*, *pol3-L612M* and *pol2-M644G* are depicted in green, blue and red squares, respectively.

**
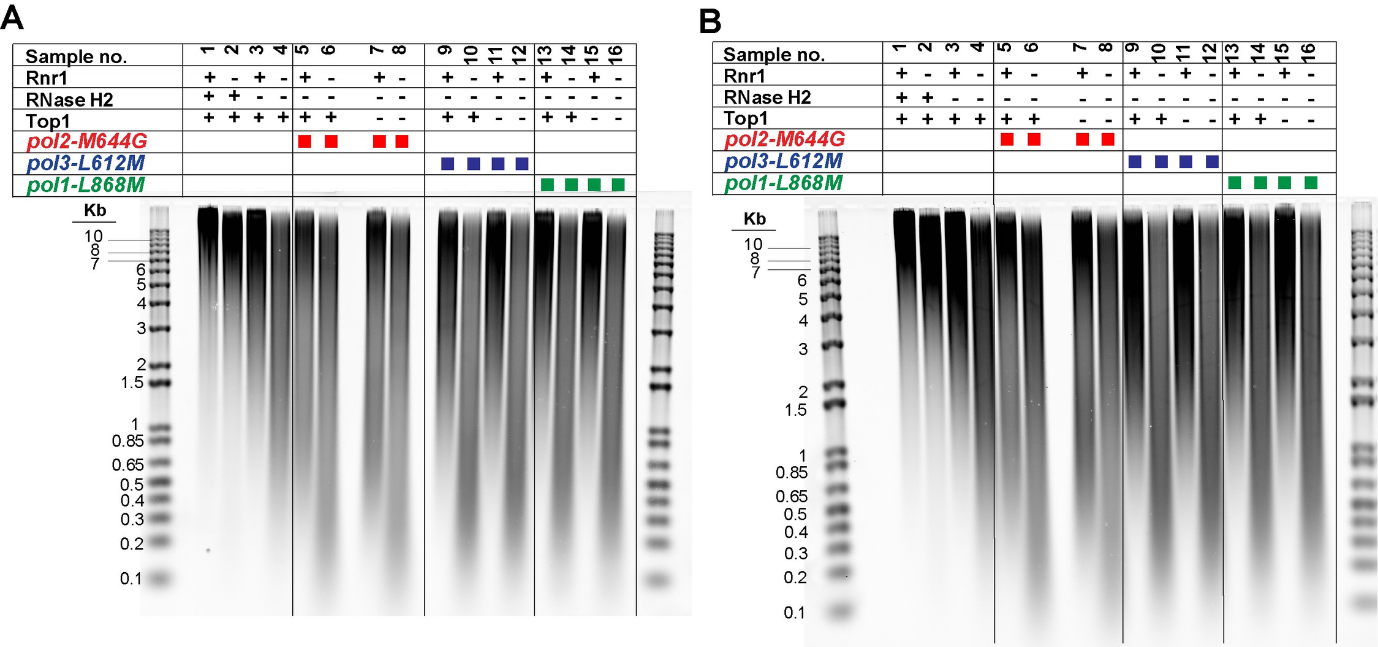
 Supplementary Figure S7. SYBR-stained gels for Southern-blots in Figure 6.** (**A**).SYBR-stained1% agarosegel for the Southern blot in Figure 6B. The vertical lines along the image of the gel are included for clarity. For other details, see legend of Figure 6. (**B**).SYBR-stained1% agarosegel for the Southern blot in Figure 6D. Other details are as for panel (A).

**
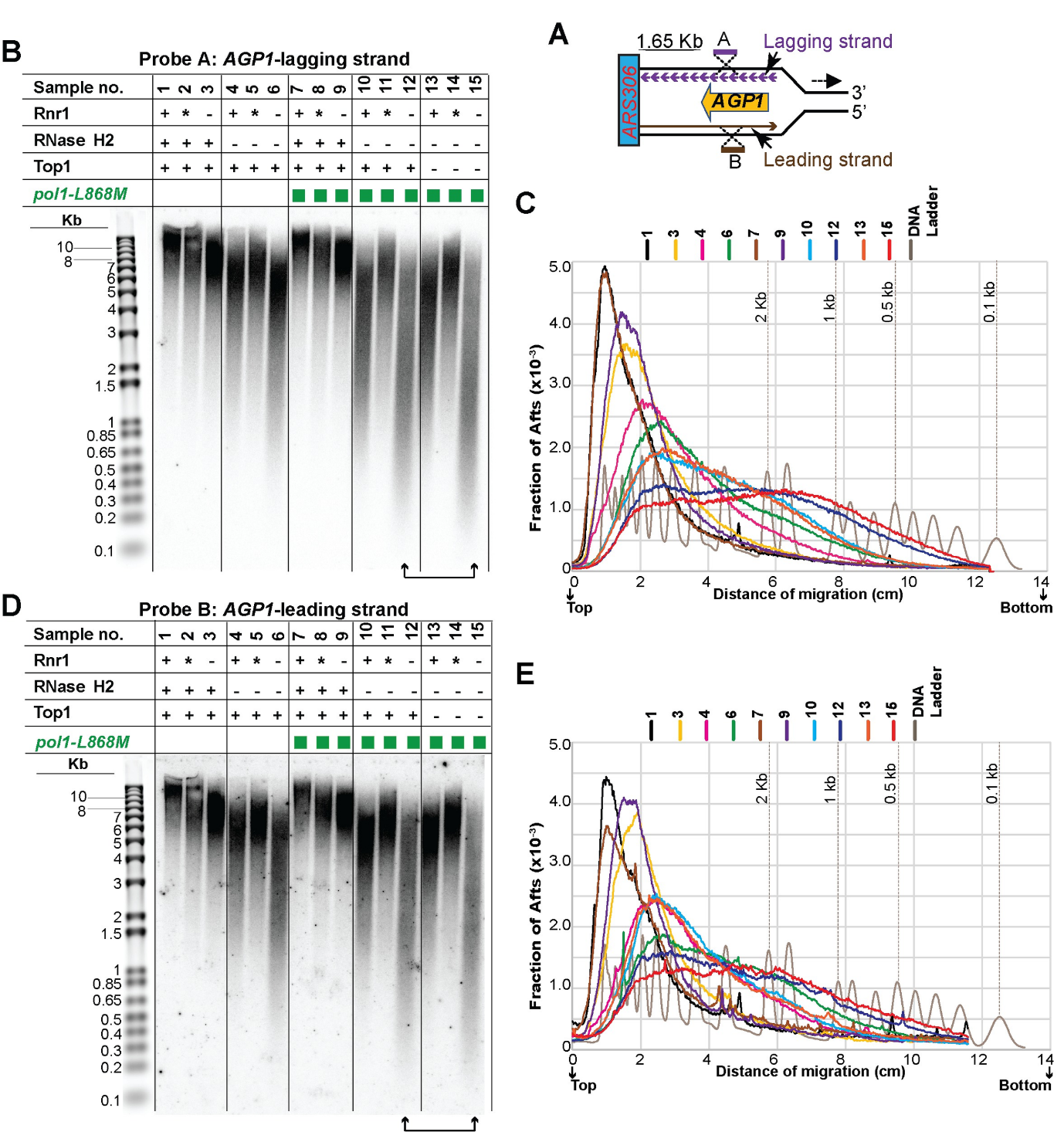
 Supplementary Figure S8. Southern analyses of RER-deficient Rnr1-depleted *TOP1^+^*/*top1Δ* mutants bearing Pol α-L868M**. This figure is related to Figure 6 and Supplementary Figures S9 and S10. (**A**).See Figure 6A. (**B**-**E**). This “introductory part” is common for Supplementary Figures S8-S10. For Rnr1 [+] condition (Rnr1 expression at WT levels), strains carrying the gene *RNR1* under the control of its native promoter were grown in rich YEPD (2% glucose) medium and harvested in exponential phase at OD_600_ ~0.5-0.6. For Rnr1 [*] condition (Rnr1 moderate over-expression), strains carrying *P_GAL_:3HA-RNR1* were grown in liquid minimal medium lacking histidine with 2% galactose and 2% sucrose at 30°C. An aliquot of cells growing in exponential phase was collected from this medium. For Rnr1 [-] condition (Rnr1 depletion), cells from the previous step were transferred to minimal medium lacking histidine with 2% glucose. Cells in exponential phase were harvested 6 hr after transfer to this medium (see also Material and Methods). Alkali-treated DNA samples (5 μg per lane) were separated on three alkaline 1% agarose gels, as follows: One gel for strains bearing allele *pol1-L868M* (samples 7-15 in Supplementary Figure S8), one gel for strains bearing *pol3-L612M* (samples 7-15 in Supplementary Figure S9), and one gel for strains bearing *pol2-M644G* (samples 7-15 in Supplementary Figure S10). As control, we included, in the three gels, DNA samples from strains WT (sample 1), *P_GAL_:3HA-RNR1* with Rnr1 that is either moderately over-expressed (sample 2) or depleted for 6 hr (sample 3), *rnh201Δ* (sample 4), and *P_GAL_:3HA-RNR1* *rnh201Δ* with Rnr1 that is either moderately over-expressed (sample 5) or depleted for 6 hr (sample 6). The gels were neutralized, stained with SYBR gold and capillary-blotted. In Supplementary Figures S8 and S9 the membrane was sequentially hybridized with probes A and B, and in Supplementary Figure S10 the membrane was sequentially hybridized with probes B and A (see also Material and Methods). The experiment was repeated independently twice. (**B**, **D**). Southern hybridizations of *AGP1*-lagging strand DNA with probe A in panel (B), and *AGP1-*leading strandDNA with probe B in panel (D). The following strains are represented by symbols on the organigrams (see also Supplementary Table S1 for the list of strains). Rnr1 [+] condition: 1. WT; 4. *rnh201*Δ; 7. *pol1-L868M*;10. *pol1-L868M rnh201*Δ; 13. *pol1-L868M rnh201*Δ* top1*Δ. Rnr1 [*] and Rnr1 [-] conditions, respectively: 2 and 3. *P_GAL_:3HA-RNR1*; 5 and 6. *P_GAL_:3HA-RNR1 rnh201*Δ; 8 and 9. *P_GAL_:3HA-RNR1 pol1-L868M*; 11 and 12. *P_GAL_:3HA-RNR1 pol1-L868M rnh201*Δ; 14 and 15. *P_GAL_:3HA-RNR1 pol1-L868M rnh201*Δ* top1*Δ. On the organigram: +, * and - indicate that the protein is present, moderately over-expressed or absent, respectively; the allele *pol1-L868M* is depicted by a green square on the organigram. For the ease of comparison, SYBR-stained DNA ladder from the picture of the gel (data not shown) was superimposed on the image of the blot. Selected molecular weights of the DNA ladder are labelled in Kb. The double-arrowed horizontal bar on the bottom of the blot points towards prominent differences between Top1+ and Top1- strains. The vertical lines along the image of the blot are included for clarity. (**C**, **E**). Signal densitometry histograms in (C) and (E) represent the quantifications of radioactive signals in selected samples in (B) and (D), respectively. For other details, see legend of Figure 6C.


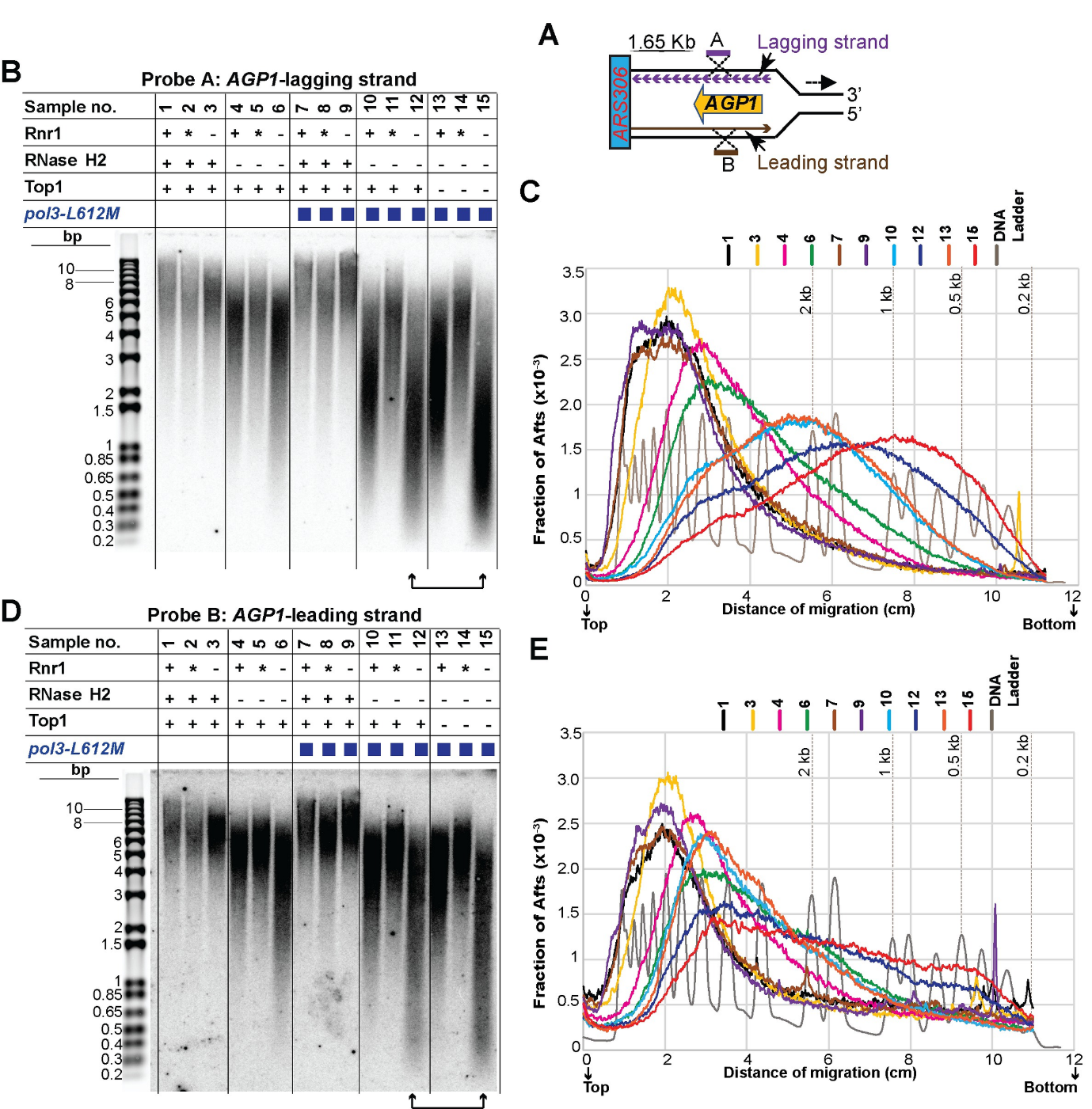


**Supplementary Figure S9. Southern analyses of RER-deficient Rnr1-depleted *TOP1^+^*/*top1Δ* mutants bearing Pol δ-L612M.** This figure is related to Figure 6 and Supplementary Figures S8 and S10. (**A**). See Figure 6A.(**B**-**E**). See the “introductory part”, which is common for Supplementary Figures S8-S10, in the legend of Supplementary Figure S8.(**B**, **D**). Southern hybridizations of *AGP1*-lagging strand DNA with probe A in panel (B), and *AGP1*-leading strand DNA with probe B in panel (D). The following strains are represented by symbols on the organigrams (see also Supplementary Table S1 for the list of strains). Rnr1 [+] condition: 1. WT; 4. *rnh201Δ*; 7. *pol3-L612M*;10. *pol3-L612M rnh201Δ*; 13. *pol3-L612M rnh201Δ top1Δ*. Rnr1 [*] and Rnr1 [-] conditions, respectively: 2 and 3. *P_GAL_:3HA-RNR1*; 5 and 6. *P_GAL_:3HA-RNR1 rnh201*Δ; 8 and 9. *P_GAL_:3HA-RNR1 pol3-L612M*; 11 and 12. *P_GAL_:3HA-RNR1 pol3-L612M rnh201*Δ; 14 and 15. *P_GAL_:3HA-RNR1 pol3-L612M rnh201*Δ* top1*Δ. The allele *pol3-L612M* is depicted by a blue square on the organigram. For other details, see legend of panels (B) and (D) in Supplementary Figure S8.(**C**, **E**). Signal densitometry histograms in (C) and (E) represent the quantifications of radioactive signals in selected samples in (B) and (D), respectively. For other details, see legend of Figure 6C.

**
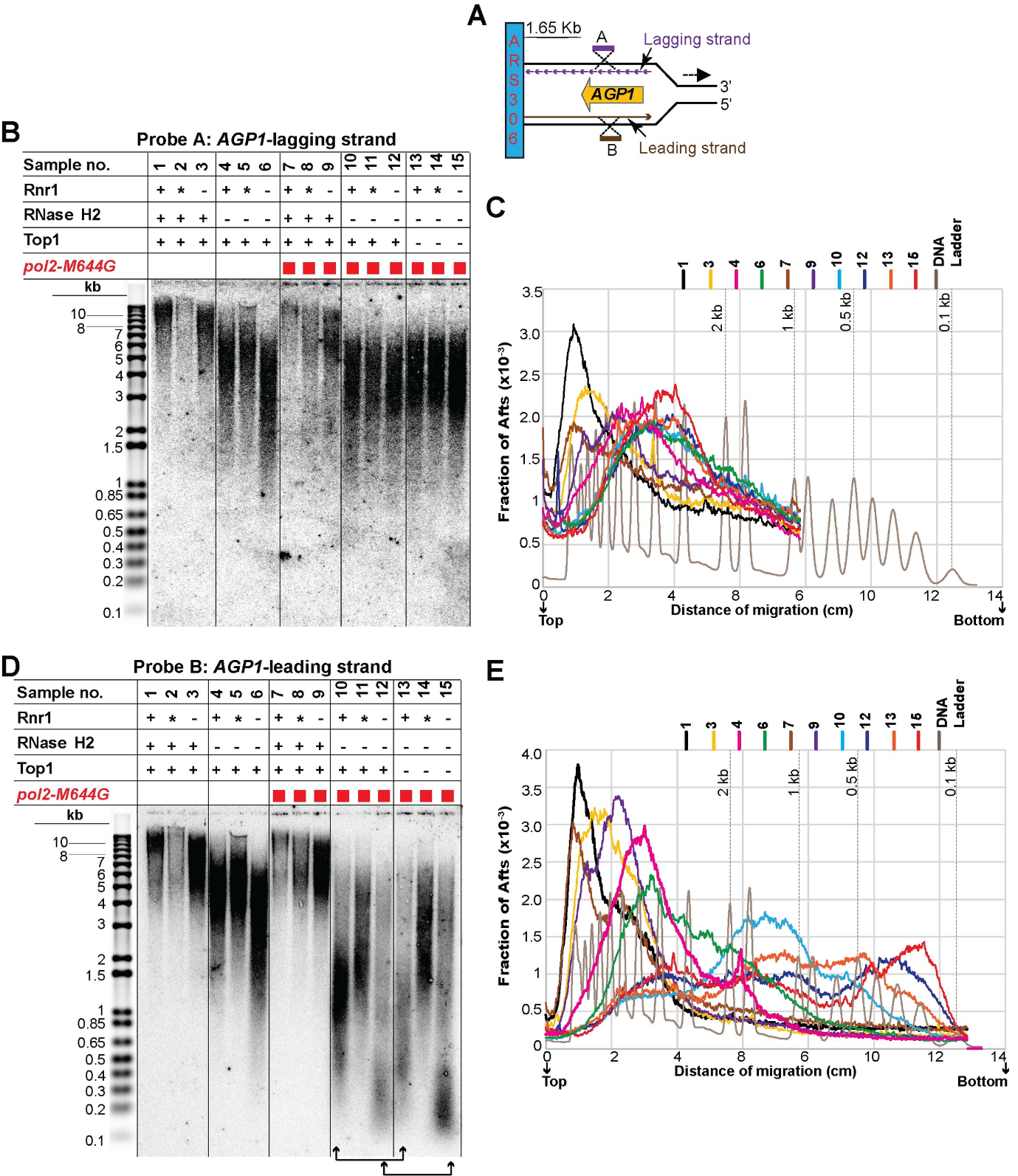
**

**Supplementary Figure S10. Southern analyses of RER-deficient Rnr1-depleted *TOP1^+^*/*top1Δ* mutants bearing Pol ε-M644G.** This figure is related to Figure 6 and Supplementary Figures S8 and S9. (**A**).See Figure 6A. (**B**-**E**). See the “introductory part”, which is common for Supplementary Figures S8-S10, in the legend of Supplementary Figure S8.(**B**, **D**). Southern hybridizations of *AGP1*-lagging strand DNA with probe A in panel (B), and *AGP1*-leading strand DNA with probe B in panel (D). The following strains are represented by symbols on the organigrams (see also Supplementary Table S1 for the list of strains). Rnr1 [+] condition: 1. WT; 4. *rnh201*Δ; 7. *pol2-M644G*;10. *pol2-M644G rnh201*Δ; 13. *pol2-M644G rnh201*Δ* top1*Δ. Rnr1 [*] and Rnr1 [-] conditions, respectively: 2 and 3. *P_GAL_:3HA-RNR1*; 5 and 6. *P_GAL_:3HA-RNR1 rnh201*Δ; 8 and 9. *P_GAL_:3HA-RNR1 pol2-M644G*; 11 and 12. *P_GAL_:3HA-RNR1 pol2-M644G rnh201*Δ; 14 and 15. *P_GAL_:3HA-RNR1 pol2-M644G rnh201*Δ* top1*Δ. The allele *pol2-M644G* is depicted by a red square on the organigram. For other details, see legend of panels (B) and (D) in Supplementary Figure S8. (**C**, **E**). Signal densitometry histograms in (C) and (E) represent the quantifications of radioactive signals in selected samples in (B) and (D), respectively. For other details, see legend of Figure 6C.

**
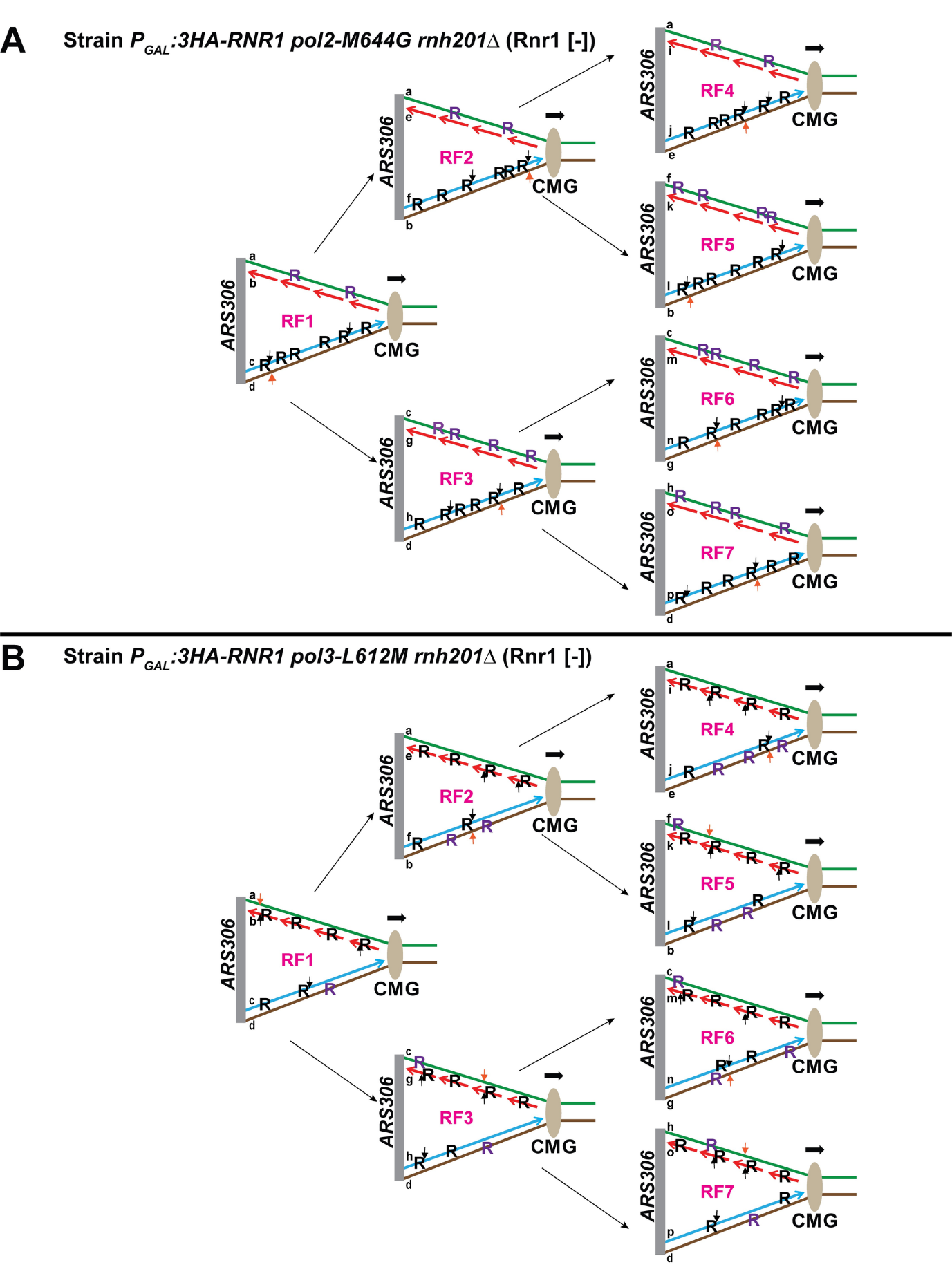
**

**Supplementary Figure S11. Model depicting single genomic rNMPs and the associated Top1-incisions in Rnr1-depleted RER-deficient triple mutants bearing Pol ε-M644G or δ-L612M.** (**A**, **B**). Represented is the RF that encompasses the locus *AGP1* (omitted for clarity), to the right side of the bidirectional origin *ARS306* on chromosome III. The direction of replication is indicated by a horizontal thick black arrow pointing right. Note that the RF to the left side of *ARS306* is omitted for clarity. CMG (Cdc45-MCM-GINS) is the replicative helicase complex (for reviews, see e.g. (21,22)). OFs on the nascent lagging strand, which are synthesized by Pols α and δ, are depicted by small red arrows. The nascent leading strand, which is mainly synthesized by Pol ε is depicted by one long blue arrow. Template leading and lagging strands are colored in brown and green, respectively. Represented are three rounds of DNA synthesis (S-phase) following Rnr1 depletion: RF1 for the first round, RF2 and RF3 for the second round, and RF4-RF7 for the third round. For example, RF1 generates one pair of dsDNA molecules/sister chromatids, which are labelled “ab” and “cd”; each DNA strand is given a Latin alphabet letter. dsDNA molecules/sister chromatids are segregated equally into mother and daughter cells by the end of mitosis. (**A**). Strain *P_GAL_:3HA-RNR1* *pol2-M644G* *rnh201Δ* (Rnr1 [-]). rNMPs incorporated by Pol ε-M644G in nascent leading strand of RF1 (and subsequent RFs) following Rnr1 depletion are depicted by black R. A subset of black R is incised by Top1 (black descending-arrow), thereby creating un-ligatable nicks. These can induce Top1-mediated DSBs (orange ascending-arrow on the complementary strand; see e.g. (23)), or other types of Top1-mediated RNA-DNA damage (omitted for clarity; for reviews, see e.g. (24-27)). Note that only cells that can overcome DNA damage can undergo another cell cycle/DNA synthesis (S-phase) round. Ribonucleotides that are inserted by Pol ε-M644G in nascent leading strand at the prior rounds of replication (before and after Rnr1 depletion), and which persist in DNA, are represented by violet Rs in template lagging strand. Note that violet Rs may not be cleaved by Top1 (for review, see e.g. (25)). rNMPs incorporated by WT Pols α and δ in nascent lagging strand following Rnr1 depletion are omitted for clarity. (**B**). Strain *P_GAL_:3HA-RNR1* *pol3-L612M* *rnh201Δ* (Rnr1 [-]). Similar description as for (A) with some modifications. rNMPs incorporated by Pol δ-L612M in both nascent leading and lagging strands of RF1 (and subsequent RFs) following Rnr1 depletion are depicted by black R. We depicted few black Rs in the leading strand because we found that rNMP incorporation is increased in *AGP1*-leading strand DNA in strain *P_GAL_:3HA-RNR1* *pol3-L612M* *rnh201Δ* (Rnr1 [-]) (see Figure 6 and Supplementary Figure S9). rNMPs incorporated by Pol δ-L612M in prior rounds of replication in nascent lagging strand (before and after Rnr1 depletion), and which persist in DNA, are depicted by violet R in template leading strand. rNMPs incorporated by Pol δ-L612M in prior rounds of replication in nascent leading strand (after Rnr1 depletion), and which persist in DNA, are depicted by violet R in template lagging strand. rNMPs incorporated by WT Pols α and ε following Rnr1 depletion are omitted for clarity.

**Supplementary Tables**

| **STRAINS** |  |  |
| --- | --- | --- |
| **Name** | **Genotype** | **Reference** |
| BY4741 | *MATa his3Δ1 leu2Δ0 met15Δ0 ura3Δ0* | Invitrogen |
| YAEH244^[a]^ | BY4741 but *rnh201Δ* (KanMx6) *top1Δ* (HphMx6) | (28) |
| YAEH255^[a]^ | BY4741 but *rnh1Δ* (KanMx6) *rnh201Δ* (NatMx6) | (28) |
| YAEH354^[a]^ | BY4741 but *rnh1Δ* (KanMx6) | (29) |
| YAEH355 | BY4741 but *rnh201Δ* (KanMx6) | (29) |
| YAEH544 | BY4741 but *dun1Δ* (His3MX6) | This study |
| YAEH562 | BY4741 but *P_GAL_:3HA-RNR1* (His3MX6) | This study |
| YAEH568 | as YAEH354 (*rnh1Δ*) but *P_GAL_:3HA-RNR1* (His3MX6) | This study |
| YAEH570 | as YAEH255 (*rnh1Δ,* *rnh201Δ*) but *P_GAL_:3HA-RNR1* (His3MX6) | This study |
| YAEH578 | as YAEH562 (*P_GAL_:3HA-RNR1*) but *top1Δ* (HphMX6) | This study |
| YAEH697 | as YAEH568 (*P_GAL_:3HA-RNR1*, *rnh1Δ*) but *rnh202Δ* (NatMX6) | This study |
| YAEH698 | as YAEH568 (*P_GAL_:3HA-RNR1*, *rnh1Δ*) but *rnh203Δ* (NatMX6) | This study |
| YAEH699^[a]^ | BY4741 but *rnh202Δ* (KanMX6) | Edinburgh Genome Foundry |
| YAEH700^[a]^ | BY4741 but *rnh203Δ* (KanMX6) | Edinburgh Genome Foundry |
| YAEH701 | as YAEH699 (*rnh202Δ*) but *P_GAL_:3HA-RNR1* (His3MX6) | This study |
| YAEH702 | as YAEH700 (*rnh203Δ*) but *P_GAL_:3HA-RNR1* (His3MX6) | This study |
| YAEH703 | as YAEH355 (*rnh201Δ*) but *P_GAL_:3HA-RNR1* (His3MX6) | This study |
| YAEH737 | BY4741 but *pol2-M644G* | This study |
| YAEH738 | BY4741 but *pol2-M644L* | This study |
| YAEH741 | as YAEH737 (*pol2-M644G*) but *rnh201Δ* (KanMX6) | This study |
| YAEH743 | as YAEH737 (*pol2-M644G*) but *P_GAL_:3HA-RNR1* (His3MX6) | This study |
| YAEH744 | as YAEH738 (*pol2-M644L*) but *P_GAL_:3HA-RNR1* (His3MX6) | This study |
| YAEH745 | as YAEH741 (*pol2-M644G*, *rnh201Δ*) but *P_GAL_:3HA-RNR1* (His3MX6) | This study |
| YAEH746 | as YAEH742 (*pol2-M644L*, *rnh201Δ*) but *P_GAL_:3HA-RNR1* (His3MX6) | This study |
| YAEH755 | as YAEH562 (*P_GAL_:3HA-RNR1*) but *dun1Δ* (HphMX6) | This study |
| YAEH756 | as YAEH737 (*pol2-M644G*) but *top1Δ* (HphMX6) | This study |
| YAEH757 | as YAEH741 (*pol2-M644G*, *rnh201Δ*) but *top1Δ* (HphMX6) | This study |
| YAEH764 | as YAEH756 (*pol2-M644G*, *top1Δ*) but *P_GAL_:3HA-RNR1* (His3MX6) | This study |
| YAEH765 | as YAEH757 (*pol2-M644G*, *top1Δ*, *rnh201Δ*) but *P_GAL_:3HA-RNR1* (His3MX6) | This study |
| YAEH770^[a]^ | as YAEH737 (*pol2-M644G*) but *rnh1Δ* (HphMX6) | This study |
| YAEH778 | as YAEH244 (*top1Δ*, *rnh201Δ*) but *P_GAL_:3HA-RNR1* (His3MX6) | This study |
| YAEH779^[a]^ | as BY4741 but *pol3-L612M* | This study |
| YAEH780 | as YAEH779 (*pol3-L612M*) but *rnh201Δ* (KanMX6) | This study |
| YAEH781 | as YAEH779 (*pol3-L612M*) but *P_GAL_:3HA-RNR1* (His3MX6) | This study |
| YAEH782 | as YAEH780 (*pol3-L612M*, *rnh201Δ*) but *P_GAL_:3HA-RNR1* (His3MX6) | This study |
| YAEH783 | as BY4741 but *sml1Δ* (NatMX6) | This study |
| YAEH784 | as YAEH355 (*rnh201Δ*) but *sml1Δ* (NatMX6) | This study |
| YAEH787 | As YAEH770 (*pol2-M644G* *rnh1Δ*) but *P_GAL_:3HA-RNR1* (His3MX6) | This study |
| YAEH789 | as YAEH741 (*pol2-M644G*, *rnh201Δ*) but *sml1Δ* (NatMX6) | This study |
| YAEH792 | as YAEH779 (*pol3-L612M*) but *top1Δ* (HphMX6) | This study |
| YAEH793 | as YAEH780 (*pol3-L612M*, *rnh201Δ*) but *top1Δ* (HphMX6) | This study |
| YAEH799 | as YAEH792 (*pol3-L612M*, *top1Δ*) but *P_GAL_:3HA-RNR1* (His3MX6) | This study |
| YAEH800 | as YAEH793 (*pol3-L612M*, *rnh201Δ*, *top1Δ*) but *P_GAL_:3HA-RNR1* (His3MX6) | This study |
| YAEH801 | as YAEH783 (*sml1Δ*) but *P_GAL_:3HA-RNR1* (His3MX6) | This study |
| YAEH805 | BY4741 but *pol1-L868M* | This study |
| YAEH808 | as YAEH805 (*pol1-L868M*) but *rnh201Δ* (KanMX6) | This study |
| YAEH809^[a]^ | as YAEH805 (*pol1-L868M*) but *top1Δ* (HphMX6) | This study |
| YAEH810 | as YAEH808 (*pol1-L868M*, *rnh201Δ*) but *top1Δ* (HphMX6) | This study |
| YAEH811 | as YAEH805 (*pol1-L868M*) but *P_GAL_:3HA-RNR1* (His3MX6) | This study |
| YAEH812 | as YAEH808 (*pol1-L868M*, *rnh201Δ*) but *P_GAL_:3HA-RNR1* (His3MX6) | This study |
| YAEH813 | as YAEH809 (*pol1-L868M*, *top1Δ*) but *P_GAL_:3HA-RNR1* (His3MX6) | This study |
| YAEH814 | as YAEH810 (*pol1-L868M*, *rnh201Δ*, *top1Δ*) but *P_GAL_:3HA-RNR1* (His3MX6) | This study |
| YAEH815 | BY4741 but *crt1Δ* (HphMX6) | This study |
| YAEH816 | as YAEH562 (*P_GAL_:3HA-RNR1*) but *crt1Δ* (HphMX6) | This study |
| YAEH817 | as YAEH355 (*rnh201Δ*) but *crt1Δ* (HphMX6) | This study |
| YAEH819 | as YAEH703 (*P_GAL_:3HA-RNR1*, *rnh201Δ*) but *crt1Δ* (HphMX6) | This study |
| YAEH820 | as YAEH743 (*pol2-M644G*, *P_GAL_:3HA-RNR1*) but *crt1Δ* (HphMX6) | This study |
| YAEH821 | as YAEH745 (*pol2-M644G*, *P_GAL_:3HA-RNR1*, *rnh201Δ*) but *crt1Δ* (HphMX6) | This study |
| YAEH822 | as YAEH741 (*pol2-M644G, rnh201Δ*) but *crt1Δ* (HphMX6) | This study |
| YAEH823 | as YAEH789 (*pol2M644G, rnh201Δ, sml1Δ*) but *crt1Δ* (HphMX6) | This study |
| YAEH824 | as YAEH817 (*rnh201Δ, crt1Δ*) but *sml1Δ* (NatMX6) | This study |
| YAEH825 | as YAEH781 (*pol3-L612M*, *P_GAL_:3HA-RNR1*) but *crt1Δ* (HphMX6) | This study |
| YAEH826 | as YAEH782 (*pol3-L612M*, *P_GAL_:3HA-RNR1*, *rnh201Δ*) but *crt1Δ* (HphMX6) | This study |
| YAEH827 | as YAEH811 (*pol1-L868M*, *P_GAL_:3HA-RNR1*) but *crt1Δ* (HphMX6) | This study |
| YAEH828 | as YAEH812 (*pol1-L868M*, *P_GAL_:3HA-RNR1*, *rnh201Δ*) but *crt1Δ* (HphMX6) | This study |
| YAEH829 | as YAEH570 (*P_GAL_:3HA-RNR1, rnh201Δ*, *rnh1Δ*) but *crt1Δ* (HphMX6) | This study |
| **PLASMIDS** |  |  |
| **Name** | **Specifications** | **Reference** |
| Vector | ycplac111: *LEU2*, *ARS/CEN* | Novagen |
| p-*RNH201* | ycNPH2‐FL2: C‐terminally 2x FLAG‐tagged *RNH201* gene under the control of its own native promoter in ycplac111 | (30) |
| p-*rnh201-G42S* | As ycNPH2‐FL2 but carrying *rnh201-G42S* | (30) |
| p-*rnh201-RED* | As ycNPH2‐FL2 but carrying *rnh201-P45D-Y219A* | (30) |

**Supplementary Table S1**. **List of strains and plasmids.** **^[a]^** Strain that is not represented in Figures and/or Supplementary Figures, but that was used for the construction of its strain(s) derivative(s), which is(are) represented in Figures and/or Supplementary Figures and in this table.

| **Primer** | **Sequence (5’- 3’)** | **Specifications** |
| --- | --- | --- |
| **Primers for synthesis of *AGP1* double-stranded PCR amplicon** |  |  |
| AGP1-F1 | GCATCCATGACTATCAAATA | Coordinates of *AGP1* on chr III are:  77,919-76,108. Coordinates of ds PCR are: 77,259-76,479. |
| AGP1-R1 | AGTGAAAACTTGTTCTTCCT | Coordinates of *AGP1* on chr III are:  77,919-76,108. Coordinates of ds PCR are: 77,259-76,479. |
| **Primers for synthesis of *AGP1* single-stranded (ss) probes** |  |  |
| AGP1-A | AGCGTAACCTTGCTCGGATA | Detects *AGP1* lagging-strand*. ARS306* coordinates on chr III are  74458-74677. Coordinates of the ss probe are: 76,479-77,139 (i.e. ~ at a distance of 1,802 bp from *ARS306*). |
| AGP1-B | TTCTTCTTCAACTGTTGCAA | Detects *AGP1* leading-strand. *ARS306* coordinates on chr III are  74458-74677. Coordinates of the ss probe are: 77,260-76,601 (i.e. ~ at a distance of 1,924 bp from *ARS306*). |
| **Primers for qPCR** |  |  |
| RNR1-F | TCATGAAGCATGGTGTTAGA |  |
| RNR1-R | GACGGGAGTACATATTGGAA |  |
| RNR2-F | CATCAAGGACCCTAAAGAAA |  |
| RNR2-R | AGGCAACTAGTCTTTCACCA |  |
| RNR3-F | CTCAACTTCCCAAATTCTTG |  |
| RNR3-R | ACTTGGAATTCACCAGACAG |  |
| RNR4-F | TGTTCTGGTTGACTGACAAA |  |
| RNR4-R | GTTCTCAAATGGGCAAATAG |  |
| HUG1-F | TGTCAAGACCGGCTACTTAT |  |
| HUG1-R | CTTACCAATGTCAGAAAGACC |  |
| CRT1-F | ACGCTTCATCTTGTTCCCAA |  |
| CRT1-R | TACCCAGCCTTCTAGTCGTT |  |
| ACT1-F | TTGGATTCCGGTGATGGTGT |  |
| ACT1-R | CGGCCAAATCGATTCTCAAA |  |

| **Primer** | **Sequence (5’- 3’)** | **Specifications** |
| --- | --- | --- |
| **Primers for synthesis of *CAN1* PCR amplicon** |  |  |
| BC3164-Forward | GACAATTCAAAAGAAGACGCCGACAT |  |
| BC3165-Reverse | CAACATTCCAAAATTTGTCCC |  |
| **Primers for sequencing of *CAN1* PCR amplicon** |  |  |
| BC3161 | CAAATTCAAAAGAAGACG |  |
| BC3162 | GGCCAATGGTTACATGTA |  |
| BC3163 | GCTCTCTATTATTCATTG |  |

**Supplementary Table S2. List of oligonucleotides.**

| Sample no.  and  Genotype | Total mutation rate  x 10^-8 [a]^ | F.I. | Transitions  rate  x 10^-8^ ^[b]^ | | F.I. | Transversions  rate  x 10^-8 [b]^ | F.I. | 1 bp indel  rate  x 10^-8^ ^[b]^ | F.I. | Δ2-5 bp  rate  x 10^-8 [b]^ | F.I. |
| --- | --- | --- | --- | --- | --- | --- | --- | --- | --- | --- | --- |
| 1.  Wild-type | 7.3  (4.1-  9.4) | 1 | 1.4  (12/64) | 1 | | 4.2  (37/64) | 1 | 1.2  (11/64) | 1 | 0.1  (1/64) | 1 |
| 2.  *rnh201*Δ | 17.9 (9.3-68.1) | 2.4 | 3.8  (12/57) | 2.7 | | 6.9  (22/57) | 1.6 | 4.7  (15/57) | 3.7 | 2.2  (7/57) | 19.3 |
| 3.  *P_GAL_:3HA-RNR1* | 14.5 (9.7-25.4) | 2.0 | 5.0  (33/95) | 3.7 | | 6.7  (44/95) | 1.6 | 2.1  (14/95) | 1.7 | 0.5  (3/95) | 4.0 |
| *4. P_GAL_:3HA-RNR1 rnh201*Δ | 168.2 (143-198.7) | 23.0 | 16.0  (10/105) | 11.7 | | 27.2  (17/105) | 6.4 | 6.4  (4/105) | 5.1 | 118.5 (74/105) | 1039.3 |

**Supplementary Table S3. Values of total and specific *CAN1* mutation rates.** WT strain (sample 1), single mutant *rnh201Δ* (sample 2), single mutant *P_GAL_:3HA-RNR1* (sample 3),and double mutant *P_GAL_:3HA-RNR1* *rnh201Δ* (sample 4), were grown in rich YEPD (2% glucose) solid medium (see Material and Methods). In these growth conditions, Rnr1 should be expressed at WT levels in samples 1 and 2, and Rnr1 should be depleted in samples 3 and 4. F.I. stands for ‘Fold increase relative to the WT strain’. **^[a]^** Total mutation rates and 95% confidence intervals (indicated in parenthesis) were calculated by the Lea and Coulson method of median (31,32). **^[b]^** Specific mutation rates were calculated using the formula [specific mutation rate within a given strain= (number of sequenced *CAN1* mutants with specific mutation/ total number of sequenced *CAN1* mutants) x total mutation rate; see (33)]. Numbers in parenthesis represent the number of *CAN1* mutants with a specific mutation versus the total number of sequenced *CAN1* mutants, within a given strain (see also Material and Methods). 1 bp Indel: 1 base pair insertion/deletion; Δ2-5 bp: 2-5 base pairs deletion.

**Supplementary References**

1. Tsaponina, O., Barsoum, E., Astrom, S.U. and Chabes, A. (2011) Ixr1 is required for the expression of the ribonucleotide reductase Rnr1 and maintenance of dNTP pools. *PLoS Genet*, **7**, e1002061.

2. Sanvisens, N., de Llanos, R. and Puig, S. (2013) Function and regulation of yeast ribonucleotide reductase: cell cycle, genotoxic stress, and iron bioavailability. *Biomed J*, **36**, 51-58.

3. Huang, M., Zhou, Z. and Elledge, S.J. (1998) The DNA replication and damage checkpoint pathways induce transcription by inhibition of the Crt1 repressor. *Cell*, **94**, 595-605.

4. Meurisse, J., Bacquin, A., Richet, N., Charbonnier, J.B., Ochsenbein, F. and Peyroche, A. (2014) Hug1 is an intrinsically disordered protein that inhibits ribonucleotide reductase activity by directly binding Rnr2 subunit. *Nucleic Acids Res*, **42**, 13174-13185.

5. Chabes, A., Domkin, V. and Thelander, L. (1999) Yeast Sml1, a protein inhibitor of ribonucleotide reductase. *J Biol Chem*, **274**, 36679-36683.

6. Lee, Y.D., Wang, J., Stubbe, J. and Elledge, S.J. (2008) Dif1 is a DNA-damage-regulated facilitator of nuclear import for ribonucleotide reductase. *Mol Cell*, **32**, 70-80.

7. Lee, Y.D. and Elledge, S.J. (2006) Control of ribonucleotide reductase localization through an anchoring mechanism involving Wtm1. *Genes Dev*, **20**, 334-344.

8. Wu, X., An, X., Zhang, C. and Huang, M. (2018) Clb6-Cdc28 Promotes Ribonucleotide Reductase Subcellular Redistribution during S Phase. *Mol Cell Biol*, **38**.

9. Earp, C., Rowbotham, S., Merenyi, G., Chabes, A. and Cha, R.S. (2015) S phase block following MEC1ATR inactivation occurs without severe dNTP depletion. *Biol Open*, **4**, 1739-1743.

10. Zhao, X. and Rothstein, R. (2002) The Dun1 checkpoint kinase phosphorylates and regulates the ribonucleotide reductase inhibitor Sml1. *Proc Natl Acad Sci U S A*, **99**, 3746-3751.

11. Andreson, B.L., Gupta, A., Georgieva, B.P. and Rothstein, R. (2010) The ribonucleotide reductase inhibitor, Sml1, is sequentially phosphorylated, ubiquitylated and degraded in response to DNA damage. *Nucleic Acids Res*, **38**, 6490-6501.

12. Li, X., Jin, X., Sharma, S., Liu, X., Zhang, J., Niu, Y., Li, J., Li, Z., Zhang, J., Cao, Q. *et al.* (2019) Mck1 defines a key S-phase checkpoint effector in response to various degrees of replication threats. *PLoS Genet*, **15**, e1008136.

13. Ding, J., Taylor, M.S., Jackson, A.P. and Reijns, M.A. (2015) Genome-wide mapping of embedded ribonucleotides and other noncanonical nucleotides using emRiboSeq and EndoSeq. *Nat Protoc*, **10**, 1433-1444.

14. El Hage, A. and Tollervey, D. (2018) Immunoprecipitation of RNA:DNA Hybrids from Budding Yeast. *Methods Mol Biol*, **1703**, 109-129.

15. Ramirez, M., Velazquez, R., Maqueda, M., Lopez-Pineiro, A. and Ribas, J.C. (2015) A new wine Torulaspora delbrueckii killer strain with broad antifungal activity and its toxin-encoding double-stranded RNA virus. *Front Microbiol*, **6**, 983.

16. Sekiguchi, J. and Shuman, S. (1997) Site-specific ribonuclease activity of eukaryotic DNA topoisomerase I. *Mol Cell*, **1**, 89-97.

17. Rydberg, B. and Game, J. (2002) Excision of misincorporated ribonucleotides in DNA by RNase H (type 2) and FEN-1 in cell-free extracts. *Proc Natl Acad Sci U S A*, **99**, 16654-16659.

18. Reijns, M.A.M., Kemp, H., Ding, J., de Proce, S.M., Jackson, A.P. and Taylor, M.S. (2015) Lagging-strand replication shapes the mutational landscape of the genome. *Nature*, **518**, 502-506.

19. Nick McElhinny, S.A., Kumar, D., Clark, A.B., Watt, D.L., Watts, B.E., Lundstrom, E.B., Johansson, E., Chabes, A. and Kunkel, T.A. (2010) Genome instability due to ribonucleotide incorporation into DNA. *Nat Chem Biol*, **6**, 774-781.

20. Williams, J.S., Clausen, A.R., Lujan, S.A., Marjavaara, L., Clark, A.B., Burgers, P.M., Chabes, A. and Kunkel, T.A. (2015) Evidence that processing of ribonucleotides in DNA by topoisomerase 1 is leading-strand specific. *Nat Struct Mol Biol*, **22**, 291-297.

21. Burgers, P.M.J. and Kunkel, T.A. (2017) Eukaryotic DNA Replication Fork. *Annu Rev Biochem*, **86**, 417-438.

22. Kunkel, T.A. and Burgers, P.M.J. (2017) Arranging eukaryotic nuclear DNA polymerases for replication: Specific interactions with accessory proteins arrange Pols alpha, delta, and epsilon in the replisome for leading-strand and lagging-strand DNA replication. *Bioessays*, **39**.

23. Huang, S.N., Williams, J.S., Arana, M.E., Kunkel, T.A. and Pommier, Y. (2017) Topoisomerase I-mediated cleavage at unrepaired ribonucleotides generates DNA double-strand breaks. *EMBO J*, **36**, 361-373.

24. Ashour, M.E., Atteya, R. and El-Khamisy, S.F. (2015) Topoisomerase-mediated chromosomal break repair: an emerging player in many games. *Nat Rev Cancer*, **15**, 137-151.

25. Cho, J.E. and Jinks-Robertson, S. (2017) Ribonucleotides and Transcription-Associated Mutagenesis in Yeast. *J Mol Biol*, **429**, 3156-3167.

26. Cho, J.E. and Jinks-Robertson, S. (2018) Topoisomerase I and Genome Stability: The Good and the Bad. *Methods Mol Biol*, **1703**, 21-45.

27. Williams, J.S., Lujan, S.A. and Kunkel, T.A. (2016) Processing ribonucleotides incorporated during eukaryotic DNA replication. *Nat Rev Mol Cell Biol*, **17**, 350-363.

28. El Hage, A., French, S.L., Beyer, A.L. and Tollervey, D. (2010) Loss of Topoisomerase I leads to R-loop-mediated transcriptional blocks during ribosomal RNA synthesis. *Genes Dev*, **24**, 1546-1558.

29. El Hage, A., Webb, S., Kerr, A. and Tollervey, D. (2014) Genome-wide distribution of RNA-DNA hybrids identifies RNase H targets in tRNA genes, retrotransposons and mitochondria. *PLoS Genet*, **10**, e1004716.

30. Chon, H., Sparks, J.L., Rychlik, M., Nowotny, M., Burgers, P.M., Crouch, R.J. and Cerritelli, S.M. (2013) RNase H2 roles in genome integrity revealed by unlinking its activities. *Nucleic Acids Res*, **41**, 3130-3143.

31. Lea, D.E. and Coulson, C.A. (1949) The distribution of the numbers of mutants in bacterial populations. *J Genet*, **49**, 264-285.

32. Spell, R.M. and Jinks-Robertson, S. (2004) Determination of mitotic recombination rates by fluctuation analysis in Saccharomyces cerevisiae. *Methods Mol Biol*, **262**, 3-12.

33. Ghodgaonkar, M.M., Lazzaro, F., Olivera-Pimentel, M., Artola-Boran, M., Cejka, P., Reijns, M.A., Jackson, A.P., Plevani, P., Muzi-Falconi, M. and Jiricny, J. (2013) Ribonucleotides misincorporated into DNA act as strand-discrimination signals in eukaryotic mismatch repair. *Mol Cell*, **50**, 323-332.
